# Closed-loop vagus nerve stimulation aids recovery from spinal cord injury

**DOI:** 10.1038/s41586-025-09028-5

**Published:** 2025-05-21

**Authors:** Michael P. Kilgard, Joseph D. Epperson, Emmanuel A. Adehunoluwa, Chad Swank, Amy L. Porter, David T. Pruitt, Holle L. Gallaway, Christi Stevens, Jaime Gillespie, Dannae Arnold, Mark B. Powers, Rita G. Hamilton, Richard C. Naftalis, Michael L. Foreman, Jane G. Wigginton, Seth A. Hays, Robert L. Rennaker

**Affiliations:** 1 Texas Biomedical Device Center, The University of Texas at Dallas, Richardson, TX USA; 2 Department of Neuroscience, School of Behavioral and Brain Sciences, The University of Texas at Dallas, Richardson, TX USA; 3 Department of Bioengineering, Erik Jonsson School of Engineering and Computer Science, The University of Texas at Dallas, Richardson, TX USA; 4 Baylor Scott and White Research Institute, Dallas, TX USA; 5 Baylor Scott and White Institute for Rehabilitation, Dallas, TX USA; 6 Division of Trauma, Baylor University of Medical Center, Dallas, TX USA; 7 Department of Neurosurgery, Baylor University of Medical Center, Dallas, TX USA; 8 Division of Acute Care Surgery, Baylor University Medical Center, Dallas, TX USA; 9 XNerve Medical Inc., Plano, TX USA

**Keywords:** Diseases of the nervous system, Motor control

## Abstract

Decades of research have demonstrated that recovery from serious neurological injury will require synergistic therapeutic approaches. Rewiring spared neural circuits after injury is a long-standing goal of neurorehabilitation^1,2^. We hypothesized that combining intensive, progressive, task-focused training with real-time closed-loop vagus nerve stimulation (CLV) to enhance synaptic plasticity^3^ could increase strength, expand range of motion and improve hand function in people with chronic, incomplete cervical spinal cord injury. Here we report the results from a prospective, double-blinded, sham-controlled, randomized study combining gamified physical therapy using force and motion sensors to deliver sham or active CLV (ClinicalTrials.gov identifier NCT04288245). After 12 weeks of therapy composed of a miniaturized implant selectively activating the vagus nerve on successful movements, 19 people exhibited a significant beneficial effect on arm and hand strength and the ability to perform activities of daily living. CLV represents a promising therapeutic avenue for people with chronic, incomplete cervical spinal cord injury.

## Abstract (web summary)

Following spinal cord injury, gamified physical therapy that incorporates closed-loop vagus nerve stimulation can aid restoration of arm and hand function.

## Main

Single therapies have demonstrated limited success in promoting recovery in people with chronic neurological injury. It has become increasingly apparent that the combination of complementary interventions represents a more promising strategy, such as recent applications that use electrical stimulation during rehabilitation to facilitate engagement of weakened networks^4–6^. CLV integrates targeted, intensive rehabilitative training with precisely timed vagus nerve stimulation (VNS) to promote recovery^7,8^. Rehabilitative training produces neural activation in spared pathways that control target musculature, which promotes plasticity through canonical mechanisms^9,10^, but is typically insufficient to support meaningful recovery on its own. CLV combines this training with VNS, which drives rapid, phasic release of acetylcholine, noradrenaline and serotonin throughout the central nervous system^11–15^. The resultant engagement of neuromodulatory networks within the seconds-long synaptic eligibility trace serves to enhance plasticity in networks activated by rehabilitation^16^. Conceptually, this approach of triggering stimulation to influence neuromodulatory networks during rehabilitation differentiates CLV from other combinatorial strategies that seek to directly facilitate the circuits engaged in movement^6^. In CLV, VNS and rehabilitation operate synergistically to mitigate the notoriously difficult credit assignment problem of identifying which synapses in a damaged network should be modified to produce more effective recovery of motor control^10,17^.

CLV was initially developed and refined in a range of animal models, including traumatic incomplete spinal cord injury (SCI)^3,18–24^. These studies have revealed that the combination of high-intensity rehabilitative training with real-time VNS produces synaptic plasticity in motor control networks in the cortex, subcortical structures and spinal cord to engender functional recovery that is not possible with training alone. Concurrent implementation is a key component, as decoupling the delivery of VNS by even tens of seconds from the appropriate movements fails to promote recovery, consistent with the reliance on the synaptic eligibility trace^3,25^. Moreover, efficacy is dependent on hundreds of VNS–movement pairings per day for many weeks^26,27^.

**Fig. 1:**
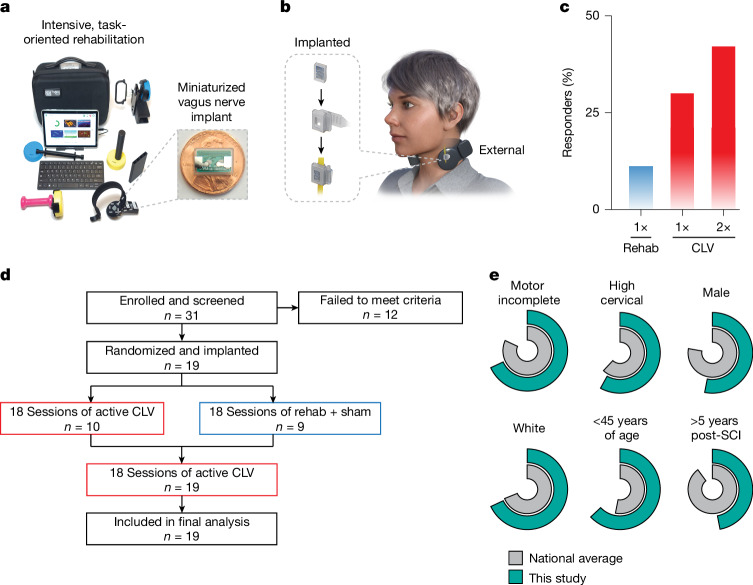
Closed-loop VNS for SCI. **a**, CLV combines intensive, task-oriented rehabilitation and concurrent VNS delivered with a miniaturized implanted stimulator to promote adaptive changes in the central nervous system and facilitate recovery of function. **b**, CLV integrates external and implanted hardware to deliver high-intensity arm and hand therapy enhanced by real-time neuromodulation. The miniaturized implant is powered and controlled by an external device placed on the neck during rehabilitation sessions. A suite of sensors enables the rehabilitation software to provide continuous visual feedback of hand position and force production during individualized rehabilitative exercises and facilitate real-time delivery of VNS during above average movements. **c**, In this study, we observed that CLV (red) produced accumulating improvements in upper limb recovery exceeding those with intensive rehabilitation with sham stimulation (blue). **d**, CONSORT diagram summarizing the enrolment stages and the per protocol completion rate (detailed in Extended Data Fig. 1). A description of reasons participants did not meet criteria can be found in Extended Data Table 2. All participants that met enrolment criteria were implanted and completed the study per protocol, which involved 42 visits total, including 36 physical therapy sessions. Participants were block randomized to receive 18 sessions of rehabilitation with either active CLV (1× CLV) or sham stimulation (1× rehab) during the RCT phase, followed by an additional 18 sessions of rehabilitation with active CLV regardless of previous group assignment. Participants that received 36 sessions of rehabilitation with active CLV are labelled as 2× CLV. This design allows comparison with sham stimulation and of two dosing regimens of CLV. **e**, The proportion of injury type, ethnicity and age of individuals in this study are comparable with averages reported by the National Spinal Cord Injury Statistical Center.

**Extended Data Fig. 1:**
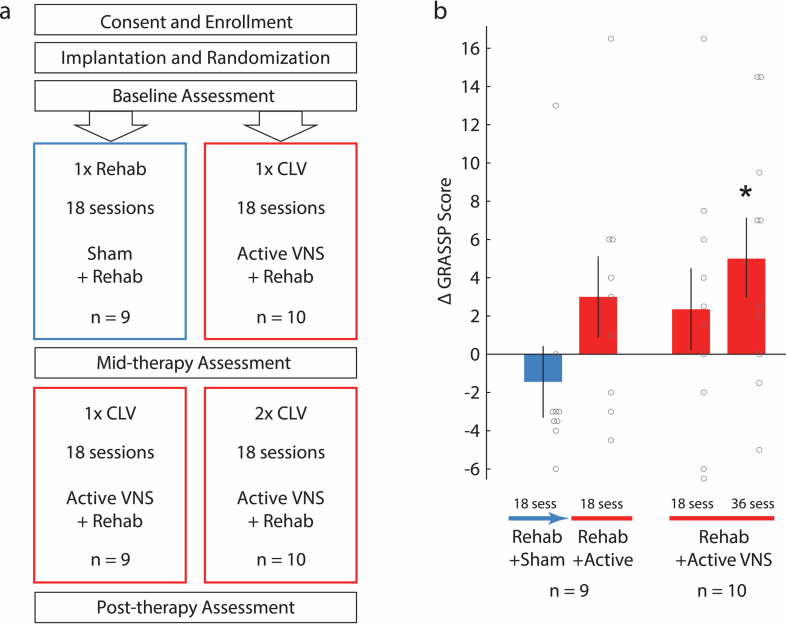
CLV Study design. (a) The study was designed to establish safety and feasibility as well as to estimate the effect of closed-loop delivery of VNS during high-intensity physical therapy compared to high-intensity physical therapy alone. After enrollment, all subjects were implanted with the VNS device. Participants were block randomized to receive 18 sessions of rehabilitation with either active CLV (1x CLV; n = 10 participants) or sham stimulation (1x Rehab; n = 9 participants) during the RCT phase, followed by an additional 18 sessions of rehabilitation with active CLV regardless of prior group assignment. Participants that received 36 sessions of rehabilitation with active CLV are labelled as 2x CLV. This design allows comparison with sham stimulation and of two dosing regimens of CLV. Assessments were completed before and after surgery, after eighteen therapy sessions, and after thirty-six therapy sessions. The final assessment was conducted 9.7 ± 2.1 days after the last day of VNS. (b) Depiction of change in GRASSP scores from baseline, the main functional outcome of the study, at each assessment during the RCT. Gray markers indicate individual participants. Paired Student’s t-test v. baseline; * indicates p < 0.05; data presented as mean ± SEM.

We sought to evaluate this approach in humans with chronic, incomplete SCI. To do so, we developed an integrated two-element system to govern both high-intensity rehabilitation and real-time neuromodulation (Fig. 1a). The first element utilizes devices instrumented to collect a range of movement parameters to allow each user to control an individualized set of video games incorporating dozens of different exercises to target specific muscles with limited function^28^. Difficulty was continuously adjusted so that the speed, force and range of motion needed to control gameplay were individualized to the peak performance abilities of each participant throughout the course of therapy. The second element, a miniaturized implanted VNS device, delivers concurrent real-time neuromodulation. The implanted stimulator^29^, which is 50 times smaller than existing systems and implanted via a simplified procedure, was wirelessly activated by a therapist or the gameplay software using a low-latency, adaptive real-time algorithm to deliver stimulation concurrent with movements that best approximate the desired outcome^30^ (Fig. 1b). After integrating these components and gaining regulatory approval, including investigational device exemption and designation as a breakthrough device from the FDA, we performed a double-blinded, sham-controlled clinical trial to test the hypothesis that CLV could enhance recovery of arm and hand function in 19 individuals with chronic, incomplete cervical SCI (Fig. 1d and Extended Data Fig. 1).

## Participants mirror national demographics

Recruiting participants that reflect the demographics of the broader clinical population is crucial to the generalizability of study results^31,32^. This trial followed the recommendations of the International Campaign for Cures of Spinal Cord Injury Paralysis panel^33^ and succeeded in enrolling a reasonably diverse set of participants whose injury type, ethnicity and age were generally consistent with the national averages reported by the National Spinal Cord Injury Statistical Center (Fig. 1d,e and Extended Data Tables 1 and 2). This study had a somewhat higher proportion of female individuals and individuals within 5 years since injury. There is evidence that interest in clinical trials may lessen with time after injury^34^, which could explain why we did not recruit a larger proportion of individuals who were more than 5 years post-injury. The 19 people who met the study criteria ranged considerably in age (21–65 years of age, median 40), time since injury (13–541 months, median 54), injury severity classification (American Spinal Injury Association Impairment Scale (AIS) B, C and D) and resultant arm and hand deficits (33–101 GRASSP, median 64). This diverse study population allows for initial exploration of the impact of demographic factors that could influence response to CLV.

## Hand strength is a viable therapy target

**Fig. 2:**
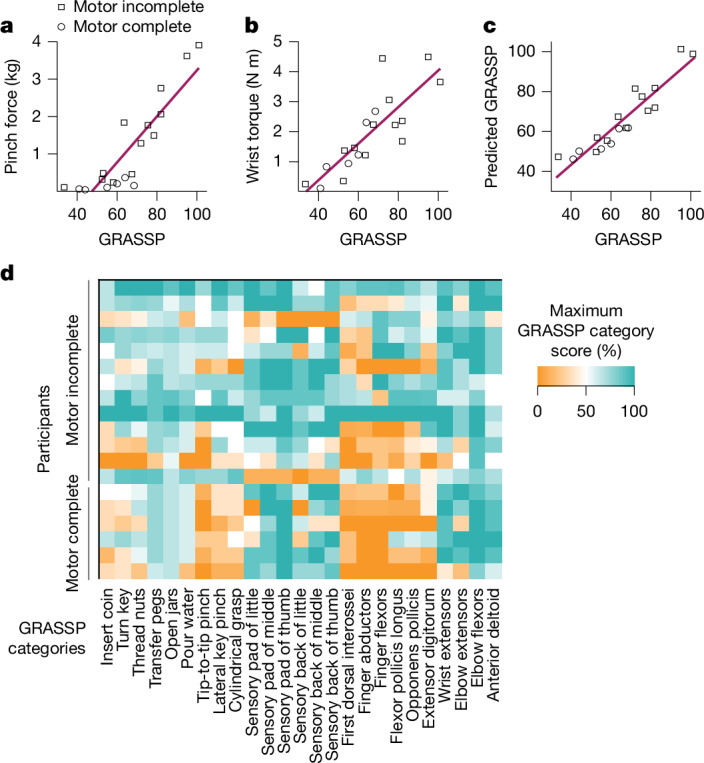
Hand and wrist dysfunction after SCI represent targets for improved functional recovery. **a**,**b**, Clinical impairment as measured by GRASSP is well correlated with pinch force (**a**) and wrist torque (**b**). **c**, Both metrics make statistically significant contributions to a linear model that accounts for most of the variance in clinical impairment. The strong correlation between clinical impairment and distal strength supports the selection of rehabilitative exercises designed to target these muscles. **d**, Each participant displayed a unique set of impairments as measured by the GRASSP score. The Pearson correlation coefficient between individuals was 0.34 ± 0.35 (mean ± s.d.). The colour represents the degree of function on each of the 25 components of the GRASSP assessment. This diversity in function motivated the creation of individualized rehabilitative regimens.

Our baseline assessments confirmed earlier studies showing that clinical impairment in upper limb function as measured by the Graded and Redefined Assessment of Strength, Sensibility and Prehension (GRASSP) score is highly correlated with the peak pinch force that participants could produce between their thumb and index finger^35^ (Pearson correlation, *R*^2^ = 0.77, *P* = 1 × 10^−6^; Fig. 2a). GRASSP was also well correlated with the peak torque participants could produce with their wrist (Pearson correlation, *R*^2^ = 0.68, *P* = 1×10^−5^; Fig. 2b). The linear combination of these two measures of hand strength produced a model that explains 87% of the variance in disability in this population, with each factor providing a statistically significant contribution to the model (Pearson correlation, *P* = 7 × 10^−9^; Fig. 2c). The strong association of clinical impairment with finger and wrist strength provides a rigorous therapeutic rationale for the use of exercises designed to target these muscles.

## Progressive, intensive and personalized CLV

**Fig. 3:**
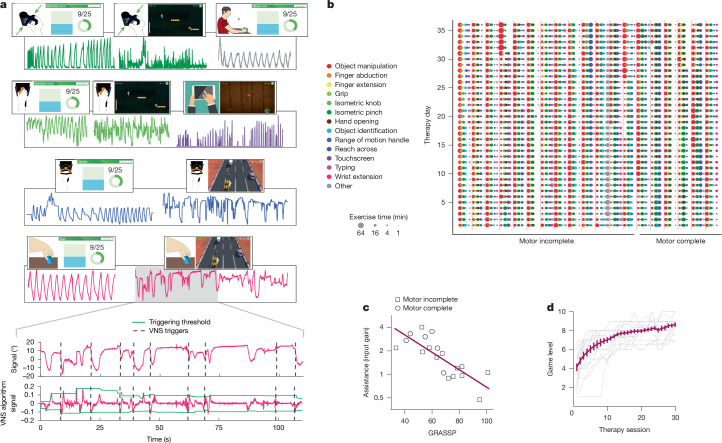
CLV therapy was intensive and individualized. **a**, Each CLV session was individualized and emphasized a variety of exercises. The rehabilitative regimen used a range of sensors, including a wireless accelerometer, strain gauge, rotary encoder, keyboard, video camera and touchscreen. An algorithm utilized the sensor data to select above-threshold movements during exercises and deliver real-time VNS. Panel **a** adapted from ref. ^28^, Sage (traffic racer and breakout); ref. ^35^, under a CC BY 4.0 license (finger module, and isotonic wrist pronation and supination ROM device); and from VectorStock (hand holding tablet). Colours correspond to the key in panel **b**. **b**, Over 36 days of therapy, participants completed approximately 4,800 activities, individualized to their level of impairment and residual hand and arm function. Participants performed 6–9 different activities per session, with each activity lasting an average of 3.3 min (IDR: 0.7–5.5 min) and typically repeated twice per session. The colour of the circle indicates the category of rehabilitative exercise performed during the activity, and the area of each circle reflects the number of minutes actively engaged in each task. We observed a 100% compliance rate, with all participants completing 36 sessions of therapy. A supplementary interactive HTML version of this figure provides comprehensive information on each session for each participant, including the number of VNS events, the number of repetitions, the assistance factor, the difficulty level, motor performance, and whether active or sham VNS was delivered. **c**, A therapist guiding exercises increased the linear assistance factor (gain), which was multiplied by the force or range of motion for each exercise for each person until they could succeed in gameplay. The average value was highly correlated with baseline GRASSP scores, which confirms that exercises were individualized and challenging. **d**, In addition, the overseeing therapist increased game level to ensure that tasks were challenging at every stage of therapy. Higher game levels required greater speed and precision. The average game level increased steadily over the 36 days of therapy for all participants (Pearson correlation, *P* < 0.001).

**Extended Data Fig. 2:**
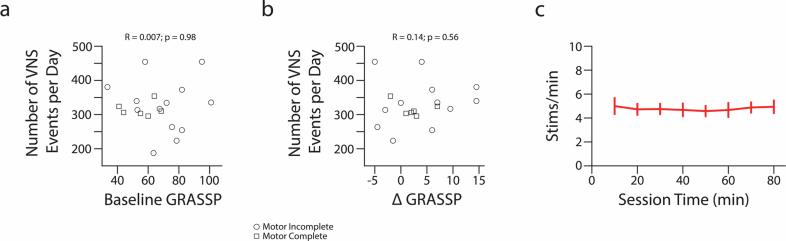
The number of stimulations delivered during therapy is stable across a range of impairments and over therapy sessions. The number of VNS events delivered during a rehabilitation sessions was not correlated with either baseline GRASSP score (a) or change in GRASSP score (b), indicating that the therapy was scaled by the level of impairment to ensure a consistent number of stimulations. Symbols denote individual participants. (c) Additionally, stimulation rate was stable across the duration of rehabilitative sessions, indicating that the algorithm was robust against effects of fatigue or other sources of variability in performance. Data are presented as mean ± SD number of VNS events per 10-minute bin over the course of each rehabilitation session for each participant.

Although some degree of finger and wrist weakness is ubiquitous in this patient population, there is a great deal of individual heterogeneity in the nature and magnitude of functional impairments. As expected, all participants in this study presented with a unique pattern of impairment, illustrated by variable deficits in GRASSP category scores (Fig. 2d). To ensure that each participant received exercises tailored to their specific impairment, the rehabilitative training regimen for each participant consisted of a personalized set of six to nine exercises and accompanying games (Fig. 3a,b, Supplementary Video 1 and ‘Individualized therapy sessions’ in Supplementary Information). Exercises were further individualized by adjusting the degree to which the force, torque or range of motion produced on each exercise was multiplied by a linear assistance factor (that is, gain) to make the games challenging but playable. The average assistance factor applied to each participant during rehabilitation was well correlated with their GRASSP score (Pearson correlation, *R*^2^ = 0.63, *P* = 5 × 10^−5^; Fig. 3c). Therapists observed exercises and, when possible, progressively increased task difficulty by gradually reducing the assistance factor. Therapists also adjusted task difficulty by increasing the level of each game, which necessitated greater speed and precision (Fig. 3d). Over the course of the study, exercise difficulty was adjusted 154 ± 15 times per person to ensure that participants were maximally challenged and at the limits of their ability, as documented by a stable rate of errors during gameplay. The observations that game error rate (1.1 ± 0.3 errors per minute, *R*^2^ = 0.00, *P* = 0.9), time actively engaged with task training (38.6 ± 0.7 h, *R*^2^ = 0.03, *P* = 0.5) and number of exercise repetitions per day (1,537 ± 74 repetitions, *R*^2^ = 0.002, *P* = 0.8) were not correlated with GRASSP confirm that the therapy parameters were set to be highly challenging despite a wide range in impairment nature and severity. Stimulation triggering was governed by an algorithm that scaled adaptively based on performance measured with the sensorized controllers^30^, which resulted in stable delivery of VNS across a wide range of impairments and over the course of rehabilitation sessions (Extended Data Fig. 2).

## CLV enhances arm and hand function

**Fig. 4:**
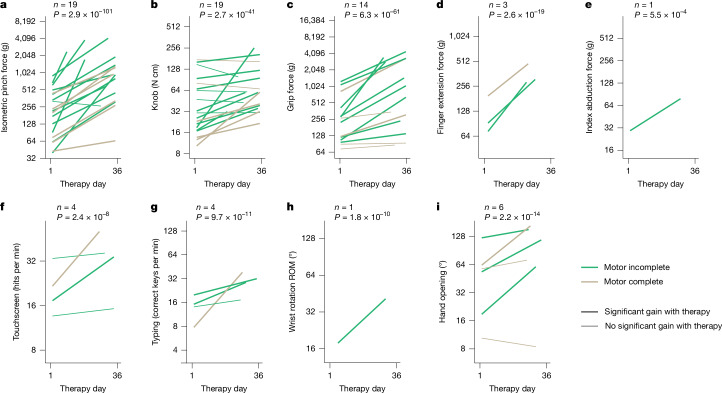
CLV improves hand and wrist strength, speed and range of motion. **a**,**b**, Pinch force (**a**) and knob torque (**b**), exercises performed by all participants, steadily increased over the course of therapy in the substantial majority of individuals. **c**–**i**, Similarly, strength, speed and range of motion (ROM) increased across a wide range of isometric and dynamic exercises. A linear mixed model was fitted to the data for each task, which was collected from daily rehabilitative training sessions. There was a significant fixed effect of therapy day for each task, as noted by the *P* value on each of the figure’s panels. *n* denotes the number of participants that performed the task as part of their individualized therapy. The thick lines indicate participants who made statistically significant increases in task performance as a function of therapy day. The *y* axis maximum represents the median of unimpaired controls. Note that because exercise regimens were individualized, not all participants performed all exercises.

**Extended Data Fig. 3:**
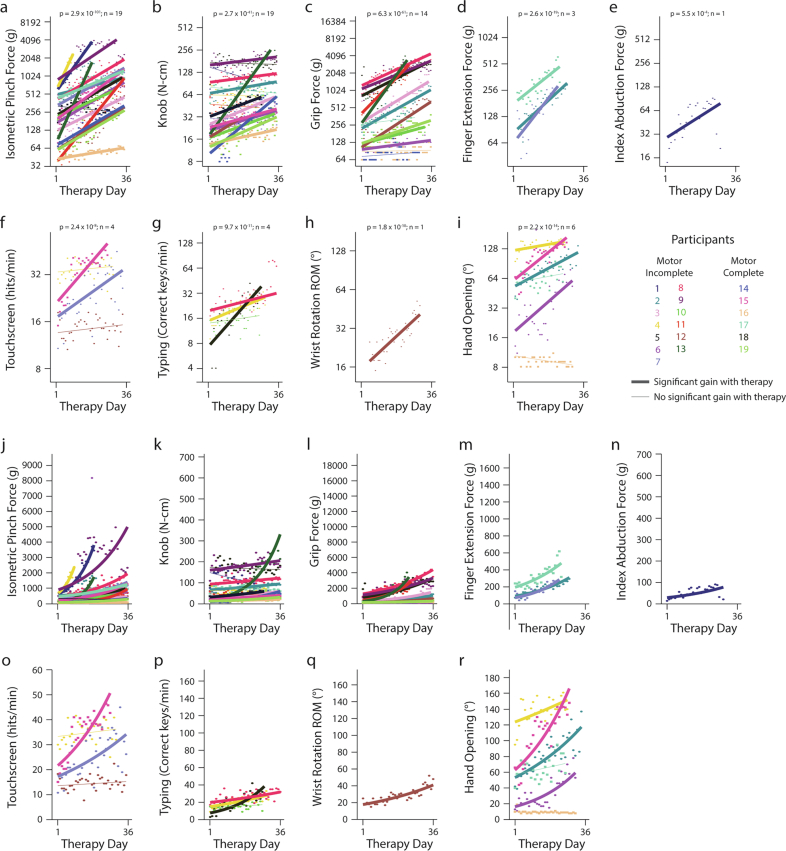
CLV improves hand and wrist strength, speed, and range of motion. a) Pinch force and b) knob torque, exercises performed by all participants, steadily increased over the course of therapy in the substantial majority of individuals. c-i) Similarly, strength, speed, and range of motion increased across a wide range of isometric and dynamic exercises. A linear mixed model was fitted to the data for each task. There was a significant fixed effect of therapy day for each task, as noted by the p-value on each of the figure’s panels. n denotes the number of participants that performed the task as part of their individualized therapy. Performance metrics for each day are shown as dots. Thick lines indicate participants who made statistically significant increases in task performance as a function of therapy day. The y-axis maximum represents the median of unimpaired controls. (j-r) Same as above, but plotted on a linear scale.

Isometric pinch and knob exercises were included in the therapy programme developed for every person, as distal hand function was consistently impaired (Fig. 2d). Consequently, we used these metrics to track progress in all participants and assessed whether a course of CLV would produce improvements in these commonly impaired measures. Thirty-six sessions of therapy produced statistically significant increases in pinch force for 18 out of 19 people. The average pinch force increased by 936 ± 247 g, a gain of 393 ± 102% over baseline (two-tailed, paired Student’s *t*-test, *n* = 19, *P* = 5 × 10^−8^; Fig. 4a and Supplementary Video 2). Similarly, the wrist torque that participants could exert on a door knob increased significantly in 13 out of 19 people, with an average increase of 28.5 ± 16.8 N cm, representing a 152 ± 87% improvement (two-tailed, paired Student’s *t*-test, *n* = 19, *P* = 0.007; Fig. 4b and Supplementary Video 3). Over the course of therapy, the force, speed and range of motion produced during various different exercises were approximately doubled (Fig. 4c–i and Extended Data Fig. 3). These gains exceed the preregistered outcomes. As expected from earlier studies in chronic cervical SCI, some participants failed to make statistically significant gains on some exercises^36^. These observations suggest that CLV can significantly improve hand strength in people with chronic cervical SCI, although rigorously validated methods are necessary to ensure that changes in force production are clinically meaningful.

## CLV improves clinical outcomes

**Fig. 5:**
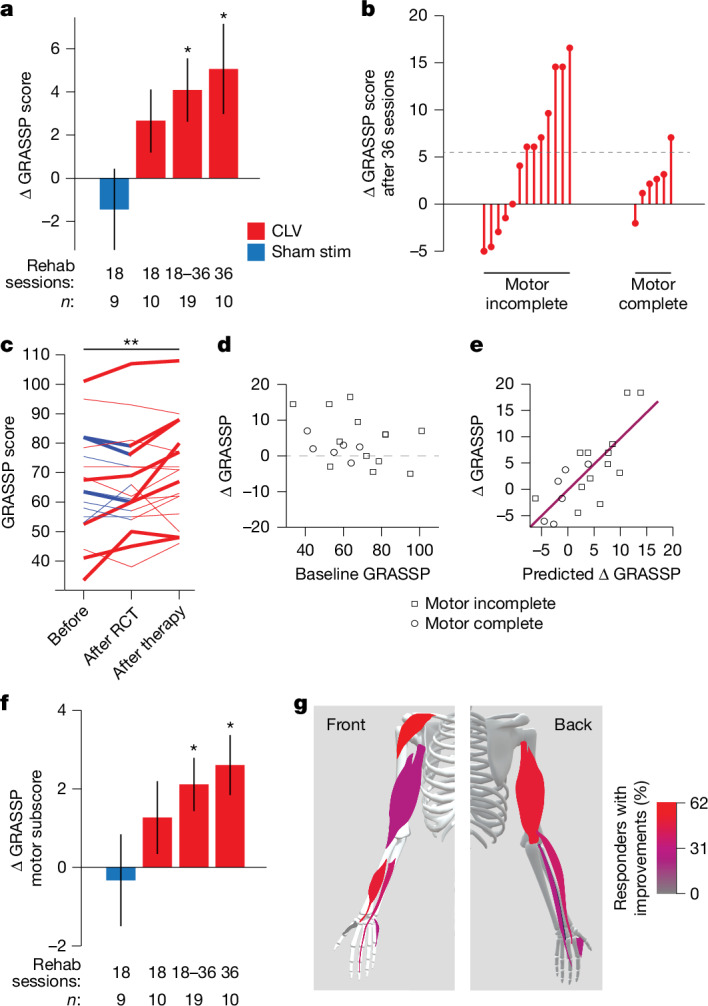
CLV improves clinical metrics of upper limb function. **a**, In the double-blinded, sham-controlled phase of the study, GRASSP score was significantly improved compared with baseline in all participants after 18 sessions of CLV and in the subset of participants that received 36 sessions of CLV. No gains were observed in the group that received intensive rehabilitation with sham stimulation (stim). CLV produces a medium effect in improvement in upper limb function (Cohen’s *d* effect size > 0.5). **b**, After the completion of 36 sessions of therapy (after therapy), 8 individuals made meaningful increases in the GRASSP score. This effect appears to be larger in individuals with motor incomplete injuries (AIS C and D). Dashed line indicates the cut-off for a meaningful increase, as defined by a 6-point or greater increase in GRASSP score. **c**, Meaningful improvements (denoted with bold lines) were observed across participants with severe, moderate and mild impairments in hand and arm function. **d**, Single characteristics, including the baseline GRASSP score, were not correlated with treatment response. Dashed line denotes no change in GRASSP score. **e**, However, a multiple linear regression model (detailed in Extended Data Table 3) with the AIS grade, GRASSP strength subscore, and GRASSP palmar and dorsal sensory subscores as inputs was highly correlated with the change in the GRASSP score from baseline to completion of 18 sessions of CLV. **f**, The strength subscore of GRASSP was significantly improved compared with baseline with CLV and unchanged with equivalent rehabilitation with sham stimulation. **g**, A range of the ten muscle groups evaluated during the GRASSP assessment improved with therapy. Muscles are colour coded to illustrate the percent of responders who exhibited measurable improvements in each muscle. Deltoid, pollicis longus, elbow extensors and wrist extensors were among the most commonly improved muscles. Panel **g** adapted using Sketchfab under a CC BY 4.0 license. Panels **a**,**c**,**f** used two-way paired Student’s *t*-test versus baseline for CLV, and Wilcoxon signed-rank versus baseline for sham stimulation; *P < 0.05 and **P < 0.01. Group data are presented as mean ± s.e.m.

**Extended Data Fig. 4:**
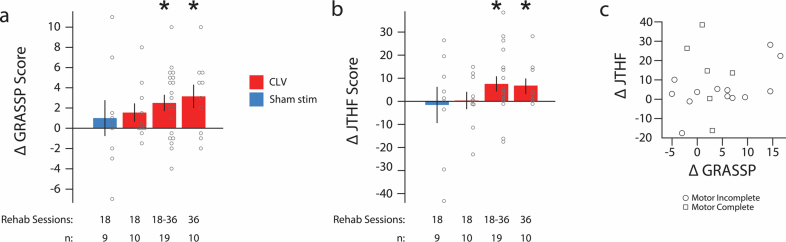
CLV produces modest gains in GRASSP score in the untrained arm and on functional measures. (a) GRASSP score in the untrained arm was significantly improved compared to baseline in all participants after 18 sessions of CLV and in the subset of participants that received 36 sessions of CLV. No gains were observed in the group that received intensive rehabilitation with sham stimulation. Gains in the untrained arm were more modest than those observed in the trained arm. Improvement in the untrained arm was not expected because VNS-induced plasticity is specific to the paired training. However, these improvements may be explained by some amount of bilateral involvement in training, generalization, or VNS-dependent pruning of synaptic connectivity in hyperconnected networks, each of which could reduce spasticity or otherwise produce benefits to the untrained arm. Future physiological studies are needed to understand why small, but statistically significant, improvement was observed in the untrained arm. (b) Additionally, participants that receive 36 sessions of CLV exhibit improved Jebsen-Taylor Hand Function scores in the trained arm compared to baseline. (c) Though not significant in the whole population, an exploratory analysis of only motor incomplete participants demonstrates a significant correlation between change in GRASSP score and JTHF score at the end of therapy (R = 0.59; p = 0.034), potentially indicative of clinical and functional gains. Symbols (and n) indicate individual participants. For panels a and b: Paired Student’s t-test v. baseline, * indicates p < 0.05. Group data are presented as mean +/− SEM.

We next evaluated whether these gains in hand and arm capabilities translated to improvements in clinical metrics. Collectively, we observed a statistically significant improvement in GRASSP score after CLV (two-tailed, paired Student’s *t*-test, *n* = 19, 4.1 ± 1.5 points, *P* = 0.01; Fig. 5a). This improvement exceeds the preregistered outcome and casts doubt on the long-held notion that additional gains are not possible in people with traumatic SCI more than 1 year post-injury and highlights the potential of CLV as a novel therapy for SCI^37–41^. Thirty-six sessions of CLV produced accumulating gains approximately double that observed after 18 sessions (Fig. 5a). In addition, the cumulative gains are consistent with the hypothesized mechanism of action by which real-time VNS promotes synaptic plasticity in spared networks^25^. We also observed modest gains in the untrained arm (Extended Data Fig. 4a).

The magnitude of recovery appeared to be dependent on the completeness of the injury. The majority of people with motor incomplete injuries (7 of 13, AIS C or D) exhibited a meaningful response to therapy, as defined by a six-point or greater increase in GRASSP score (Fig. 5b). By contrast, only one of the six people with motor complete injuries (AIS B) made a meaningful improvement. The greater recovery observed in individuals with motor incomplete injuries may suggest that individuals with motor complete injuries do not benefit from CLV. Alternatively, the observation that this population exhibited half the benefit seen in those with motor incomplete injuries benefit (2.3 ± 1.3 versus 4.9 ± 2.1 points) could be explained if this population requires twice as much therapy to produce the same benefit. Future studies are needed to distinguish between these possibilities.

In addition to GRASSP scores, we performed an exploratory analysis to evaluate whether CLV would enhance performance of common activities of daily living, as measured with the Jebsen–Taylor hand function test. At the conclusion of CLV, the Jebsen–Taylor hand function score increased by 7.5 ± 3.1 points (two-tailed, paired Student’s *t*-test, *n* = 19, *P* = 0.03) compared with baseline (Extended Data Fig. 4b). An exploratory analysis revealed that the Spinal Cord Independence Measure Version 3 (SCIM-III) components that emphasize arm and hand use demonstrated significant improvement after CLV (Wilcoxon signed-rank test, +1.3 ± 0.7 points, *n* = 19, *P* = 0.04). Collectively, these results demonstrate that hand and arm function can be improved in people with chronic, incomplete cervical SCI.

## Predictors of treatment response

Given the heterogeneity along a range of participant characteristics, we sought to determine whether there were potential relationships between these baseline features and treatment response. Single characteristics, including baseline impairment severity (Fig. 5c,d; Pearson correlation, *R*^2^ = 0.1, *P* = 0.2), time since injury (*R*^2^ = 0.005, *P* = 0.8) and age (*R*^2^ = 5 × 10^−4^, *P* = 0.9), were not associated with treatment response. This observation is consistent with findings in stroke^42^ and suggests that this approach could be potentially valuable for a large number of people living with chronic, incomplete SCI. Multiple linear regression modelling indicates that treatment response is greatest in individuals classified as motor incomplete and that have less strength and poor palmar sensation with preserved dorsal sensation (Fig. 5e and Extended Data Table 3; *R*^2^ = 0.6, *P* = 0.009). This aligns with preclinical evidence showing that both lesion characteristics and pathological synaptic plasticity directed to non-functional skin surfaces shape the potential to benefit from CLV^21^. These initial findings highlight the importance of exploring predictors of response in a larger study.

## Double-blinded RCT phase

To estimate the effect size that VNS contributed to recovery, participants were randomized to receive either sham stimulation (*n* = 9) or active CLV (*n* = 10) during the first 18 sessions of high-intensity physical therapy (Extended Data Fig. 1; additional description of trial design, clinical protocol and consent procedures are available in Supplementary Information). Because all participants were implanted with the miniaturized device, received comparable high-intensity rehabilitation and stimulation was software controlled, therapists, assessors and participants were blinded to group assignment. Participant blinding was confirmed by questionnaires at the end of the randomized controlled trial (RCT) phase, which revealed that 58% of participants (11 of 19) were incorrect or uncertain about whether they received active VNS or sham stimulation, comparable with previous studies that used a similar blinding strategy^43^.

The RCT phase of the study confirmed that adding VNS to rehabilitation provides a medium-sized effect size over equivalent rehabilitation with sham stimulation, although this study was not powered to reach statistical significance (Fig. 5a; Cohen’s *d* = 0.58, *P* = 0.3). The effect size observed in this study was comparable with that observed in people with ischaemic stroke, which is now an FDA-approved therapy^43^. These findings highlight the need for a prospectively powered study to evaluate the efficacy of CLV in individuals with chronic SCI. On average, the eight responders lost 23 ± 4% of their disability as measured by GRASSP. Future studies are needed to determine whether greater gains are possible with additional therapy sessions and whether some proportion of the non-responders can be converted to responders by adjusting VNS intensity. Although animal studies have consistently shown that the treatment effects of VNS during therapy are an inverted-U function of stimulation intensity^44–47^, this study did not seek to individualize VNS current.

To clarify the degree to which the current set of rehabilitative tools and exercises was able to improve specific muscle groups, we evaluated the probability that each of the ten muscles tested in the GRASSP assessment were improved in responders (Fig. 5f). Improvements were observed in proximal muscles of the arm and intrinsic and extrinsic muscles of the hand (Fig. 5g). The only muscle that did not improve was the opponens pollicis, which opposes the thumb. Additional work is needed to ensure that rehabilitative exercises are developed to target this muscle, as well as additional muscles that are not included in the GRASSP assessment but that may benefit from targeted exercises.

## Automated CLV is safe and effective

**Extended Data Fig. 5:**
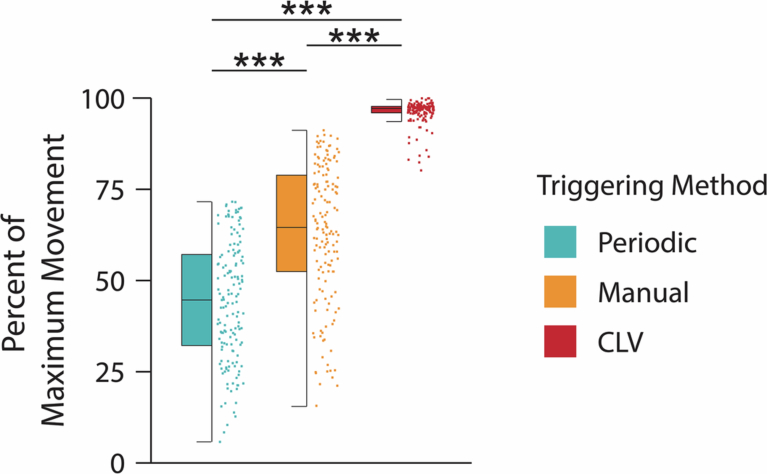
VNS was delivered on above average movements for each participant. Closed-loop triggering produces bursts of VNS that coincide with the largest movements that occurred during therapy. Both therapist and an automated algorithm were able to outperform open-loop (e.g. periodic) triggering. Automated closed-loop triggering using real-time sensor data was able to outperform therapists using visual inspection to determine stimulation timing. Symbols denote individual exercises for participants that received both manual and automated triggering, and boxes show median and interquartile range. Unpaired Student’s t-test across conditions, *** indicates p < 1 × 10^−17^.

The core premise of CLV is the coupling of task-specific training and real-time neuromodulation. On average, during sessions in which active VNS was paired with rehabilitative training, participants received 341 ± 15 half-second stimulation bursts to coincide with selected movements. Participants in the active group received 11,793 ± 990 total stimulations over the course of therapy. We utilized two strategies to govern the triggering of stimulation during movement. Initially, for the first 96,000 stimulation events, triggering decisions were made by therapists observing rehabilitative exercises using a dedicated software application. Manual triggering resulted in VNS events coinciding with movements that were twice the average amplitude above a non-targeted periodic triggering scheme, confirming that the therapists successfully coupled stimulation with task-focused movements (Extended Data Fig. 5; unpaired, two-tailed Student’s *t*-test, *P* = 2 × 10^−74^). Using the therapists triggering scheme as a ground truth, we developed and validated an automated algorithm to monitor movement and trigger stimulation^30^. As expected, the automated triggering method significantly outperformed therapists (unpaired, two-tailed Student’s *t*-test, *P* = 2 × 10^−34^). The algorithm governed stimulation for the remaining 137,000 stimulation events during the study, which allowed the therapists to focus solely on guiding rehabilitative exercises. Collectively, this shows that an automated approach to CLV is feasible to deliver.

In conjunction with feasibility, a key consideration in the delivery of any therapy with an implanted device is safety. After 19 implant surgeries, 760 patient visits, 3.7 million total VNS pulses and a collective 42 patient-years of device contact, there were zero serious device-related adverse events and zero unexpected device-related adverse events. All devices performed to specification, and we observed no device complications or technical failures. These findings are consistent with the larger corpus of literature using standard VNS strategies and reinforce that this approach is safe and well tolerated.

Because the CLV strategy tested in this study used a novel miniaturized VNS system, we assessed occurrence of adverse events. All study-related adverse events were classified as mild (Extended Data Table 4). As expected, the rate of post-surgical pain at the incision site was comparable with published rates using VNS in individuals with stroke^48^. By contrast, rates of voice alteration appear to have been reduced in this study. This probably reflects the reduced device size and simplified surgical procedure that obviates subcutaneous tunnelling in the neck, and the dramatically lower total charge delivery required for CLV relative to conventional VNS for epilepsy.

**Extended Data Fig. 6:**
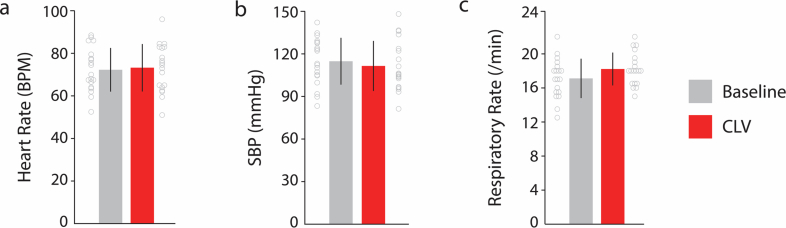
CLV does not affect cardiovascular function in individuals with SCI. To determine whether CLV would impact measures of cardiovascular function, we measured vital signs prior to the initiation of therapy (baseline) and after the completion of the first 18 sessions of CLV (n = 19 participants). Heart rate (a), systolic blood pressure (b), and respiratory rate (c) were not influenced by CLV. Data are presented as mean ± STD. Symbols denote individual participants.

The use of CLV raises the potential for a specific safety concern in the context of SCI. Because the vagus nerve exerts control of autonomic function, it is conceivable that VNS could influence cardiovascular function and induce autonomic dysreflexia in individuals with SCI. Over the course of treatment, we observed no changes in heart rate or blood pressure between active and sham stimulation (Extended Data Fig. 6). In addition, no instances of autonomic dysreflexia were observed with stimulation. Consistent with results in animal models, these findings indicate that the stimulation parameters used for CLV do not meaningfully alter autonomic function and reinforce the safety of this strategy in individuals with SCI^49^.

## Discussion

Here we report the first-in-human use of CLV in 19 individuals with chronic, incomplete cervical SCI. Delivery of CLV using a novel, miniaturized VNS system was safe and feasible, with individuals receiving approximately 12,000 stimulations combined with weeks of intensive, personalized, task-focused rehabilitation. The majority of individuals exhibited meaningful improvements in multiple metrics of arm and hand function, and a longer course of therapy produced accumulating gains. The magnitude of upper limb recovery and proportion of responders was comparable with that observed using a similar approach in chronic stroke, which recently received the first-ever FDA approval for an intervention to improve recovery in the chronic phase. Collectively, these findings argue against the long-held belief that recovery after SCI is limited in the chronic phase and highlight the clinical potential of combinatorial treatment approaches.

Some limitations of the present study merit consideration and should be addressed in future trials. Because there are no validated biomarkers of VNS, we did not collect direct evidence of neuromodulator engagement; such a metric, as well as direct measures of plasticity, could potentially be used to individualize stimulation parameters in future implementations. Personalization of neuromodulator release or plasticity represents a potential means to increase the proportion of individuals that exhibit a clinically meaningful response, a key consideration for an implanted device. Given the limited size of this first-in-human study, subsequent trials should evaluate a larger pool of participants to examine potential predictors of recovery, including demographic characteristics or the GRASSP predictor developed here, and expand evaluation of hand and arm metrics at longer times post-therapy. In summary, this study provides strong evidence that CLV is safe, feasible to deliver and provides initial evidence of robust improvements of arm and hand function that supports investigation in a larger trial.

## Methods

### Overall study procedure

The trial was designed as a double-blinded, randomized, sham-controlled early feasibility study (Extended Data Fig. 1). The clinical protocol and informed consent document are included in the Supplementary Information. Individuals who indicated interest in participation and met general criteria in an initial screen underwent informed consent. After enrolment, all participants underwent implantation of the miniaturized VNS device. Approximately 4 weeks after implantation, all participants received a post-implantation baseline assessment. Participants then received 18 sessions of rehabilitation with active CLV or sham stimulation in accordance with their randomization. After the initial 18 sessions, participants received an additional 18 session of rehabilitation with active VNS, regardless of their previous group assignment. The primary objectives evaluated safety, feasibility and changes in measures of arm and hand function. Outcomes were assessed after enrolment, after implantation, after completion of 18 sessions of therapy and after completion of 36 sessions of therapy. Occurrence of adverse events was screened throughout the course of the study.

### Regulatory compliance and ethics

The use of the miniaturized VNS device used in this study received investigational device exemption (approval from the FDA investigational device exemption approval ID G190032). In addition, all study procedures were approved by the Baylor Scott and White Research Institute Institutional Review Board, the University of Texas at Dallas Institutional Review Board, and the US Department of Defense Human Research Protections Office. The study was conducted in compliance with all relevant regulatory and ethical guidelines. The trial was preregistered at ClinicalTrials.gov (NCT04288245).

### Participants

Participants were recruited from 5 March 2021 to 30 June 2023. Trial recruitment ended when budgeted funds for the trial were expended. Patient characteristics are delineated in Extended Data Table 1. A total of 293 potential participants contacted us or were identified through an established referral network at Baylor Scott and White Spinal Cord Injury Model System, study flyers, local advertisements and online advertisement. Of these, 19 met criteria, elected to participate, were consented and enrolled, and ultimately underwent implantation. Informed consent was obtained in a private setting after consultation with study staff to answer any questions. The informed consent process followed our established procedure, which is included in the Supplementary Information.

Participants met the following key inclusion criteria: (1) first time cervical SCI occurring at least 12 months before and resulting in AIS grade B, C or D (confirmed during the baseline visit by an experienced clinician); (2) residual movement in the upper limb and hand in either arm; (3) appropriate candidate for VNS implantation; (4) between 18 and 64 years of age; (5) a signed and dated informed consent form; and (6) willing to comply with all study procedures and were available for the duration of the study. Participants were excluded based on the following criteria: (1) SCIs by sharp objects, firearms, and non-traumatic or congenital causes; (2) evidence of recurrent laryngeal nerve injury; (3) excessive scar tissue in the neck; (4) concomitant clinically significant brain injuries; (5) previous injury to the vagus nerve; (6) previous or current treatment with VNS; (7) receiving any therapy that would interfere with VNS; (8) pregnant or lactating; (9) psychiatric disorders, psychosocial and/or cognitive impairment that would interfere with study participation; (10) abusive use of alcohol and/or illegal substances; (11) participation in other interventional clinical trials; (12) known immunodeficiency or receipt of chronic corticosteroids, immunosuppressants, immunostimulating agents or radiation therapy within 6 months; (13) significant comorbidities or conditions associated with high risk for surgical or anaesthetic survival; (14) active neoplastic disease; (15) significant local circulatory problems; (16) any medical condition or other circumstances that might interfere with their ability to receive rehabilitation or return for follow-up visits; (17) any condition that would preclude adequate evaluation of the safety and performance of the device; (18) aphasia and other cognitive deficits that interfere with provision of informed consent; (19) recent history of syncope; (20) recent history of dysphagia; (21) currently require, or are likely to require, diathermy; (22) significant respiratory issues that would interfere with participation; (23) non-English speaking; (24) acutely suicidal and/or have been admitted for a suicide attempt; and (25) incarceration or legal detention. Full eligibility criteria can be found on ClinicalTrials.gov (NCT04288245). No changes were made to eligibility criteria after trial commencement.

### VNS device implantation

All 19 enrolled participants were implanted with a next-generation VNS device^29^. This device is over 50 times smaller than conventional VNS systems, largely due to the offloading of the battery and elimination of the need for leads. Both of these changes simplify surgical implantation by necessitating only a single incision at the neck and obviating the need for tunnelling. Implantation was performed by surgeons with experience in the implantation of VNS systems. After surgical preparation and induction of general anaesthesia, the skin and platysma overlying the left cervical vagus nerve were incised. The sternocleidomastoid muscle was mobilized laterally to reveal the carotid sheath, and the vagus nerve was dissected free circumferentially over a length of 3–4 cm. The vagus nerve was placed inside the silicone cuff, which positions the electrodes of the implanted pulse generator (IPG) in contact with the nerve. The silicone cuff was closed around the nerve with two 4-0 permanent sutures. The IPG was then positioned superficially to facilitate alignment with the external components during stimulation. Before closure of the skin, wireless communication and power functions of the IPG were verified. After confirmation of device functionality, closure of the platysma and skin with absorbable sutures was performed, followed by a second communication verification. Impedance checks confirmed that all participants were within the acceptable range. Average surgical time was 38 ± 2 min. Routine post-anaesthetic care was provided. Approximately 1 week later, participants returned to the clinic for a follow-up visit to assess recovery.

### VNS delivery

Participants were randomized 1:1 to receive either 18 sessions of rehabilitation with sham stimulation followed by 18 sessions of rehabilitation with active VNS (*n* = 9) or 36 sessions of rehabilitation with active VNS (*n* = 10). A blocked software-randomized design was used with a block size of two. The blocking covariate was impairment severity (treated arm GRASSP ≤ 58 versus GRASSP > 58). Ten participants were randomized to receive active (0.8 mA, 30 Hz for 500 ms) VNS for all 36 sessions of VNS. Nine participants were randomized to receive sham VNS for the first 18 sessions and active VNS for the second 18 sessions (Extended Data Fig. 1).

Before the beginning of each rehabilitation session, vital signs were collected with a digital blood pressure cuff. During rehabilitation sessions, the external power and communication module (PCM) was placed in a band around the neck of the participant with the coil aligned over the IPG. Because stimulation was only delivered during rehabilitative training sessions, the PCM was only worn during these sessions. Each 0.5-s train of VNS was delivered concurrent with exercises during rehabilitative training (as detailed below), and comprised 0.8-mA 100-µs biphasic pulses at 30 Hz, as in previous studies.

The concurrent timing of VNS and movement is based on the principle that precisely timed neuromodulatory feedback can direct specific synaptic changes to enhance recovery. After an SCI, a subset of neurons within the central nervous system is affected by the injury, and promoting long-term potentiation and depression within these networks has long been recognized as an approach to support recovery^1,50,51^. The ability to direct synapse-specific, adaptive plasticity within the affected networks while not influencing other networks is at the core of the credit assignment problem, which articulates the challenge of identifying which synapses should be changed to improve motor function^17,52–54^. CLV leverages the synaptic eligibility trace, a phenomenon in which the arrival of neuromodulatory reinforcement within seconds promotes plasticity in recently active networks and not in inactive networks^16,55–57^, to direct changes within specific synapses. In CLV, motor networks controlling the upper limb affected by the SCI are engaged by rehabilitative exercises, and closed-loop VNS delivered in response to a target movement triggers neuromodulatory release to produce therapeutic synaptic plasticity within these networks^3,11^.

All participants underwent implantation, a key feature of blinding. In addition, all participants received equivalent individualized rehabilitation regimens and donned the external device during rehabilitation sessions, regardless of active or sham group assignment. The stimulation parameters were software controlled and were preset by a study staff member who was not involved in rehabilitation, data collection or analysis, allowing therapists and assessors to maintain blinding. Most participants did not perceive the stimulation or only felt the first few stimulations each day and rapidly adapted to the sensation. To maintain blinding, at each rehabilitation session, participants in the sham group received active stimulations of reducing strength beginning at 0.8 mA and decreasing with each trigger to 0.6 mA, then 0.4 mA, then 0.2 mA, then 0.1 mA and then 0 mA for the remainder of the session. Preclinical and clinical studies indicated that this amount of stimulation was insufficient for therapeutic effect and sufficient to ensure blinding. Participants were instructed that they may initially perceive stimulation, but the perception may fade. Participant blinding was confirmed with a questionnaire at the end of phase 1 of the study, in which 11 of 19 participants were incorrect or uncertain about group assignment. Although therapists and assessors were blinded, blinding status was not directly surveyed in these individuals and should be assessed in future studies to confirm effective concealment.

### Personalized rehabilitation regimen

All 19 participants completed 36 sessions of intensive rehabilitation at a rate of approximately 3 per week. Each session was approximately 90 min long. Both groups followed the same visit schedule and received equivalent rehabilitation of one arm. Clinical judgement was used to select the arm that was most likely to benefit from therapy.

Personalized exercise regimens were selected based on the ability profile of each participant. Each regimen incorporated conventional rehabilitative exercises and training on a computer-based rehabilitation system^28^. As appropriate for their level of ability and expressed interest, participants also performed functional tasks (labelled as object manipulation in Fig. 2 and ‘Individualized therapy sessions’ in Supplementary Information), including jar opening, threading a nut on a bolt, lifting cans, writing, and inserting and turning keys. Participants generally cycled between relatively short (1–5 min) sets of each exercise to mitigate substantial fatigue. Difficulty was continuously monitored by licensed therapists to ensure that the therapeutic exercises were challenging. If performance improved, difficulty was increased by reducing the linear assistance factor (that is, gain) and increasing the game level of the computer-based rehabilitation system, which had the effect of increasing the required movement speed, accuracy and force^28^. Assistance factor adjustments to the exercise regimens were made systematically, such that a typical progression for a single exercise included decreasing the input multiplier from the controller by 20%. In addition, progression involved increasing the number of repetitions or extending the duration of the exercise.

The closed-loop triggering scheme to deliver VNS concurrent with movements used two strategies. In the first strategy, the therapist overseeing the rehabilitation session used a button press in a software app to trigger stimulation on movements identified as above-average attempts based on specific performance metrics, such as increased force, speed, accuracy or fluidity of motion. This is the stimulation approach that has been applied in conventional studies using paired VNS therapy^43,58,59^. In the second approach, triggering was automatically controlled by a software algorithm, as previously described^30^. This strategy used real-time movement signals collected from the sensors in the rehabilitative training devices and delivered stimulation on movements that exceeded a continuously updated threshold. The relevant aspect of the movement for triggering was dependent on the gameplay; for example, gameplay controlled by pinch force used the force signal to determine triggering, whereas gameplay controlled by wrist rotation used degree of rotation to determine triggering^28,35^. The algorithm initiated stimulation within 500 ms when movement exceeded the 95th percentile of previous repetitions. The algorithm produced stable triggering across a range of impairments and over the course of rehabilitative sessions (Extended Data Fig. 2). Most participants received a combination of both stimulation-triggering strategies.

Individuals with lived SCI experience, including study participants, were iteratively involved in the design of the CLV system and therapy, including the development and implementation of the PCM and neckband, the rehabilitation devices and the design of the rehabilitative regimens. The components were designed to emphasize adoption among individuals with SCI, maximize comfort and promote engagement with the therapy. Consistent with this, study participants indicated a high level of satisfaction with the therapy (4.6 ± 0.2 on a 5-point Likert scale surveyed at the end of the study), a critical consideration for eventual clinical adoption.

### Outcome assessments

#### GRASSP

The GRASSP (version 1) is a clinician-administered assessment quantifying three domains describing upper limb function and impairment^60^. This assessment was specifically designed to evaluate the effect of novel interventions on upper limb impairment in the traumatic tetraplegic population. The preregistered clinical end point was a greater than four-point increase in GRASSP. An improvement of six points or more in the trained limb was considered a meaningful difference. Higher scores are associated with improved arm and hand function. GRASSP scores are categorical and were collected at each assessment session. Percent disability, as reported in the main text, was derived from GRASSP scores. This metric was calculated as the number of GRASSP points gained during therapy as a proportion of the difference between baseline pre-therapy score and the total number of available points to reach a maximum score.

#### Quantitative force and range of motion assessment

A suite of rehabilitative tools was used to assess force and range of motion in the hand and wrist throughout the course of the study^28,35^. The system includes modules to quantify wrist rotational range of motion and torque on a doorknob and a revolving D-handle, wrist flexion–extension range of motion and torque, and finger flexion–extension force. These continuous values were collected at each rehabilitation session, as appropriate for the assigned exercises for each participant. The preregistered strength end points were greater than 10% increases in finger pinch and flexion force following active VNS, 10% increases in wrist flexion and extension force following active VNS, and 10% increases in wrist pronation and supination force following active VNS.

#### Jebsen–Taylor Hand Function Test

The Jebsen Taylor Hand Function Test is a widely used standardized and objective measure of fine and gross motor hand functions that uses simulated activities of daily living^61^. This was an exploratory end point.

#### Spinal cord independence measure

As this study only performed hand and arm rehabilitation, we did not expect to see improvements in respiration, lower body function, bowel function, bladder function or mobility. We thus excluded these metrics from SCIM-III to produce an 18-point measure of independence associated with arm and hand function. This composite score includes feeding, grooming, toileting, upper body bathing and upper body dressing, all of which require arm and hand function. This was an exploratory end point.

#### American Spinal Injury Association Impairment Scale

The AIS is the gold-standard assessment to evaluate level and completeness of injury in individuals with SCI. In this study, AIS was evaluated by R.G.H., a clinician with extensive expertise in SCI, at baseline and at the end of each phase. AIS grade was used to confirm study eligibility.

### Data analysis

The primary and secondary study outcomes were reviewed by the regulatory bodies and were preregistered on ClinicalTrials.gov (NCT04288245). Data in the figures and text are presented as mean ± standard error of the mean unless otherwise indicated. Comparisons across groups were made using unpaired Student’s *t*-tests or Wilcoxon rank-sum tests. As appropriate, comparisons across time were made using paired Student’s *t*-tests or Wilcoxon signed-rank tests. Before conducting any parametric test, the assumption of normality was first checked using the Jarque–Bera goodness-of-fit test. For Fig. 4 and Extended Data Fig. 3, a linear mixed model was fitted to the data from each task, and statistics were performed using that model. Pearson correlation tests were used to determine the correlation between a number of elements and GRASSP score changes. Standardized effect size was calculated using Cohen’s *d*. As there was high test–retest reliability (Pearson correlation coefficient, *R* = 0.95, *P* < 0.00001) for the baseline assessments performed before and after implantation and no significant difference (*P* > 0.05), these two values were averaged to serve as the baseline assessment. All comparisons used an α = 0.05.

### Reporting summary

Further information on research design is available in the Nature Portfolio Reporting Summary linked to this article.

## Online content

Any methods, additional references, Nature Portfolio reporting summaries, source data, extended data, supplementary information, acknowledgements, peer review information; details of author contributions and competing interests; and statements of data and code availability are available at 10.1038/s41586-025-09028-5.

## Supplementary information

Supplemental Statistical Information Description of the statistical methods and outcomes from statistical testing regarding assumptions of normality and linear mixed modelling.

Reporting Summary

Supplementary Data (Dataset) **Raw Assessment Dataset**. Demographic data for each person, GRASSP subscores at 4 assessment time points, baseline forces, and forces recorded during sessions.

Supplementary Data (interactive file) Individualized Therapy Sessions.

Supplementary Video 1 Exercise type was customized for each participant based on their ability. Initial exercise selection was based on the GRASSP profile (Fig. 3d) of each participant. Exercises were refined and progressed by licensed physical therapists to ensure tasks were consistently challenging. Participants completed computer-based exercises that were progressed by increasing the speed and precision required for success and by reducing the assistance factor so that more force or range of motion was needed. Every participant completed exercises with isometric pinch and knob inputs because all study participants exhibited reduced pinch force and knob torque. Each participant also completed exercises with real-world objects to ensure natural tactile stimulation and support skills needed for activities of daily living. During reach across exercises, participants pressed pucks in different locations while holding objects that required specific grips (e.g. key or cylindrical) to improve range of motion and grip strength. Other exercises included typing, touchscreen use, and stereognosis.

Supplementary Video 2 *CLV improves pinch force*. The video shows maximum pinch force at the beginning of therapy and at the end of therapy for participant MI12. Positive values reflect closing (flexion) of the index finger and thumb. Negative values indicate opening (extension). Participants were given real-time feedback that was scaled to their maximum performance to enable them to see when they were producing greater than average forces. In this game (called repetition mode), the software counts the individual events and triggers VNS on the largest events. The video ends with an overlay of the forces produced before and after therapy.

Supplementary Video 3 CLV improves range of motion. The video shows maximum torque at the beginning of therapy and at the end of therapy for participant MI12. Positive values reflect pronation of the wrist. Negative values indicate supination of the wrist. The video ends with an overlay of the range of motion that was available before and after therapy.

## Figures and Tables

**Extended Data Table 1:**
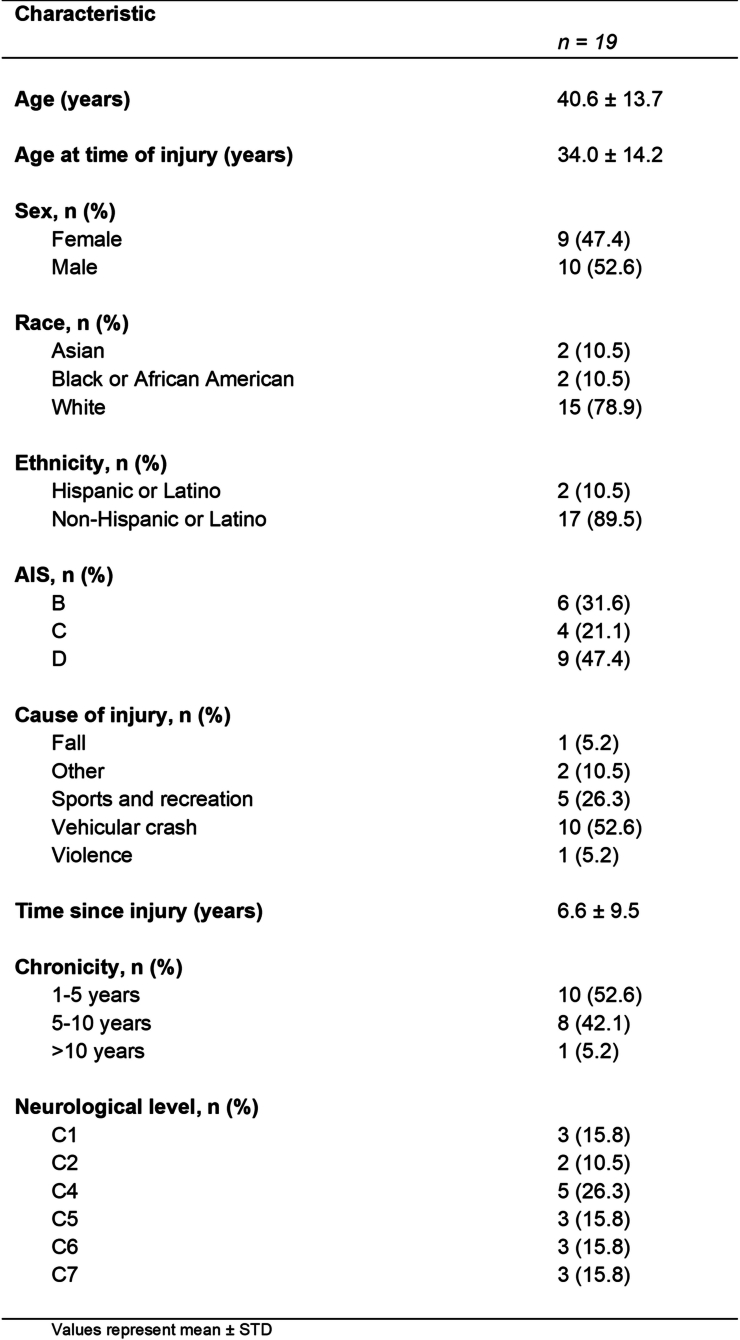
Participant Characteristics

**Extended Data Table 2:**
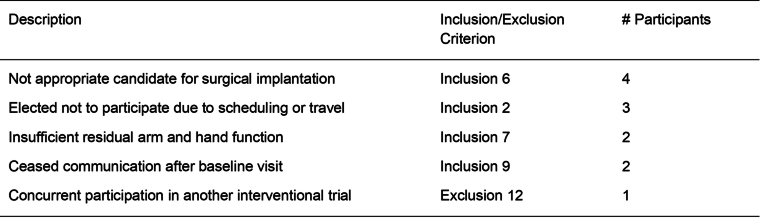
Reasons for Participant Discontinuation after Enrollment

This table provides information regarding the reasons that the twelve participants failed to meet criteria for study participation. The inclusion/exclusion criteria column indicates which pre-registered study criterion that the participant failed to meet, resulting in discontinuation prior to implantation.

**Extended Data Table 3:**
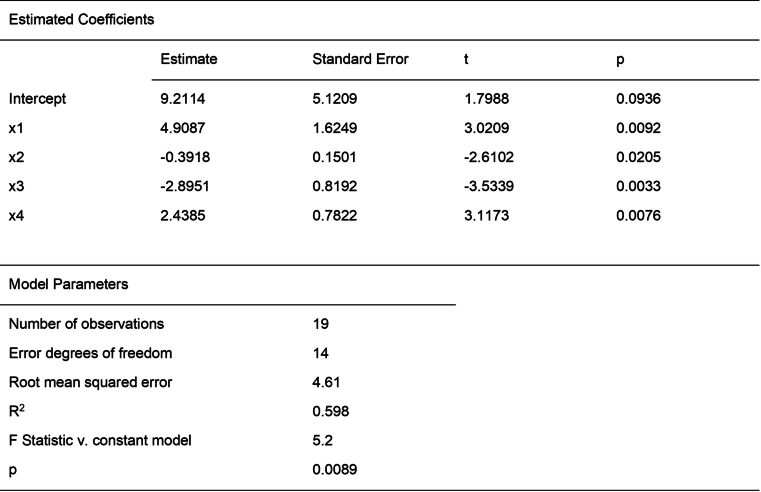
Predicted GRASSP Score

The model for predicting the change in GRASSP was derived from a linear regression model based on AIS and three of the GRASSP subtotals using MATLAB. Values for these and all other metrics for all participants can be found in the supplemental data file. The model parameters are reported below. Predicted GRASSP = (AIS x 4.9) + (Strength x -0.39) + (PalmSensation x -0.29) + (DorsalSensation x 0.24) + 9.2.

**Extended Data Table 4:**
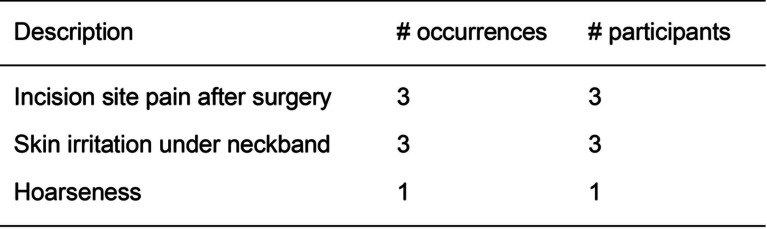
Study-Related Adverse Events

This table includes all AEs classified as probably or definitely related to an aspect of the study. All AEs were classified as mild.

## Data Availability

The data collection code is available on GitHub (https://github.com/davepruitt/RePlay). All raw data have been included in the Supplementary Information and are available at Open Data Commons for Spinal Cord Injury (https://odc-sci.org/data/1315).
