# Supplementary material for: Closed-loop vagus nerve stimulation aids recovery from spinal cord injury
Source: Nature. 2025 May 21;643(8073):1030–6. doi: 10.1038/s41586-025-09028-5 (PMC12286844; doi:10.1038/s41586-025-09028-5)
Supplement: Supplementary file 1 — Description of the statistical methods and outcomes from statistical testing regarding assumptions of normality and linear mixed modelling. [file 41586_2025_9028_MOESM1_ESM.pdf]

nature portfolio

https://doi.org/10.1038/s41586-025-09028-5

Supplementary information

Closed-loop vagus nerve stimulation aids recovery from spinal cord injury

In the format provided by the authors and unedited

Supplemental Statistical Information

Part 1: Assumption Checks

Figure 4a and 4b: Pinch and Knob tasks

This approach uses the raw force/torque values from each participant's first and last day. The chosen statistical test therefore compares actual force/torque values at two timepoints (first day and last day).

Task	Jarque-Bera Test (First Day) p-value	Jarque-Bera Test (Last Day) p-value	Test used	Test result (p-value)
Pinch	**0.024**	**0.0048**	Wilcoxon signed-rank	**0.00013**
Knob	0.101	**0.0225**	Wilcoxon signed-rank	**0.00331**

This approach uses a *log2* transform of the raw force/torque values from each participant's first and last day.

Task	Jarque-Bera Test (First Day) p-value	Jarque-Bera Test (Last Day) p-value	Test used	Test result (p-value)
Pinch	0.5*	0.383	Paired t-test	**2.396×10^{-8}**
Knob	0.119	0.170	Paired t-test	**0.000784**

This approach first calculates a linear regression from each participant's data (using a *log2* transform on the data, which thus gets incorporated into the linear regression). It then uses the predicted first and last day values of force/torque using the linear regression model as the data being tested. This is the approach used in the main manuscript where the results from Figure 4a and Figure 4b are reported.

Task	Jarque-Bera Test (First Day) p-value	Jarque-Bera Test (Last Day) p-value	Test used	Test result (p-value)
Pinch	0.437	0.5*	Paired t-test	**5.653×10^{-8}**
Knob	0.212	0.182	Paired t-test	**0.00645**

* A p-value of 0.5 is the maximum p-value that can be represented with the Jarque-Bera test.

Figure 5a: Delta GRASSP Score

Group	Mean +/- Std Err	Jarque-Bera Test p-value	Test used	Test result (p-value)
No VNS for 18 sessions	-1.44 +/- 1.877	**0.001**	Wilcoxon signed-rank	0.172
Yes VNS for 18 sessions	2.658 +/- 1.472	0.167	Paired t-test	0.087
Yes VNS for 18-36 sessions	4.079 +/- 1.465	0.365	Paired t-test	**0.012**
Yes VNS for 36 sessions	5.05 +/- 2.085	0.5*	Paired t-test	**0.038**

* A p-value of 0.5 is the maximum p-value that can be represented with the Jarque-Bera test.

Figure 5c: GRASSP score before and after CLV

Timepoint	GRASSP Mean +/- Std Err	Jarque-Bera Test p-value
Before CLV	65.605 +/- 4.052	0.5*
After CLV	69.684 +/- 3.831	0.5*

Because both the before and after timepoints had p-values of 0.5, a paired t-test was used to compare the data between the two timepoints. The resulting paired t-test had a p-value of **p = 0.0122**.

* A p-value of 0.5 is the maximum p-value that can be represented with the Jarque-Bera test.

Figure 5f: Delta GRASSP Motor Subscore

Group	Mean +/- Std Err	Jarque-Bera Test p-value	Test used	Test result (p-value)
No VNS for 18 sessions	-0.33 +/- 1.167	0.055	Paired t-test	0.782
Yes VNS for 18 sessions	1.263 +/- 0.927	0.174	Paired t-test	0.189
Yes VNS for 18-36 sessions	2.105 +/- 0.677	0.097	Paired t-test	**0.0061**
Yes VNS for 36 sessions	2.6 +/- 0.765	0.5*	Paired t-test	**0.0078**

* A p-value of 0.5 is the maximum p-value that can be represented with the Jarque-Bera test.

Part 2: Linear Mixed Modeling

For Figure 4 a linear mixed model was fitted to the data using Matlab. Before fitting a model, the data was transformed using a *log2* transform. The model used was:

y ~ 1 + *day* + (1 | *subject*)

Therefore, our model included task day (or "time") as a fixed effect while also including each subject as a random effect to account for variation in each subject's individual slope. This model was used for each task/panel shown in Figure 4. The resulting p-values from running an ANOVA on the linear model are given below:

**Task**	**Linear mixed-model p-value**
Pinch	**2.853×10^{-101}**
Knob	**2.717×10^{-41}**
Grip	**6.383×10^{-61}**
Finger Extension	**2.584×10^{-19}**
Index Abduction	**0.000554**
Touchscreen	**2.362×10^{-8}**
Typing	**9.702×10^{-11}**
Range of Motion Handle	**1.8×10^{-10}**
Hand Opening	**2.17×10^{-14}**

IRB Project Number: 019-356

Targeted Plasticity Therapy for Upper Limb Rehabilitation in Spinal Cord Injuries

Study Protocol

Rita Hamilton, DO

Richard Naftalis, MD, FAANS, FACS

Michael Foreman, MD FACS

Mark Powers, PhD

Ann Marie Warren, PhD

Jane Wigginton, MD

Robert Rennaker, PhD

Michael Kilgard, PhD

Seth Hays, PhD

Holle Carey, MS

Baylor University Medical Center (BUMC)
Baylor Scott & White Institute for Rehabilitation
&
Division of Trauma
Dallas, Texas

Texas Biomedical Device Center
University of Texas at Dallas
Richardson, Texas

1. Background and General Aims

Targeted Plasticity Therapy for Upper Limb Rehabilitation in Spinal Cord Injuries (SCI) is a federally funded study previously sponsored by the Defense Advanced Research Projects Agency (DARPA: a division of the Department of Defense). The study will provide an initial evaluation of the use of the ReStore implanted stimulator in the context of spinal cord injuries. The study will help to assess the prospect that VNS coupled with rehabilitation in participants suffering from upper extremity paresis after a spinal cord injury is a potentially effective treatment to improve recovery of upper limb motor function.

This technique uses brief pulses of VNS to engage pro-plasticity neuromodulatory circuits during rehabilitation exercises. Brief trains of VNS or placebo stimulation are paired with movements during standard upper limb rehabilitation exercises that are individualized to each participants' level of function in SCI individuals.

The study design will be a prospective, double blind, randomized placebo controlled, plus open-label extension trial conducted over a 6 month period in 20 spinal cord injured individuals. Participants for the study will include 20 participants with cervical SCI and will be randomized into either interventional group (rehabilitation with active VNS) or control group (equivalent rehabilitation with placebo stimulation). Safety and efficacy will be assessed in all participants, regardless of whether they elect to exit the study at the end of Phase 1 or continue to the Phase 2 open-label extension, twice per year for up to 2 years after the date of implantation.

Study Rationale

Texas Biomedical has developed an innovative strategy to enhance recovery of motor and sensory function after neurological injury termed targeted plasticity therapy (TPT). This technique uses brief pulses of vagus nerve stimulation to engage pro-plasticity neuromodulatory circuits during rehabilitation exercises (Engineer et al., 2011; Hays et al., 2013). Preclinical findings demonstrate that VNS paired with rehabilitative training enhances recovery in multiple models of neurological injury, including spinal cord injury, ischemic stroke, intracerebral hemorrhage, and traumatic brain injury (Engineer et al., 2011; Khodaparast et al., 2013, 2014, 2016, Hays et al., 2014a, 2014b, 2016; Pruitt et al., 2016; Ganzer et al., 2018; Meyers et al., 2018). Recovery is associated with neural plasticity in spared motor networks in the brain and spinal cord. Moreover, an early feasibility study and an independent, double-blind, placebo-controlled study in chronic stroke participants indicate that VNS is safe in participants with upper limb deficits, and yields a clinically-significant three-fold increase in recovery of upper limb function compared to rehabilitation without VNS. Here, the Sponsor proposes to extend these findings and evaluate whether VNS paired with rehabilitation represents a safe and feasible strategy to improve recovery of upper limb motor function in participants after spinal cord injury.

This double blind, placebo controlled randomized design with open-label extension will allow for the assessment of safety and feasibility of the ReStore system to deliver vagus nerve stimulation paired with rehabilitation in spinal cord injury participants. This study's aim is estimate the safety and feasibility, as well as the potential prospect of benefit in spinal cord injured individuals. The

ReStore system and therapy will be considered feasible if greater than 50% of the valid attempts to stimulate are successful per the ReStore system log.

Benefits of Targeted Plasticity Therapy:
A number of preclinical studies and evidence from two clinical trials support the notion that VNS paired with rehabilitation represents a potential method to enhance recovery in SCI patients. Clinical trials of VNS paired with rehabilitation have not yet been conducted in spinal cord injured individuals, so this study will be a first in human assessment of safety and feasibility, and is also expected to provide an initial estimate of sample size for a pivotal trial in this participant population. Given that VNS paired with rehabilitation has been shown in preclinical studies as well as a pilot clinical trial in stroke patients to enhance neural plasticity, if effective in participants with spinal cord injury, it may provide a route for participants to recover other functions that are impaired from their spinal cord injury, including bowel and bladder dysfunction.

Benefits of the New Device IPG:
Texas Biomedical has developed a low-cost, glass encapsulated implantable pulse generator (IPG) referred to as the ReStore device. This will be the first in human safety test of this specific IPG. This IPG does not have a battery, does not have leads, and can be reprogrammed wirelessly. The device has been shown to be MRI conditionally safe based on ASTM standards. All of these factors make the ReStore IPG a superior solution to current commercially-available IPGs Participants with the IPG will still be able to undergo standard clinical imaging techniques, and a surgery will not be required for battery replacement or lead repair/replacement, obviating several common issues associated with existing IPGs.

Background
Summary of Findings from Nonclinical in vitro and in vivo Studies

- TPT enhances recovery in unilateral model of C5/C6 contusion injury in rat compared to rehabilitation alone (*Ganzer et al., 2018*)
- TPT enhances recovery in bilateral model of C5/C6 contusion injury in rat compared to rehabilitation alone (*Ganzer et al., 2018*)
- TPT triples synaptic connectivity to rehabilitated muscles after spinal cord injury (*Ganzer et al., 2018*)
- TPT enhances recovery of forelimb motor function following a controlled cortical impact compared to rehabilitation alone (*Pruitt et al, 2015*)
- TPT enhances forelimb motor recovery after a severe subcortical intracerebral hemorrhage (ICH) compared to rehabilitation alone (*Hays et al, 2014*).
- TPT enhances recovery of forelimb strength (*Khodaparast et al, 2013*) and forelimb speed (*Khodaparast et al, 2014*) compared to rehabilitation alone after ischemic stroke.
- TPT is effective in aged participants (*Hays et al, 2016*) and when initiated long after injury (*Khodaparast et al, 2016*).
- TPT results in long-lasting recovery of motor function, generalization of rehabilitation benefits, and substantial plasticity in motor networks after ischemic stroke (*Meyers et al, 2018*).
- TPT engages the pro-plasticity noradrenergic and cholinergic networks to promote plasticity (*Hulsey et al, 2016*; *Hulsey et al, 2017*).

Summary of Human Use

VNS has been used in over 90,000 people for the treatment of intractable epilepsy. Similar VNS protocols have also been safely delivered in participants with depression, heart failure, obesity, bipolar treatment-resistant depression, Alzheimer's disease, essential tremor, Landau-Kleffner syndrome and autism. In these indications, VNS is used throughout the day and is not contingent with rehabilitative training. This continuously cycling stimulation follows a duty cycle typically 30 s ON, 5 min OFF, for 24-hours a day – a total of about 130 minutes per day. Stimulation parameters typically range from 1.0 to 3.0 mA; 250 to 500 µs pulse width at 20 Hz.

**VNS for Epilepsy**	**VNS for Targeted Plasticity Therapy**
~130 minutes of stimulation each day	~3-5 minutes of stimulation during each rehabilitation session
1mA - 2mA	~ 0.8 mA
7 days a week	3 days a week
24 hours a day	~2 hours on therapy days
30 second long stimulation train	0.5 second long stimulus train

In comparison, the VNS stimulation paradigm used in targeted plasticity therapy consists of 0.5 s trains of 0.8 mA 100 µs biphasic pulses delivered at 30 Hz. Stimulation trains are delivered only during rehabilitative sessions. In previous implementations for stoke and tinnitus, patients receive approximately 300-600 stimulations per rehabilitative session, equivalent to 3-5 total minutes of vagus nerve stimulation each day. The current study will deliver a similar amount of stimulation. This stimulation is well within the established safety guidelines with a duty cycle less than 50% (Agnew et al., 1989). Because of the lower pulse width, lower amplitude, and substantial reduction in train duration and number of trains, the proposed use of VNS in this study will deliver less than 1% of the total daily charge compared to existing FDA-approved applications for epilepsy. To provide an additional level of safety, the ReStore system limits the duty cycle to 10%. Together, it is expected that the substantial reduction in amount of stimulation will further reduce the side effect profile and increase tolerability of VNS therapy.

TxBDC has completed four early feasibility studies using paired VNS therapy in humans (*NCT1962558*; *NCT01253616*; *NCT01669161; NCT02243020*) and is currently participating in the fifth IDE, a pivotal study using VNS paired with rehabilitation in stroke patients (*NCT03131960*). Each of the completed studies has demonstrated safety and efficacy of TPT for the treatment of tinnitus (De Ridder et al., 2013; Tyler et al., 2017) and recovery of motor and sensory function following ischemic stroke (Dawson et al., 2016; Kilgard et al., 2018).

In the tinnitus trials, the therapy was well tolerated, and no participant withdrew from the study due to complications or side effects. In the first trial, four of the ten participants exhibited clinically meaningful improvements in their tinnitus, both for the affective component, as quantified by the Tinnitus Handicap Inventory, and for the sound percept, as quantified by the minimum masking level. These improvements were stable for more than two months after the end of therapy. Of the six participants that did not exhibit improvements, five were on medications that included muscarinic antagonists, norepinephrine agonists, and γ-amino butyric acid agonists, thereby

possibly interfering with acetylcholine and norepinephrine release induced by vagus nerve stimulation (VNS) and essential for inducing plasticity (Hulsey et al., 2016, 2017). These participants had no improvement in contrast to medication-free participants (De Ridder et al., 2013). A second trial demonstrated safety in an additional 30 participants with tinnitus received active VNS, with more than 50% of patients demonstrating a clinically meaningful response at one year (Tyler et al., 2017).

In the initial study examining TPT for stroke, twenty-one participants with ischemic stroke >6 months before and moderate to severe upper-limb impairment were randomized to receive VNS plus rehabilitation or rehabilitation alone (*NCT01669161*). Rehabilitation consisted of three 2-hour sessions per week for 6 weeks, each involving >400 movement trials. In the VNS group, movements were paired with 0.5 second VNS. The primary objective was to assess safety and feasibility. Secondary end points included change in upper-limb measures (including the Fugl–Meyer Assessment-Upper Extremity). Nine participants were randomized to VNS plus rehabilitation and 11 to rehabilitation alone. There were no serious adverse device effects. One participant had transient vocal cord palsy and dysphagia after implantation. Five had minor adverse device effects including nausea and taste disturbance on the evening of therapy. In the pre-specified analysis, there was a significant difference in change in Fugl–Meyer Assessment-Upper Extremity score (between-group difference, 6.5 points; 95% confidence interval, 0.4 to 12.6). In a second randomized, blinded study, 8 participants received active VNS and 9 received placebo. At 90 days post-therapy, active VNS resulted in a trend toward improved UE-FM scores compared to placebo ($p = 0.055$) (*Kimberley et al., 2018*). Participants in the placebo group then crossed over to receive active VNS and demonstrated a significant improvement in UE-FM scores after active therapy compared to after placebo ($p < 0.01$).

Summary of Use of Targeted Plasticity for Treating Motor Deficits in Spinal Cord Injury
It is well understood that the human central nervous system has the capability of undergoing a significant degree of plasticity in both healthy and diseased states. Following insults such as spinal cord injury, plasticity in spared networks can improve function to some degree. Building on this, the ability to augment plasticity in conjunction with rehabilitation represents a potential readily-translatable avenue to enhance recovery after SCI. Multiple mechanistically-distinct animal models of spinal cord injury demonstrate substantially improved recovery and a three-fold increase in synaptic connectivity to rehabilitated muscles after SCI. This is consistent with significant, lasting improvements in recovery and enhanced plasticity in multiple animal models of ischemic stroke, intracerebral hemorrhage, and traumatic brain injury. Moreover, pilot clinical studies provide initial evidence that VNS paired with rehabilitation is safe, tolerable, and enhances recovery of upper limb motor function in chronic stroke participants. Based on these data, a trial of VNS therapy for SCI participants that could improve long term functional recovery is now timely, and may stand to dramatically change clinical practice in the near future.

Importance of the Study and Any Relevant Treatment Issues or Controversies
Spinal cord injury (SCI) places an enormous burden on participants, their families, and society at large. In addition to the emotional and psychological suffering experienced, low tetraplegics direct lifetime costs directly attributable to their spinal cord injury (SCI) of between approximately $2 - $4 million each, which does not include loss of wages and productivity. Current medical care and

rehabilitation lack consistently effective post-lesion interventions to reduce disability and improve quality of life.

In response to this substantial need, TxBDC has developed an innovative technique to enhance recovery of motor and sensory function after neurological injury. To date, preclinical studies involving numerous animal models have shown safety and efficacy of targeted plasticity therapy for neurological injuries, including SCI. Early clinical trials of VNS paired with rehabilitation in stroke participants have also shown safety, and pointed towards signals of efficacy. Therefore, it is timely to begin translation of this technology to participants with SCI.

Earlier devices aimed at utilizing VNS for enhanced recovery suffered from having an internal battery system that required surgery when the power was depleted, thus TxBDC has developed a system with an external power source that should allow for a single implantation surgery. Additionally, early devices were not known to be MRI compliant, which was a deterrent to participants, as MRIs are being employed to evaluate more and more disease processes, both those associated with their neurologic injury as well as a host of others. The ReStore System is MRI conditional (ASTM F2213; ASTM F2182; ASTM F2052, ASTM F2119; ASTM F2503), allowing participants to receive the imaging necessary to monitor their health status.

2. Specific Objectives

OBJECTIVES	ENDPOINTS	JUSTIFICATION FOR ENDPOINTS
Primary		
Estimate safety	Adverse events	Review of adverse events reported throughout the trial will be used to inform the potential risks associated with the ReStore system and provide a better understanding of risk/benefit analysis
Secondary		
Demonstrate the system is feasible to use during rehabilitation	Greater than 50% of the valid attempts to stimulate are successful per the ReStore system log.	If stimulation delivery is successful during active portion of therapy sessions then the system is feasible to use.
Measure changes in force/torque as a result of VNS paired rehabilitation	10% increase in finger pinch and flexion force following active VNS<br>10% increase in wrist flexion and extension force following active VNS<br>10% increase in wrist pronation and supination force following active VNS	Efficacy measurement to use as basis for estimate of sample size for a pivotal trial

OBJECTIVES	ENDPOINTS	JUSTIFICATION FOR ENDPOINTS
Measure changes in GRASSP score	>4 point shift in GRASSP assessment following active VNS	Efficacy measurement to use as basis for estimate of sample size for a pivotal trial

3. Participant Selection Criteria

Twenty participants, ages 18-64 years with cervical SCI at least a twelve months prior to study enrollment, resulting in an ASIA grade B, C, or D, and grade 1 or better motor function as described by the International Standards for Neurological Classification of Spinal Cord Injury (ISNCSCI). These will be recruited from Baylor Rehabilitation Institute's current patients, partnering rehabilitation facilities, and from the community at large.

Inclusion Criteria
In order to be eligible to participate in this study, an individual must meet all of the following criteria:

- Provision of signed and dated informed consent form
- Stated willingness to comply with all study procedures and availability for the duration of the study
- Adult, aged 18-64
- In good general health as evidenced by medical history and diagnosed with first time cervical spinal cord injury resulting in an ASIA grade B, C, or D, and level 1 or better motor function as described by the International Standards for Neurological Classification of Spinal Cord Injury (ISNCSCI).
- SCI caused by trauma that occurred ≥ 12 months prior to enrollment
- Meets all clinical criteria for the surgical VNS implantation as determined by the PI, surgeon, and anesthesiologist
- Must demonstrate some residual upper limb and hand movement in either arm
- Appropriate candidate for VNS implantation
- Willing and able to comply with the study protocol

Exclusion Criteria
An individual who meets any of the following criteria will be excluded from participation in this study:

- Spinal cord injuries by sharp objects, firearms, and non-traumatic or congenital causes, even if at different levels of the spinal cord
- Participants with prior right-sided anterior cervical surgery will require laryngoscopy prior to randomization. Those with evidence of recurrent laryngeal nerve injury will be excluded.

- Participants with prior left sided anterior cervical surgery will be eligible, regardless of prior recurrent laryngeal nerve injury. However, if at surgery there is too much scar tissue for safe implantation the surgery will be abandoned to ensure participant safety
- Concomitant clinically significant brain injuries
- Prior injury to vagus nerve
- Prior or current treatment with vagus nerve stimulation
- Participant receiving any therapy (medication or otherwise) that would interfere with VNS
- Pregnancy or lactation
- Clinical complications that hinder or contraindicate the surgical procedure
- Psychiatric disorders, psychosocial, and/or cognitive impairment that would interfere with study participation, as assessed by medical evaluation
- Abusive use of alcohol and/or illegal substances use
- Participation in other interventional clinical trial
- Participants with known immunodeficiency including participants who are receiving or have received chronic corticosteroids, immunosuppressants, immunostimulating agents or radiation therapy within 6 months
- Participants with significant comorbidities or conditions associated with high risk for surgical or anesthetic survival (e.g. renal failure, peripheral vascular disease, unstable cardiac disease, poorly controlled diabetes, immunosuppression, etc.).
- Participants with active neoplastic disease.
- Participants with significant local circulatory problems, (e.g. thrombophlebitis and lymphedema, and clinically-significant hypotension or bradycardia).
- Participants with any medical condition or other circumstances that might interfere with their ability to return for follow-up visits in the judgment of the Investigator.
- Any condition which, in the judgment of the Investigator, would preclude adequate evaluation of device’s safety and performance.
- Aphasia and other cognitive deficits may be present but participants will be excluded if are unable to understand the potential risks and benefits of the study or personally provide informed consent.
- A recent history of syncope
- A recent history of dysphagia
- Currently require, or are likely to require diathermy
- Significant respiratory issues that would interfere with participation
- Non-English speaking
- Patients who are acutely suicidal and/or have been admitted for a suicide attempt
- Incarceration or legal detention

4. Study Schedule

Subjects that align with inclusion/exclusion criteria and sign informed consent documents will undergo surgical implantation of the ReStore stimulator on the left cervical vagus nerve. After a minimum of one week after surgery, participants will undergo a baseline assessment and be randomized 1:1 into one of two groups. During phase 1, the first group will receive active VNS

paired with rehabilitation for 18 sessions of in-office rehabilitation over the course of six weeks (Immediate Start VNS group). The second group will receive equivalent in-office rehabilitation with placebo stimulation according to the same visit schedule. (Delayed Start VNS group).

All participants will undergo approximately three sessions per week with a physical therapist or occupational therapist. Each session will be approximately an hour long. During rehabilitation, the power and control module (PCM) of the ReStore system will be placed in a band around the participant's neck with the coil positioned over the implanted stimulator. The therapist will perform standard rehabilitative exercises and VNS will be triggered coincident with arm movement, as in our previous studies. Each session consists of standard rehabilitation exercises, including motor and sensory integration exercises, but may also include devices to measure upper limb strength, range and flexibility. All stimulation parameters will be controlled by the software, based on participant group assignment, to allow the therapist to be blinded to the treatment group. At the beginning of each session, subjects in both groups will receive placebo stimulation consisting of a descending level of stimulation intensity (0.8, 0.6, 0.4, 0.2, 0.1, 0 mA) delivered on the first six movements during rehabilitative exercises. Participants will be instructed that they may initially perceive stimulation, but the perception may fade. This serves to better blind participants and therapists to stimulation group. After the initial placebo stimulation ramp, participants in the active stimulation group will receive 0.5 s trains of stimulation at 0.8 mA with each button press by study personnel (limited to a 25% duty cycle). The stimulation parameters may be modified within the stated safety limits based on the discretion of the overseeing physician. Based on our previous studies, we expect that participants will receive stimulation approximately 5-10 times per minute, equating to approximately 300 stimulations during an in-office rehabilitation session.

During Phase 1, all participants will receive six weeks of rehabilitation with either active or placebo VNS, in accordance with their randomization. At the end of Phase 1, all participants will undergo mid-therapy assessment (Assessment 2) and will be provided the option to exit the study or continue to Phase 2 during which all participants will receive active stimulation, regardless of prior group assignment. Rehabilitation with active VNS will continue for an additional eighteen in-office sessions during Phase 2. Following the completion of the final session of rehabilitative training in Phase 2, an assessment will be performed (Assessment 3). Long-term assessment of safety will be performed twice yearly for up to 2 years after the implant date when possible.

Phases:

Pre-Therapy: During the first six weeks, participants will undergo consent, assessment, surgical implantation, and group assignment. Participants must wait a minimum of two weeks after surgery to start rehabilitation.

Phase 1: The Immediate Start VNS group will receive rehabilitation and active stimulation for 18 in-office sessions over the course of approximately 6 weeks. The Delayed Start VNS group will receive equivalent rehabilitation with placebo stimulation. An assessment will be performed approximately one week after the completion of the 18 sessions of rehabilitation.

Phase 2: Subjects who do not exit the study at the end of Phase 1 will participate in an open-label extension consisting of an additional 18 sessions of in-office rehabilitation with active VNS over

the course of approximately 6 weeks. Participants will be assessed approximately 1 week after the conclusion of the additional 18 sessions of therapy.

Long-term Follow Up: Safety will be assessed in all participants, regardless of whether they elect to exit after Phase 1 or continue into the Phase 2 open-label extension, twice per year for up to 2 years after the date of implant. Participants will have the option to leave the device implanted, and participants may be offered the opportunity to participate in additional studies. Aside from participation in additional studies, there is no plan to stimulate during this period. Safety assessments will only report on the presence of the implanted device and/or participant status during this period. Participants who experience a serious device related adverse event will be removed from the identified risk, but will continue to be monitored for 2 years or longer if the event has not resolved.

Schedule of Activities (SOA)

	Pre therapy				Phase 1							Phase 2 OLE						
Visit Number	1	2	3	4	5-7	8-10	11-13	14-16	17-19	20-22	23	24-26	27-29	30-32	33-35	36-38	39-41	42
Approximate Week Number	1	4	4	6	7	8	9	10	11	12	13	14	15	16	17	18	19	20
Sign Informed Consent[1,2,3]	x																	
Medical History[4]	x																	
Physical Exam[5,6]	x																	
Laryngoscopy (if applicable)[7]	x																	
Pre-Op Assessment[8]		x																
Implantation[9]			x															
Post-Op and Stimulation Test[10,11]				x														
Vital Signs[12]	x	x	x	x	x	x	x	x	x	x	x	x	x	x	x	x	x	x
Collect Adverse Events[13,14,15]	x	x	x	x	x	x	x	x	x	x	x	x	x	x	x	x	x	x
Collect Device Setting Information[11]				x	x	x	x	x	x	x	x	x	x	x	x	x	x	x
Complete Case Report Forms[5,16,17,18,19,20,31,32]	x	x	x	x	x	x	x	x	x	x	x	x	x	x	x	x	x	x
Therapy																		
Rehabilitation[11]					x	x	x	x	x	x		x	x	x	x	x	x	
Vagus Nerve Stimulation (in Active Group)				x	x	x	x	x	x	x		x	x	x	x	x	x	
Assessments																		
International SCI UE Basic Data Set[17]	x										x							x
ASIA Assessment[22]	x										x							x
SCI Independence Measure III[23]	x										x							x
International SCI Pain Basic Data Set[24]	x										x							x
Patient Health Questionnaire 9[25]	x			x							x							x
Hospital Anxiety and Depression Scale[26]	x			x							x							x
GRASSP[27]	x			x							x							x
Jebsen-Taylor Assessment[28]	x			x							x							x
Force and Range of Motion Assessments[29]	x			x							x							x
Participant Satisfaction Survey[30]											x							x

Forms:

1. Informed Consent (CSF-SCI-FDA-001)
2. Inclusion Exclusion Criteria (CRF-SCI-009)
3. Informed Consent Documentation Process (CRF-SCI-010)
4. Demographics and Medical History (CRF-SCI-003)
5. Baseline Signs and Symptoms (CRF-SCI-001)
6. Physical Exam (CRF-SCI-017)
7. Laryngoscopy (CRF-SCI-026)
8. Pre-Operative Assessment (CRF-SCI-019)
9. Surgical Implantation (CRF-SCI-024)
10. Post-Operative Assessment (CRF-SCI-018)
11. Rehabilitation Note and Stimulation Device Programming (CRF-SCI-025)
12. Vital Signs (CRF-SCI-037)
13. Narrative Adverse Event Report Form (CRF-SCI-027)
14. Participant Adverse Event Log (as applicable) (CRF-SCI-028)
15. Study Adverse Event Log (as applicable) (CRF-SCI-029)
16. Concomitant Medications (CRF-SCI-002)
17. Device Complication (as applicable) (CRF-SCI-004)
18. Device Removal (as applicable) (CRF-SCI-005)
19. Study Completion Withdrawal (as applicable) (CRF-SCI-022)
20. Study Deviation Log (as applicable) (CRF-SCI-023)
21. International SCI UE Basic Data Set (CRF-SCI-012)
22. ASIA Assessment (CRF-SCI-031)
23. SCIM III (CRF-SCI-021)
24. International SCI Pain Basic Data Set (CRF-SCI-011)
25. Patient Health Questionnaire 9 (PHQ 9) (CRF-SCI-016)
26. Hospital Anxiety and Depression Scale (HADS) (CRF-SCI-008)
27. GRASSP Scores (CRF-SCI-006)
28. Jebsen-Taylor Hand Function (CRF-SCI-013)
29. Quantitative Force and Range of Motion Assessment (CRF-SCI-030)
30. Participant Satisfaction Survey (CRF-SCI-014)
31. Device Information Log (CRF-SCI-043)
32. Reimbursement Log (CRF-SCI-032)

Participant Flow

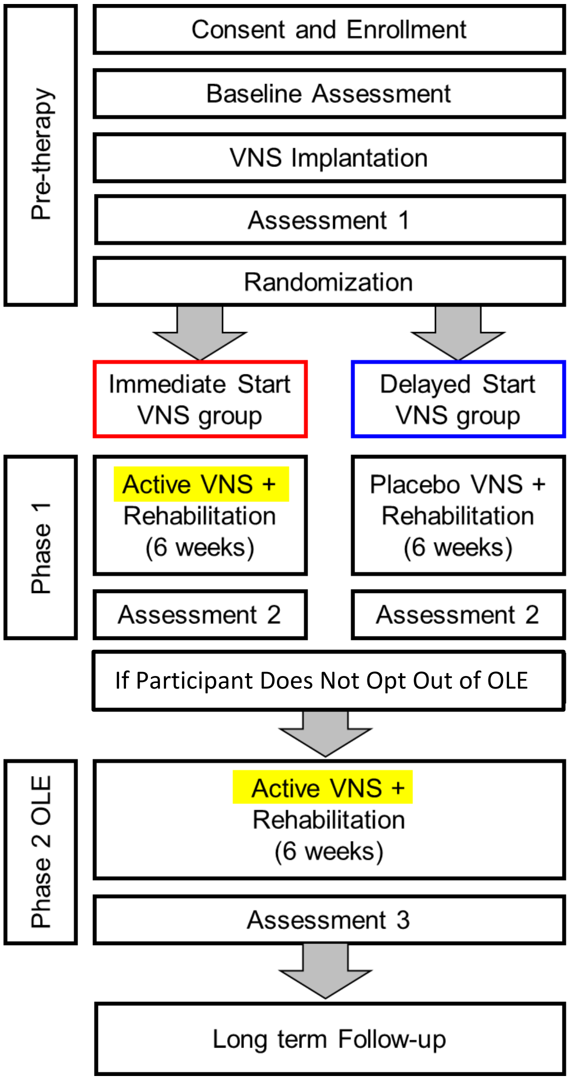

5. Measures (see appendix for all measures)

Demographic Data

Participant demographic variables will be obtained through a standard self-report form or the patient's medical record at baseline and will include age, race, ethnicity, marital status, education level, employment, income, insurance status, veteran status, premorbid psychiatric history, and history of substance use.

Safety Screening and other Assessments

Medical History: Each participant will have a medical history evaluated during participant recruitment. As part of this history, the PI will review all medications that might interfere with the study efficacy.

Physical examinations may include:

- Medical history
- Demographics
- History of injury and care
- Physical assessment of health
- EKG may be required to screen for surgical eligibility
- Clinical assessments and examinations, including:
    1. International SCI Upper Extremity Basic Data Set
    2. International Standard for Neurological Classification of Spinal Cord Injury
    3. Jebsen-Taylor Hand Function Test
    4. Graded Redefined Assessment of Strength, Sensibility, and Prehension (GRASSP)
    5. Spinal Cord Injury Independence Measure (SCIM) III
    6. International SCI Pain Basic Data Set
    7. Hospital Anxiety and Depression Scale (HADS)
    8. Participant Health Questionnaire 9 (PHQ-9)
    9. Quantitative Force and Range of Motion Assessment
- Confidentiality and Privacy
- Rehabilitative devices

Data will be collected using case report forms designed specifically for this study. Each test will have a specific form or portion of a form designated to collect appropriate information. Upon completion, data will be entered as it is obtained into a central database maintained by the TX Biomedical Device Center at UT Dallas or their designee.

Measures to Minimize Bias: Randomization and Blinding

All participants will be implanted. The first ten patients will be block randomized in blocks of two to receive either Immediate Start VNS or Delayed Start VNS. The remaining ten subjects will be dynamically allocated to minimize the between-group difference in GRASSP score.. All participants will be implanted. All stimulation parameters will be programed by the engineering team at TxBDC and controlled by the software based on participant group assignment, allowing the clinician, physical therapist, and participants to remain blinded. At the beginning of each in-

office session, participants in the placebo group will receive sham stimulation consisting of a descending level of stimulation intensity delivered on the first five movements during rehabilitative exercises, after which intensity will be set to zero. Participants will be instructed that they may initially perceive stimulation, but the perception may fade. This procedure provides an effective method to blind the participants.

Efficacy Assessments

As a secondary endpoint, TxBDC will explore an estimation of the sample size required for a sufficiently powered pivotal trial. The following assessments may be used to measure the therapeutic improvement and were selected to sample functional arm and hand movements, and have been shown to be sensitive to rehabilitation and demonstrated reliability.

1. International SCI Upper Extremity Basic Data Set is a six-item data collection form designed to facilitate consistent collection and reporting of basic upper extremity findings in people with spinal cord injuries (Biering-Sørensen et al., 2014).
2. International Standard for Neurological Classification of Spinal Cord Injury (upper extremity) AIS/ ASIA is a worksheet and list of steps for determining the classification of individuals with spinal cord injury (Kirshblum et al., 2011). The worksheet includes evaluation of motor and sensory function, and neurological levels.
3. Jebsen-Taylor Hand Function Test is a seven-item test of hand functions commonly used in activities of daily living (Jebsen et al, 1969). The items tested include range of fine motor, weighted and non-weighted hand function activities.
4. Graded Redefined Assessment of Strength, Sensibility, and Prehension (GRASSP) is a measure used for spinal cord injury patients. The measure includes five subsets of questions designed to quantitatively measure clinical upper limb impairment (Kalsi-Ryan et al., 2012).
5. Spinal Cord Injury Independence Measure (SCIM) III is a 17-item measure designed to look at areas of function in spinal cord injury patients. The measure is scored on a scale from 0 to 100, where 0 is total dependence and 100 is complete independence (Catz et al., 2007; Itzkovich et al., 2007).
6. International SCI Pain Basic Data Set is a 10-item questionnaire that aids in collecting consistent reporting of pain in spinal cord injury patients (Widerström-Noga E et al., 2014).
7. Hospital Anxiety and Depression Scale (HADS) is a 14-item self-report scale created to detect depression and anxiety in healthcare settings (Zigmond & Snaith, 1983).
8. Participant Health Questionnaire 9 (PHQ-9) is a brief self-report measure of major depressive disorder (Kroenke et al, 2001). The PHQ-9 is considered to be a valid measure of depression for population-based studies and clinical populations (Corson, Gerrity, & Dobscha, 2004) with a cut off score of equal or greater than 10 as the diagnostic for current depression.
9. The Quantitative Force and Range of Motion Assessment form is used to record which assessments the therapist used to collect data on participant force and range of motion.

Study Intervention Compliance

The participants will be required to come to the study site for in-office rehabilitation, or study staff may deliver therapy in their home or living facility. The investigators will provide study visit reminders the night before an in-office session when possible. All sessions will be logged. The number of stimulations and duration of the therapy will be recorded.

6. Devices

Device Description

The therapy system consists of two components. The first component is the ReStore stimulator, which is a small glass encapsulated implantable pulse generator measuring 13 x 8 x 3.5 mm. It is placed in a medical grade silicone rubber cuff to maintain contact with the left cervical vagus nerve when implanted. The second component is the external power and communication module (PCM), which is worn in a cloth band around the neck. The PCM is placed above the IPG during therapy and provides wireless power and commands to trigger preprogrammed stimulation via the IPG. A secure application running on a smart device can be used to send a trigger to the PCM during therapy to initiate a stimulation.

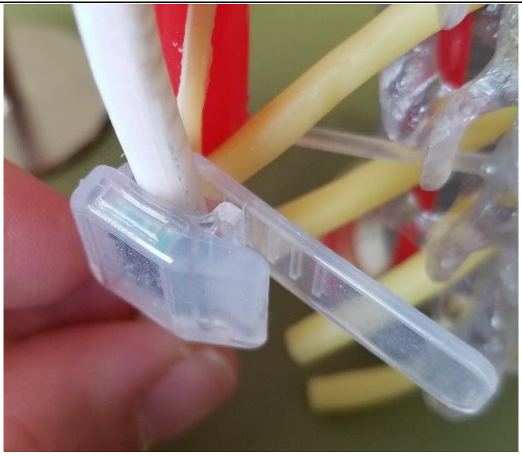	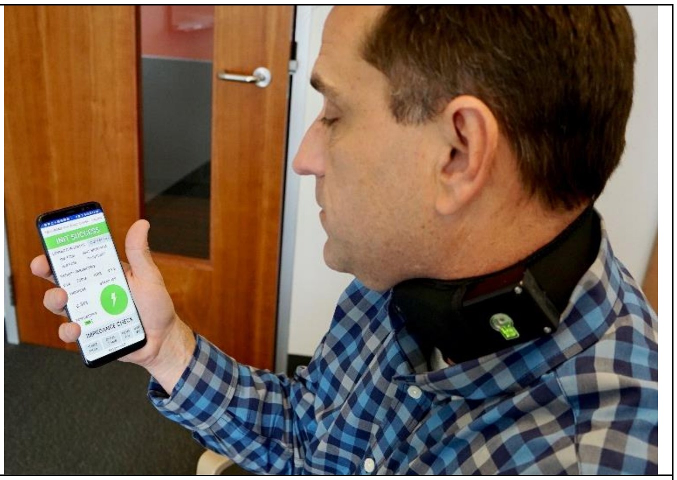
The IPG placed inside the silicone cuff. The white cable depicts the orientation of the nerve.	A smart device running the stimulation software application and the PCM in the neckband

Accessories

Rehabilitation Devices: TxBDC has developed rehabilitative devices that are similar to standard rehabilitation exercises commonly used in those with SCI that isolate upper limb forces and use those quantitative physical measures to trigger the smart device application to precisely control the time of stimulation.

FitMi Device: Accessories to rehabilitation. This commercially-available system from FlintRehab consists of two pucks participants will manipulate to control custom video game software. The participant manipulates the pucks to play the games to perform self-guided rehabilitation.

Device Traceability

Acquisition and accountability: The study devices will be stored in a locked room in a locked cabinet under the supervision of the study investigators. All devices will be appropriately labeled indicating they are investigational devices for use on this specific study only. The implantable

devices will be brought by study personnel to the surgery location and will not be stored at the hospital. A study storage and disposition log will be provided to the site to record study ID number.

Formulation, Appearance, Packaging, and Labeling: TxBDC at UT Dallas manufactures all devices. The IPGs come in a sealed, sterile pouch with an outer box. The label contains the lot number, sterilization date, sterilization expiration and the serial number of the device. Instructions for use will be provided to the study sites. The label will say "CAUTION - Investigational Device. Limited by Federal (or United States) law to investigational use."

Product Storage and Stability: The PCM and smart device will be kept in a locked cabinet in normal indoor conditions. Prior to implantation, the IPG and cuff will be stored as described above.

7. Patient Consent

The investigators will inform individuals about the aim of the study, the requirements (e.g., surgery, completing the questionnaires and subsequent sessions), and then invite them to participate. Those who agree to participate will be provided with an informed consent form. Consent will be obtained in a private room. Participants considered "special populations" that are unable to physically sign (i.e., tetraplegia) will verbally be able to provide consent and the consent form will be signed by a third party non-biased witness (i.e., patient advocate or guest relations). Family and friends may take part in the consent process but will not be allowed to sign or co-sign the consent form on behalf of the participant.

For this initial pilot study, we will enroll only English-speaking participants. The original signed consent will be retained in each participants' study file, and a copy of the signed form will be returned to the participant.

8. Trial Design

Screening

Participants will be screened and assessed for upper limb paresis associated with spinal cord injury using a detailed spinal cord injury history evaluation (including age of onset and neurological evaluation, etc.) and various screening scores will be collected to determine the level of disability in performing everyday tasks.

The first visit will be to determine eligibility. A physician will review the medical history and perform a physical and neurological exam. If they meet the inclusion criteria and sign the informed consent form, the participant will be scheduled for surgery and given a schedule of the study visits as well as instruction to prepare them for each visit. Participants must be able to give their own consent. After screening, enrollment, informed consent and surgery, participants will undergo the full set of assessments prior to randomization.

Biological Specimen Collection

Lab studies may be required to screen for surgical eligibility. Blood pregnancy tests will be collected in females ≤ 50 years of age. This is due to the blood test being more sensitive than a urine test.

Assessment of Study Agent Adherence

Each participant's progress and adherence will be monitored during the therapy. The computer used to activate the stimulator tracks the stimulus parameters, number of stimulations and number of sessions.

Administration of Participant-Reported Outcomes

Participants may be interviewed at each office visit. Interviews may be video recorded and reviewed . Study investigators will document any participant reported issues in their study records.

Implantation Procedures

Implantation: The participants will be placed in a supine position, administered general anesthesia, and intubated. A transverse incision will be made through the skin and the platysma in the left lateral neck at approximately the level of the cricoid. The carotid triangle will be carefully dissected, and the carotid sheath will be opened to expose the carotid artery and the vagus nerve. Hemostasis will be achieved without the use of metal vascular clips. One to two inches of the vagus nerve will be bluntly dissected circumferentially. The depth of the vagus nerve from the skin will be measured and recorded at the location where the implant will be placed.

The IPG will be placed into its cradle in the silicone cuff. Then the flap of the silicone cuff will be introduced between the carotid and vagus nerve utilizing a lateral to medial approach, such that the base of the cuff faces the superficial, dermal side. The flap will then be passed through the loop on the cuff to close the cuff such that the cuff contacts the entire circumference of the vagus nerve without impinging on it. Prior to closure, a PCM will be placed over the implant and a communication check will be performed. If the device fails to communicate after multiple trials, it will be replaced. The replacement of a device during the initial surgery does not qualify as a failure with regards to safety outcome measures. Once communication at the implant depth has been verified, the excess length of the silicone flap will be trimmed, and the remaining end of the flap will be sutured to the base of the cuff to secure the cuff around the nerve. The cuff will then be rotated such that flat surface of the base is facing outwards towards the skin. A photo will be taken of the implanted cuff prior to closing the surgical site to verify position and orientation. The incision will then be closed in layers with sutures and/or medical skin glue. No staples will be utilized in the closure of the surgical incision.

Device Removal

If medically required, or if the participant elects, the IPG will be explanted at no cost to the participant or their insurance within up to 2 years of their date of implant. Beyond these two years, the participant and/or their insurance will be responsible for the costs associated with explantation.

After appropriate surgical preparation and anesthesia, the participants will be placed in a supine position. An incision, at the original site, will be made through the skin and the platysma. The carotid triangle will be dissected, and the carotid sheath will be opened near the implant. The implant identified and photographed in situ. Any capsular tissue overlying the cuff will be carefully dissected, the sutures that secure the flap of the cuff will be cut to allow the cuff to open. The cuff

will be then carefully removed from under the nerve. The wound will be closed in layers with sutures and/or medical skin glue. The device and cuff will be placed in a vial with physiological saline and returned to the manufacturer.

Dosing and Administration

The total charge delivered per day is considerably less than that that is currently FDA-approved for epilepsy treatment. VNS will be delivered during rehabilitative exercises as appropriate for each group. Stimulation parameters for active and placebo conditions will be software controlled to ensure blinding. During in-office rehabilitative sessions, study personnel will initiate a train of stimulation at up to 0.8 mA by pressing a button during arm and hand rehabilitation exercises. Participants may also perform computer-based rehabilitative exercises where the software sends stimulation trigger requests at specified times to coincide with arm and hand movement. Each participant should receive a total of approximately 300-600 stimulations per session. The PCM will control all stimulation parameters and ensure that stimulation does not exceed the predefined daily limits.

9. Sample Size of Study

The purpose of this IDE is to gather early safety data for participants implanted with the ReStore device. Given the small sample size of 20 participants, there is no statistical rationale.

10. Protocol Deviations and Serious Adverse Events/Adverse Events

Summary: All protocol deviations will be recorded in the participant's chart as they occur, and subsequently formally reported to the IRB at continuing review. Non-serious AEs will be reported to the IRB at continuing review. Serious Adverse Events (SAEs) and Adverse Events (AEs) will be reported to the PI in a timely manner upon identification via email or in person. All SAEs will be promptly reported to the IRB, DSMC, and FDA as appropriate and within 10 business days.

Definition of Adverse Events (AE)

Adverse event means any untoward medical occurrence associated with the use of an intervention in humans, whether or not considered intervention-related (21 CFR 312.32 (a)).

All adverse events, regardless of relationship to the device, will be recorded, as applicable, on the case report forms provided. Adverse events that occur during this study will be treated by established standards of care which will protect the life and safety of the participants.

Adverse events will be assessed and documented at the time of the procedure and at all study follow-up visits. The study investigator will provide source documentation as requested by the sponsor to facilitate reporting and Data Safety and Monitoring Committee (DSMC) review of these events.

Severity of Adverse Events (AE)

Mild: The participant is aware of the sign or symptom, but finds it easily tolerated. The event is of little concern to the participant and/or little clinical significance. The event is not expected to have any effect on the participant's overall health or well-being.

Moderate: The participant has discomfort enough to cause interference with or change in usual activities. The event is of some concern to the participant's health or well-being and may require medical intervention and/or close follow-up.

Severe: The adverse event interferes considerably with the participant's usual activities. The event is of definite concern to the participant and/or poses a substantial risk to the participant's health or well-being. The event is likely to require medical intervention and/or close follow-up and may be incapacitating or life threatening. Hospitalization and treatment may be required.

Relationship to Device or Study Procedure
The investigator will evaluate the relationship of the adverse event to the research intervention according to the following definitions. The term "device-related," as it pertains to adverse events, means that the event was or may have been attributable to a device, or that a device was or may have been a factor in an event, including those occurring as a result of malfunction, poor manufacture, inadequate labeling, or improper design.

The term "procedure-related," as it pertains to adverse events, means that the event was or may have been attributable to a procedure, or that a procedure was or may have been a factor in an event.

- **Definite**: The adverse event is clearly related to the investigational agent(s) or research intervention: the adverse event has a temporal relationship to the administration of the investigational agent(s) or research intervention, follows a known pattern of response, or is otherwise logically related to the investigational product, and no alternative cause is present.
- **Probable**: The adverse event is likely related to the investigational agent(s) or intervention: the adverse event has a temporal relationship to the administration of the investigational agent(s) or research intervention, follows a known or suspected pattern of response, or is otherwise logically related to the investigational product, but an alternative cause may be present.
- **Possible**: The adverse event may be related to the investigational agent(s) or intervention: the adverse event has a temporal relationship to the administration of the investigational agent(s) or research intervention, follows a suspected pattern of response, or is otherwise logically related to the investigational product, but an alternative cause is present.
- **Unlikely**: The adverse event is doubtfully related to the investigational agent(s) or intervention: the adverse event has a temporal or other relationship to the administration of the investigational agent(s) or research intervention, but follows no known or suspected pattern of response, and an alternative cause is present.
- **Not Related**: The adverse event is clearly not related to the investigational agent(s) or intervention: the adverse event has no temporal or other relationship to the administration of the investigational agent(s) or research intervention, follows no known or suspected pattern of response, and an alternative cause is present

Classification of Serious Adverse Events (SAE)

Serious: A Serious Adverse Event is an adverse event which:

- Led to death,
- Resulted in life threatening illness or injury,
- Resulted in participant hospitalization or prolongation of existing hospitalization,
- Resulted in participant disability or permanent damage or required intervention to prevent permanent impairment/damage
- Resulted in anomaly/ birth defect

NOTE: the term "life-threatening" refers to an event in which the participant was at risk of death at the time of the event; it does not refer to an event which hypothetically might have caused death if it were more severe.

Serious Adverse events will be classified as either:

- **Serious Device Related Adverse Event (SDRAE):** The adverse event was definitely or probably the direct result of the device's presence in the body or as a result of activating or operating the device. Definitely or probably are defined below. Examples would be serious event triggered by the activation of the IPG or an infection caused by failure of a sterile package.
- **Serious Surgery Related Adverse Event (SSRAE):** Adverse events that are determined to be definitely or probably the direct result of the surgery. Example include excessive bleeding due damage to a blood vessel, infection due to loss of sterile field or an anesthesia related event.
- **Serious Unrelated Adverse Event (SUAE)**: Adverse events that are determined to not be directly related to the device or surgery. Examples would be a stroke resulting from high blood pressure or an injury resulting from a fall while participating in rehabilitation or during normal daily activities.

Adverse Event Assessment

The site study investigators, sponsor physician and DSMC will review all adverse events, device complications, and unintended adverse device effects and take appropriate action as necessary (including study termination, if necessary). An outside VNS expert may be appointed to review adverse events and safety information, and describe and compare these events relative to the typical VNS therapy events associated with epilepsy and depression).

Adverse events will be assessed and documented by the investigator at all study visits. All suspected AEs (serious, severe, mild, and moderate) irrespective of the classification (SDRAE, SSRAE, SUAE) will be recorded and reported to the Sponsor. Each AE will be reported as soon as possible after the investigational center's knowledge of the event. The investigational center will provide source documentation as requested by the sponsor. Adverse events reported during the study will be listed, documenting course, severity, and possible relationship to the surgery and stimulation. All serious adverse events will be documented and promptly reported to The Texas Biomedical Device Center and all applicable regulatory authorities.

Time Period and Frequency for Event Assessment and Follow-Up

The occurrence of any adverse event (AE) may come to the attention of study personnel during study visits and interviews of a study participant presenting for medical care, or upon review by a study monitor. All AEs including local and systemic reactions not meeting the criteria for SAEs will be captured on the appropriate case report form (CRF). Information to be collected includes event description, time of onset, clinician's assessment of severity, relationship to study device and procedure (assessed only by those with the training and authority to make a diagnosis), and time of resolution/stabilization of the event. All AEs occurring while on study must be documented appropriately regardless of relationship to device or intervention. All AEs will be followed to adequate resolution. SAEs will be followed for the duration of the study, SAE resolution, or stabilization as appropriate to the SAE. All adverse events will be reviewed by the DSMC and determination of the safety for endpoint evaluation will be the more serious and relatedness of the reports, but all will be assessed.

Any medical condition that is present at the time that the participant is screened will be considered as baseline and not reported as an AE. However, if the study participant's condition deteriorates at any time during the study, it will be recorded as an AE. Changes in the severity of an AE will be documented to allow an assessment of the duration of the event at each level of severity to be performed. AEs characterized as intermittent require documentation of onset and duration of each episode.

The Data Safety Monitoring Committee will review all data after 5, 10, 15, and 20 participants finish the study if no serious adverse events are noted. The DSMC charter will outline a plan to assess any SAEs in a timely manner. The SAE response plan will discuss the appropriateness of the participant's continuation in the study, mitigation of any potential risks, and definition of the time period for follow-up observation. The DSMC charter will document the criteria for halting the study. The DSMC will be promptly provided with data surrounding any serious device related adverse event or any device removal due to a failure or serious adverse event. The goals will be to identify the cause of the event, and to determine the potential impact on study participants.

If at any point 5 study participants experience a SDRAE, the study will be halted.

Adverse Event Reporting

The person to whom an adverse event is reported, including but not limited to the surgeon, study nurse, physical therapist, or chief medical officer, will record all reportable events on an adverse event form and report the occurrence to the PI. The PI will promptly report all serious adverse events to the IRBs, DSMC and FDA within 10 business days. At each study visit, the investigator will inquire about the occurrence of AEs since the last visit. Events will be followed by the Chief Medical Officer for the Texas Biomedical Device Center for outcome information until resolution or stabilization.

Serious Adverse Event Reporting

TxBDC is responsible for the classification and reporting of adverse events and ongoing safety evaluation of the clinical investigation in line with ISO 14155:2011 and US regulatory requirements.

Investigator will report “to the Sponsor, without unjustified delay, all serious adverse events and device deficiencies that could have led to a serious adverse device effect; this information shall be promptly followed by detailed written reports” [ISO 14155:2011 § 9.8 b]. Device malfunctions and use errors should also be reported without unjustified delay.

Should any SAE (including all device deficiencies) be identified, anticipated or unanticipated, the Investigator will report the event to TxBDC immediately (at least within one business day after first knowledge of the event) via email as follows:

Contact Names:

Dr. Jane Wigginton, CMO	Dr. Rita Hamilton,PI
Texas Biomedical Device Center	Baylor Rehabilitation Institute
(469) 407-7403	(214) 820-8557

Contact Email addresses:

Jane.Wigginton@UTDallas.edu	Rita.hamilton@baylorhealth.edu
Jane.Wigginton@UTSouthwestern.edu	

The Investigator should provide additional information on the SAE by updating the information in the adverse event report forms as updates become available. TxBDC may also ask for additional clinical reports including redacted source documents to be provided by the Investigator to assist in the assessment of the event. Significant new information and updates should continue to be submitted promptly to TxBDC and the Investigator should follow the SAE until it is resolved or no further improvement is expected.

It is the responsibility of each Investigator to promptly report all Serious Adverse Events and/or Serious Adverse Device Effects to the IRB, according to national regulations and IRB requirements, as well as the DSMC and FDA within 10 business days.

Determination and Reporting of Unanticipated Adverse Device Effects

The Sponsor shall review all reported SAEs to evaluate whether they meet the criteria for an Unanticipated Adverse Device Effect (UDAE). For adverse events that are determined to be UADEs, the Sponsor will submit an expedited safety report to the FDA’s Center for Devices and Radiological Health (CDRH). The expedited safety report will be submitted to the FDA as soon as possible and, in no event, later than ten (10) business days after the Sponsor first receives notice of the UADE. A copy of this safety report will be provided to all participating study Investigators.

If, following receipt and investigation of follow-up information regarding an adverse event that was previously determined not to be a UADE, the Sponsor determines that the event does meet the requirements for expedited reporting, the Sponsor will submit a report as soon as possible, but in no event later than ten (10) business days after this is determined.

Reporting Events to Participants

In the event of a SDRAE or SSRAE, the study sponsor will, in writing and in person, provide a description of the event, the potential impact on a study participant if it were to occur with their device, and any potential efforts to mitigate further occurrence. Study participants will be informed within 10 working days once a determination that the SAE was directly related to the study device.

Reporting of Pregnancy

Study participants that have not yet been implanted will not be implanted if they become pregnant. If a study participant becomes pregnant, the Chief Medical Officer will review the risks with the participant, suspend all study interventions, and obtain informed consent to continue to monitor the pregnant participant through the pregnancy. The study sponsor may offer to delay study treatment until after the pregnancy.

11. Methods of Data Analysis

Statistical Hypotheses

The primary objective of this study is to evaluate the safety of the ReStore stimulator system. Safety will be assessed at the completion of the randomized, blinded portion of the study.

The secondary objectives are to provide feasibility and early efficacy information on several assessments, including the International SCI Upper Extremity Basic Data Set, International Standard for Neurological Classification of Spinal Cord Injury (upper extremity) , Graded Redefined Assessment of Strength, Sensibility, and Prehension (GRASSP), International SCI Pain Basic Data Set, Hospital Anxiety and Depression Scale (HADS) , Participant Health Questionnaire 9 (PHQ-9), and quantitative assessments of hand and wrist force and range of motion. Secondary efficacy measures will be assessed at the completion of the randomized, blinded portion of the study, and at the completion of the open-label extension. The primary comparisons are assessment of efficacy across groups at the completion of the randomized, blinded portion of the study, within groups at the completion of the randomized, blinded portion of the study, and within group after the completion of the OLE aligned by the number of therapy sessions.

Populations for Analyses

All summaries will be performed on the Intent-to-Treat (ITT) population, defined as all participants who successfully complete the implant procedure and complete at least one post-implant assessment. In addition to the Intent-to-Treat population, efficacy analyses will be performed on a Per Protocol (PP) population as defined below.

To be included in the Per Protocol population, participants must be considered compliant with treatment and be without major protocol violations that could impact and/or compromise the safety or efficacy of the treatment. Exclusion from the PP population will be finalized prior to database lock in a blinded manner.

Statistical Analyses

For each outcome measure, the means with standard deviations and percentage change in improvement will be reported. Statistical analysis of primary and secondary endpoints are further described below.

Analysis of Secondary Endpoint(s): Standard statistical methods will be employed to analyze all data. It is anticipated that the following techniques may be used: descriptive statistics, paired T-test, Bowker's test repeated measures ANOVA, and graphical displays. Assumptions of normality and homogeneity of variance will be tested with the Shapiro-Wilks test. If the distributional assumptions are violated, non-parametric techniques, such as Wilcoxon's Rank-Sum test and the Kruskal-Wallis test, will be employed.

All data analyses and statistical testing will be conducted using SPSS 24.0 or higher. All statistical tests will use a significance level of $\alpha = 0.05$. All data collected in this study will be reported using data listings, summary tables, and graphical displays. Descriptive statistics will be provided for each variable. The statistics will include sample size, mean, median, standard deviation (SD), minimum, and maximum for continuous variables. Categorical variables will be summarized using frequency and percentages.

All descriptive summaries will be reported by treatment group. When summarizing the long term outcomes, data will be grouped into 1 active treatment group, aligned by like visits. Additionally, data for secondary outcome measures may be combined for participants based on the total amount of active therapy received.

Safety Analysis: The safety endpoints will be analyzed as specified below.

- Adverse events with an onset during the course of study, including during the surgical procedure, will be recorded and tabulated. All adverse events will be tabulated, by body system, by first occurrence of the event, maximum severity, and strongest relationship to study treatment and implant surgery. Results will be summarized by treatment group. Furthermore, any adverse events considered serious and any adverse events resulting in discontinuation of stimulation or explanation of the device will be listed.
- Device complications will be tabulated in a manner similar to the adverse event summaries with an emphasis on any unanticipated or serious unanticipated adverse device effect.

Tabulation of Individual Participant Data: Individual data will be reported for baseline, interim, and final assessments.

12. Potential Risks/Benefits

Adverse events reported during the study will be listed, documenting course, severity, and possible relationship to the study device. All serious adverse events will be documented and reported to Institutional Review Board at Baylor University Medical Center (BUMC), data safety monitoring committee (DSMC), and all applicable regulatory authorities within the appropriate time period.

The Hospital Anxiety and Depression Scale (HADS) will be collected at baseline and end-of-study to assess any changes in depression. Vagus nerve stimulation can reduce depressive episodes in participants with depression; similar to other depression therapies, mania has been reported rarely, especially in those with bipolar disorder.

Physical Risks
Since this is a new device, no experience with device longevity or malfunctions is available beyond the canine study and our bench testing. Although malfunctions are not expected, they are possible during this study. All device malfunctions with the IPG and PCM will be evaluated and documented.

Surgical Risks:

Possible Adverse Events Related to the implantation/explantation procedure:

- Nerve damage
- Side effects from the anesthesia
    - Breathing and heart problems
    - Collapsed lung (pneumothorax)
    - Drug reactions
    - Aspiration
    - Nerve damage
    - Cardiac arrest
    - Brain damage
    - Paralysis
    - Permanent organ damage
    - Memory dysfunction/memory loss
    - Injury to vocal chords, teeth, lips, eyes
    - Awareness during the procedure
    - Death

- Pain caused by the incision
- Blood Clot
- Inflammation (swelling)
- Formation of cysts
- Infection
- Facial numbness
- Local pain after the operation

- Facial paralysis
- Nausea
- Edema
- Paresthesia (sleeping limbs)
- Hematoma (clot in tissue)
- Formation of scar tissue
- Histotoxicological reaction
- Irritation of the skin
- Hoarseness/vocal cord paresis/paralysis (due to surgery)
- Tissue reaction

Stimulation Risks:

Possible Adverse Events Related to the stimulation:

- Diarrhea
- Dyspepsia (indigestion)
- Dysphagia (swallowing problems)
- Dyspnea (problems with breathing)
- Ear ache
- Hoarseness (due to stimulation)
- Hiccup
- Cough
- Laryngospasm
- Muscle twitching during stimulation
- Nausea and vomiting
- Pain (especially in the throat or neck)
- Paresthesia (numbness or tingling sensation of the skin)
- Pharyngitis (infection of the throat)
- Respiratory effects (typically at high output current levels and typically at night during sleep when receiving stimulation)

The 60-fold longer VNS trains used for epilepsy control have been reported to influence heart rate variability; however, clinically relevant cardiac effects have not been observed in controlled studies. Additionally, although not shown to be definitely related to stimulation, a small number of participants in studies of other indications using substantially higher stimulation intensity and duration have reported cardiac abnormalities after stimulation was initiated. Based on a large body of preclinical and clinical literature and our previous clinical investigations using lesser VNS stimulation, cardiac effects are not expected given the substantially lower stimulation intensity and shorter duration employed in the present protocol.

Additional discussion of explantation choices and potential risks:

If adverse events are experienced, the team of surgeons and clinical investigators will determine if the device warrants removal during or at completion of the study. If device removal is warranted, the study team will make a strong recommendation to have the device removed.

Additionally, participants are free to decide to have the device explanted at any time during the study. Upon completion of the study, even if the participant has experience no reported adverse events, it is the participants' choice whether to leave the device implanted or have the device removed.

The risks of leaving the device implanted long-term are currently unknown.

Device removal may have increased risks compared with the original implantation surgery. This is because there may be an increased risk of damage to the structures around the implant because of potential scar tissue from the previous operation. In reported series removal of the implanted electrode (a different device that wrapped wires around the vagus nerve) resulted in a slightly higher incident of nerve injury compared to implantation (4.9 vs 3.6%). As explained in the section on the risks of implantation and explantation, damage to the nerve may result in issues such as hoarseness, difficulty with swallowing and cough.

Participants are encouraged to speak with their doctor to determine whether to leave the device implanted or have it removed.

Participants electing to leave the device implanted may be checked in with annually by phone, and may also be contacted about relevant future studies.

Loss of Confidentiality Risks:
While every effort will be made to protect the confidentiality of all participant information and samples, including locking drawers, locked doors, and password protected, encrypted computers and secure servers, it is possible that that a participant could experience some loss of confidentiality.

Long-Range Risks
The risks of leaving the device implanted long-term are currently unknown. It may be necessary or desirable to remove the implant at some point. The participants would be exposed to the same risks as seen during implantation listed above (such as infection, anesthesia, nerve damage, etc.).

Psychological Risks

Depression will be monitored with the PHQ-9 and anxiety will be monitored with the HADS at screening (Visit 1), baseline (Visit 4) and assessments (Visits 23 and 42). These assessments will be regularly reviewed by clinical personnel involved in the study. If a participant demonstrates significant symptoms (i.e. PHQ-9 total ≥ 20, PHQ-9 item 9 ≥ 1, or HADS total ≥ 11)[21–23], clinical personnel will be notified within 24 hours of the visit. Clinical personnel will make a determination as to further necessary action, including monitoring, referral to a specialist, or

hospital admittance. Any actions will be recorded as an adverse event and processed according to our existing study procedures.

As suicidal ideation/risk must be monitored, the following safety plans will be put into place if a participant endorses suicidality: the assessment will pause, and (a) the interviewer will complete the C-SSRS with the participant and follow the “Suicide Assessment and Management” plan. See attached “Suicide Assessment and Management”, “C-SSRS”, and “Safety Plan” for specific suicide mitigation strategies.” This suicide mitigation strategy is a standardized empirically supported method used in clinical research studies[24,25].

Social Risks
No social risks have been identified by participating in this study.

Legal Risks
No legal risks have been identified by participating in this study.

Economic Risks
No economic risks have been identified by participating in this study.

Rationale for the Necessity of Exposing Human Participants to Such Risks
Spinal cord injury is a devastating disease process that often leads to substantial disability. The development of a therapeutic intervention that may allow these participants to recover lost function could improve their overall general health and wellbeing and at the same time allow them to live independently. While this study is focused on upper limb dysfunction, the same methods could perhaps be used to recover other motor functions including lower limb, respiratory, bowel and bladder issues.

<u>Why the Value of the Information to be Gained Outweighs the Risks Involved:</u>
Targeted Plasticity Therapy takes advantage of the innate ability of the nervous system to rapidly reorganize and repair. The nature of spinal cord injury impairments will provide an opportunity to develop new therapies using this approach. SCI participants tend to be younger and healthier than stroke participants and as a result their participation in the development of new therapies is likely to be safe, providing them the prospect of benefit with lessened risk. Animal data provide a compelling demonstration under multiple conditions that VNS paired with rehabilitation can safely more than double recovery of forelimb strength and significantly increase plasticity compared to rehabilitation alone. Even a portion of this level of improvement in SCI participants could result in a significant improvement in quality of life.

This therapy and the device used to deliver it have been demonstrated safe and effective in a number of animal studies. Moreover, initial clinical studies in chronic stroke participants highlight the potential utility of this therapy to improve recovery of function after spinal cord injury. The surgical procedure might be anticipated to expose the participant to the greatest risk of the study. However, the IPG has been designed to make the surgery as minimally invasive as possible. The stimulator does not have a battery, which mitigates risk and obviates the need for additional

surgeries for battery replacement compared to a traditional device. The MRI testing demonstrates that participants can safely undergo standard clinical MRIs. In summary, the system has been designed with a specific emphasis on mitigating any categories of risk while allowing access to this potentially beneficial therapy.

If Risk is Related to Proposed Procedures Included in Protocol, Any Alternative Procedures that have been Considered and Explanation on Why Alternative Procedures Not Included

There are no known mechanisms for non-invasively activating the vagus nerve in an effective and consistent manner at the prescribed levels necessary for efficacy in the animal models. A number of noninvasive VNS systems have become available, including the CerboMed NEMOS transcutaneous auricular branch vagus nerve (ABVN) stimulator and the electroCore gammaCore transcutaneous neck stimulating device. However, the efficacy of noninvasive stimulation to activate the nerve is highly contested, and the preponderance of evidence in well-controlled, reliable studies points to the failure of these devices to sufficiently activate key brain structures. Studies in animals demonstrate that noninvasive VNS drives significantly weaker effects in a number of studies compared to implanted cervical VNS. Moreover, the largest human study to date using transcutaneous VNS failed to demonstrate significant activation of the locus coeruleus, a key brainstem nucleus in the actions of VNS, compared to sham stimulation. Moreover, studies indicate that stimulation intensities less than 0.4mA or greater than 1.2mA will diminish efficacy, and it is highly unlikely that a transcutaneous system could provide stimulation intensity at a consistent, precise level across sessions and participants. Therefore, it is anticipated that the use of a transcutaneous technique would require very large sample sizes and would be significantly less effective than the current approach. Together, these findings suggest that current noninvasive VNS strategies fail to provide sufficient nerve activation to deliver VNS paired with rehabilitation to improve recovery after SCI.

Known Potential Benefits From Either Clinical or Nonclinical Studies

TxBDC, in conjunction with MicroTransponder Inc., has completed 4 early feasibility studies using TPT in humans (NCT1962558; NCT01253616; NCT01669161; NCT02243020) and is currently participating in the pivotal study of TPT for stroke (NCT03131960). Each of these studies have demonstrated safety and an initial indication of efficacy of TPT for the treatment of tinnitus (De Ridder et al, 2012; Tyler et al., 2016) and recovery of motor function following ischemic stroke (Dawson et al, 2015).

Additionally, a preponderance of evidence in animal models of neurological injury demonstrates that VNS therapy enhances plasticity and recovery. VNS paired with rehabilitation significantly enhances recovery of motor function compared to equivalent rehabilitation without VNS after unilateral SCI, bilateral SCI, ischemic stroke, intracerebral hemorrhage, and traumatic brain injury (Ganzer et al, 2018; Meyers et al, 2018; Hays et al, 2016; Khodaparast et al, 2016; Pruitt et al, 2016; Hays et al, 2014a; Hays et al, 2014b; Khodaparast et al, 2014; Khodaparast et al, 2013).

Immediate Potential Benefits

VNS paired with rehabilitative training has the potential to yield immediate functional benefits for SCI participants. Evidence from prior clinical studies supports the potential for immediate benefits. In the first-in-human tinnitus trial, four of the ten participants that received VNS paired

with tone therapy exhibited clinically meaningful improvements in their tinnitus, both for the affective component, as quantified by the Tinnitus Handicap Inventory, and for the sound percept, as quantified by the minimum masking level. These improvements were stable for more than two months after the end of therapy (de Ridder et al, 2012). The therapy was well tolerated, and no participant withdrew from the study due to complications or side-effects. In the follow-up study with 30 participants, the device was used on 96% of days with good compliance (Tyler et al., 2016). After 6 weeks, the paired VNS group improved on the Tinnitus Handicap Inventory ($p = 0.0012$).

In stroke participants, VNS paired with rehabilitation resulted in a significant improvement in change in Fugl–Meyer Assessment-Upper Extremity score compared to equivalent rehabilitation without VNS (between-group difference, 6.5 points; 95% confidence interval, 0.4 to 12.6). The therapy was well tolerated, there were no serious adverse device effects. One participant went on to participate in a trial therapy in which VNS was paired with tactile stimulation to target recovery of somatosensory function. A case study resulting from these data indicates that VNS therapy may also substantially improve somatosensory thresholds, proprioception, and stereognosis (Kilgard et al, 2018).

Together, TxBDC clinical and preclinical studies suggest that participation in this study may result in improved upper limb function.

Long-Range Potential Benefits

Spinal cord injury is a devastating injury in which motor, sensory and autonomic processes are pathologically altered. The development of a therapeutic intervention that allows these participants to recover lost function could improve their overall general health and wellbeing and at the same time allow them to live independently. While this study is focused on upper limb dysfunction, the same methods could potentially be used to recover other motor functions including lower limb, respiratory, bowel and bladder issues. TxBDC is working on methods for treating sensory disorders as well. The heterogeneous nature of spinal cord injury impairments will provide an opportunity to develop new therapies using this approach. SCI participants tend to be younger and healthier than stroke participants and as a result their participation in the development of new therapies may potentially be safer and more effective.

13. Discontinuation/Withdrawal

Discontinuation of Study Intervention

Discontinuation from the study intervention does not mean discontinuation from the study, follow-up and remaining study assessments will be completed as the participant allows as indicated by the study protocol. If a clinically significant finding is identified (including, but not limited to changes from baseline) after enrollment, the investigator or qualified designee will determine if any change in participant management is needed. Any new clinically relevant finding will be reported as an adverse event (AE).

Potential reasons for discontinuation of intervention (VNS) include increased pain, spasticity, light headedness or some other unacceptable physiological response as a direct result of vagus nerve stimulation that is not controlled by reducing the current, duration of stimulation, pulse width, time

between stimulations or some other parameter. If this occurs, study investigators will wait one week and attempt the intervention again. If study investigators are still unable to stimulate the vagus nerve without an unacceptable physiological response the physician will wait 4 weeks and attempt to restart the intervention. If after the 1 or 4 week waiting period the participant is able to continue the intervention, TxBDC will restart the study at the beginning of the phase they were assigned to. If the unacceptable physiological response continues, the study investigators will work with the participant to discuss options and discuss discontinuing the participant from the study intervention. Study investigators will undertake protocol-specified safety follow-up procedures to capture adverse events (AE), serious adverse events (SAE), and unanticipated adverse device effects (UADEs) if participants discontinue or withdrawal from the study.

The data to be collected at the time of study intervention discontinuation will include the following:

- Stimulation parameters current and voltage that elicited the unacceptable physiological response.
- Date and time
- Number of sessions and stimulations per session
- Physiological response and any quantitative measures of that response.
- Whether or not the participant was removed from the study.
- Time since surgery

Participant Discontinuation/Withdrawal from the Study
Participants are free to withdraw from participation in the study at any time upon request.

An investigator may discontinue or withdraw a participant from the study for the following reasons:

- Pregnancy
- Significant study intervention non-compliance
- If any serious adverse event (SAE) occurs (even if not device related) such that continued participation in the study would not be in the best interest of the participant
- Disease progression which requires discontinuation of the study intervention
- If the participant meets an exclusion criterion (either newly developed or not previously recognized) that precludes further study participation

The reason for participant discontinuation or withdrawal from the study will be recorded on the case report form (CRF). Participants who sign the informed consent form and are randomized but do not receive the study intervention may be replaced.

Participants who sign the informed consent form, receive the study intervention, and subsequently withdraw or are withdrawn or discontinued from the study, can be replaced. All participants who are terminated from the study will have the reason for their termination documented. At the end of the study or if the participant withdraws early, the investigator will discuss device follow-up with the participant to ensure the participant receives appropriate ongoing follow-up according to local standard of care. If an implanted participant withdraws early, the investigator and surgeon will discuss device system removal and schedule a removal surgery date if desired by the participant. For participants who are never implanted or are explanted, they will not be followed

under the typical visit schedule. However, they will be followed as long as necessary to indicate recovery/resolution from any potential complication, at which point they will be discontinued. Follow-up information will be provided on all such participants; they will be designated as non-randomized/failed surgery.

If a participant withdraws from the study prior to completion of 18 active stimulation in-office rehabilitation sessions, an additional participant may be enrolled in their place.

The DSMC will review all adverse events, device complications, and unanticipated adverse device effects and take appropriate action as necessary (including study termination, if necessary). An outside VNS expert may be consulted if there are any non-standard events. This study may be temporarily suspended or prematurely terminated if there is sufficient reasonable cause. Written notification, documenting the reason for study suspension or termination, will be provided by the suspending or terminating party to the Chief Medical Officer, the IRB at Baylor University Medical Center, the IRB at UT Southwestern, the IRB at UT Dallas, and the FDA. If the study is prematurely terminated or suspended, the PI will promptly inform the IRB and will provide the reason(s) for the termination or suspension. Potential reasons include:

- Determination of unexpected, significant, or unacceptable risk to participants
- Demonstration of efficacy that would warrant stopping
- Insufficient compliance to protocol requirements
- Data that are not sufficiently complete and/or evaluable

The study may resume once concerns about safety, protocol compliance, and/ or data quality are addressed and satisfy the sponsor, DSMC, IRBs and/or FDA.

Lost to Follow-Up
A participant will be considered lost to follow-up if he or she fails to return for 6 consecutive scheduled visits and is unable to be contacted by the study site staff.

- The site will attempt to contact the participant and reschedule the missed visit and counsel the participant on the importance of maintaining the assigned visit schedule and ascertain if the participant wishes to and/or should continue in the study.
- Before a participant is deemed lost to follow-up, the investigator or designee will make every effort to regain contact with the participant (where possible, 3 telephone calls, 3 emails and/or text messages, and, if necessary, a certified letter to the participant’s last known mailing address or local equivalent methods). These contact attempts will be documented in the participant’s medical record or study file.
- Should the participant continue to be unreachable, he or she will be considered to have withdrawn from the study with a primary reason of lost to follow-up.
- If a participant is lost to follow-up, they may still contact the study staff to have their device removed for up to 2 years from the date of implantation at no cost to them or their insurance company.
-

14. Regulatory, Ethical, and Study Oversight Considerations

Informed Consent
Only persons who sign the Informed Consent will be allowed to participate in this clinical study. The original signed consent will be retained in each participants study file.

Study Discontinuation and Closure
This study may be temporarily suspended or prematurely terminated if there is sufficient reasonable cause. Written notification, documenting the reason for study suspension or termination, will be provided by the suspending or terminating party to the IDE sponsor and regulatory authorities. If the study is prematurely terminated or suspended, the PI will promptly inform study participants, the IRB, and sponsor and will provide the reason(s) for the termination or suspension. Study participants will be contacted, as applicable, and be informed of changes to study visit schedule.

Circumstances that may warrant termination or suspension include, but are not limited to, determination of unexpected, significant, or unacceptable risk to participants.

Study may resume once concerns about safety, protocol compliance, and data quality are addressed, and satisfy the sponsor, IRB and/or FDA.

Confidentiality and Privacy
Participant confidentiality and privacy is strictly held in trust by the participating investigators, their staff, and the sponsor. Therefore, the study protocol, documentation, data, and all other information generated will be held in strict confidence. No information concerning the study or the data will be released to any unauthorized third party without prior written approval of the sponsor.

All research activities will be conducted in as private a setting as possible.

The study monitor, other authorized representatives of the sponsor, representatives of the Institutional Review Board (IRB), regulatory agencies may inspect all documents and records required to be maintained by the investigator, including but not limited to, medical records (office, clinic, or hospital) and pharmacy records for the participants in this study. The clinical study site will permit access to such records.

The study participant's contact information will be securely stored at each clinical site for internal use during the study. At the end of the study, all records will continue to be kept in a secure location for as long a period as dictated by the reviewing IRB, institutional policies, or sponsor requirements.

Study visits and rehabilitation sessions will be video recorded for training and study purposes, and portions of sessions will be reviewed live or prerecorded at a later date.

Study participant research data, which is for purposes of statistical analysis and scientific reporting, will be transmitted to and stored at the Texas Biomedical Device Center. Individual participants

and their research data will be identified by a unique study identification number. The study study will use paper based records used by clinical sites and by the Texas Biomedical Device Center research staff. Records will be locked in secured file cabinets. At the end of the study, all study databases and records will be de-identified and archived at the Texas Biomedical Device Center.

Future Use of Data

Data collected for this study will be analyzed and stored at the Texas Biomedical Device Center. After the study is completed, the de-identified, archived data will be stored at the Texas Biomedical Device Center for use by other researchers including those outside of the study. Permission to transmit data will be included in the informed consent.

Key Roles and Study Governance

A contact sheet will provide the names and contact information of the principle investigator, surgeon, and TxBDC Chief Medical Officer.

Data and Safety Monitoring

Safety oversight will be under the direction of a Data and Safety Monitoring Committee (DSMC) composed of three individuals with the appropriate expertise including Spinal Cord Injury, biomedical statistics, and Neurology. Members of the DSMC are independent from the study conduct and free of conflict of interest. The DSMC will meet at least semiannually to assess safety of the study. The DSMC will operate under the rules of an approved charter that will be written and reviewed at the organizational meeting of the DSMC. At this time, each data element that the DSMC needs to assess will be clearly defined. The DSMC will provide its input to the study sponsor.

Clinical Monitoring

Clinical site monitoring may be conducted to ensure that the rights and well-being of trial participants are protected, that the reported trial data are accurate, complete, and verifiable, and that the conduct of the trial is in compliance with the currently approved protocol/amendment(s), with International Conference on Harmonization Good Clinical Practice (ICH GCP), and with applicable regulatory requirement(s).

Clinical Monitor(s) assigned to the study will fulfill all required responsibilities. Monitors will be responsible for maintaining the device accountability log and participant Case Report Forms and assuring that the Investigational Plan has been approved by the appropriate persons and is adhered to. Regular clinical monitoring visits will be conducted by The Texas Biomedical Device Center personnel or by a CRO. A Data Safety Monitoring Committee (DSMC) will be convened, likely comprised of a biostatistician, a PM&R physician, and a neurologist or neurosurgeon.

To ensure that investigators and their staff understand and accept their defined responsibilities, the Clinical Monitor will maintain regular correspondence and perform periodic site visits during the course of the study to verify the continued acceptability of the facilities, compliance with the Investigational Plan, complete documentation and reporting of any adverse events and unanticipated adverse device effects, and the maintenance of complete records.

Clinical monitoring will include review of the case report forms and resolution of missing or inconsistent results and source document checks (i.e., comparison of submitted study results to original reports) to assure the accuracy of the reported data. The Clinical Monitor will evaluate and summarize the results of each site visit in written reports, identifying any repeated data problems with any investigator and specifying recommendations for resolution of noted deficiencies. As required, the conduct and monitoring of the clinical investigation will be in accordance with The Texas Biomedical Device Center's internal procedures. This includes obtaining and maintaining all required investigator and Ethics Committee documentation, site visits and monitoring, control of device shipment and disposition, review and maintenance of case report forms and investigational files, compliance with reporting requirements and monitoring of the investigators' adherence to the protocol. Standardized Case Report Forms will be provided for use at the investigational sites. Investigators are responsible for completion and timely submission of the data to The Texas Biomedical Device Center for data processing.

All hard copy forms and data files will be secured to ensure confidentiality. Investigators will maintain all source documents as required by the protocol, including laboratory results, case report forms, supporting medical records, Informed Consent forms and applicable files. The source documents will be used at the regular monitoring visits to verify information submitted on the Case Report Forms.

Quality Assurance and Quality Control

The Texas Biomedical Device Center will perform internal quality management of the study conduct, documentation and completion. An individualized quality management plan will be developed to describe the site's quality management. Any missing data or data anomalies will be communicated to the PI for clarification/resolution.

Following written Standard Operating Procedures (SOPs), the monitors will verify that the clinical trial is conducted and data are generated and biological specimens are collected, documented (recorded), and reported in compliance with the protocol, International Conference on Harmonization Good Clinical Practice (ICH GCP), and applicable regulatory requirements (e.g., Good Laboratory Practices (GLP), Good Manufacturing Practices (GMP)).

The investigational site will provide direct access to all trial related sites, source data/documents, and reports for the purpose of monitoring and auditing by the sponsor, and inspection by local and regulatory authorities.

Data Handling and Record Keeping

Data Collection and Management Responsibilities:

All source documents will be completed in a neat, legible manner to ensure accurate interpretation of data. Hardcopies of the study visit worksheets will be provided for use as source document worksheets for recording data for each participant enrolled in the study. All electronic records will be stored in a password protected shared drive and only study personnel will have access.

Clinical data (including adverse events (AEs), concomitant medications, and expected adverse reactions data) and clinical laboratory data will be entered into a 21 CFR Part 11-compliant data

capture system. The data system includes password protection and internal quality checks, such as automatic range checks, to identify data that appear inconsistent, incomplete, or inaccurate. Clinical data will be entered directly from the source documents.

Study Records Retention:
Study documents will be retained for a minimum of 2 years, or as required by law.

Protocol Deviations
All protocol deviations will be recorded in the participant's chart as they occur, and subsequently formally reported to the IRB at continuing review.

Publication and Data Sharing Policy
This study will be conducted in accordance with the following publication and data sharing policies and regulations: This study will comply with the NIH Data Sharing Policy and Policy on the Dissemination of NIH-Funded Clinical Trial Information and the Clinical Trials Registration and Results Information Submission rule. As such, this trial will be registered at ClinicalTrials.gov, and results information from this trial will be submitted to ClinicalTrials.gov. In addition, every attempt will be made to publish results in peer-reviewed journals.

Conflict of Interest Policy
The study leadership in conjunction with the University of Texas at Dallas has established policies and procedures for all study group members to disclose all conflicts of interest and has an established mechanism for the management of all reported dualities of interest. All participants in this study comply with UT Dallas' conflict of interest program.

15. Strategies for Recruitment and Retention

- TxBDC will target enrolling 20 spinal cord injured individuals.
- TxBDC anticipates enrolling 20% female, 20% non-white, based on epidemiology and local population.
- TxBDC will carefully select study participants to maximize retention, and will work to make the study a pleasant experience for them.
- No special vulnerable populations will be included in the present study.
- There will only be sites in the United States.
- Study availability will be distributed by word of mouth and fliers at Baylor Rehabilitation Institute, partnering rehabilitation centers, and via traditional and social media.
- Non-coercive payment incentives may be given to encourage adherence to the study protocol. These funds are intended to help cover the travel costs associated with participating in the study.

Fliers will be approved by the IRB and posted in BSWIR patient facing areas. In addition, members of the study team will attend team meetings to meet with clinicians and physicians and inform them about the purpose of the study. Further, link to the trial website will be provided on the BSWIR website.

Members of the research team, clinicians, and physicians may discuss the study with appropriate patients and distribute fliers. A waiting list will also be created for individuals that are interested in participation. Those patients that are not yet 12 months or more since their injury will be asked if they would like a study staff member to contact them about the study once they are eligible. These contacts will be saved as part of the REDCap database.

16. Participant Study Compensation

Participants enrolled will be compensated up to $3,500 to be distributed as described below. Starting with study visit 4 and continuing through visit 42, participants will be compensated a total of $87.50 per visit for their time and inconvenience. Participants will be provided an additional $87.50 in compensation for completing Assessment 2 at the end of Phase 1. Total compensation available is therefore $1,750.00 for Phase 1, $87.50 for completion of Phase 1, and $1,662.50 for Phase 2. Compensation will be credited to the card upon completion of each scheduled study visit. If participants do not complete all study activities, they will still receive compensation for the activities they have completed up to discontinuation.

17. Administrative Responsibility

The principal investigator will be responsible for the conduct of this study and the safety of the participants.

18. References

Agnew WF, McCreery DB, Yuen TG, Bullara LA. Histologic and physiologic evaluation of electrically stimulated peripheral nerve: considerations for the selection of parameters. *Ann Biomed Eng*. 1989;17(1):39-60. doi:10.1007/bf02364272

Biering-Sørensen F, Bryden A, Curt A, et al. International spinal cord injury upper extremity basic data set. *Spinal Cord*. 2014;52(9):652-657. doi:10.1038/sc.2014.87

Catz A, Itzkovich M, Tesio L, et al. A multicenter international study on the Spinal Cord Independence Measure, version III: Rasch psychometric validation. *Spinal Cord*. 2007;45(4):275-291. doi:10.1038/sj.sc.3101960

Corson K, Gerrity MS, Dobscha SK. Screening for depression and suicidality in a VA primary care setting: 2 items are better than 1 item. *Am J Manag Care*. 2004;10(11 Pt 2):839-845.

Dawson J, Pierce D, Dixit A, et al. Safety, Feasibility, and Efficacy of Vagus Nerve Stimulation Paired With Upper-Limb Rehabilitation After Ischemic Stroke. *Stroke*. 2016;47(1):143-150. doi:10.1161/STROKEAHA.115.010477

De Ridder D, Vanneste S, Engineer ND, Kilgard MP. Safety and Efficacy of Vagus Nerve Stimulation Paired With Tones for the Treatment of Tinnitus: A Case Series. *Neuromodulation: Technology at the Neural Interface*. 2014;17(2):170-179. doi:10.1111/ner.12127

Engineer ND, Riley JR, Seale JD, et al. Reversing pathological neural activity using targeted plasticity. *Nature*. 2011;470(7332):101-104. doi:10.1038/nature09656

Foa EB, McLean CP, Zang Y, et al. Effect of Prolonged Exposure Therapy Delivered Over 2 Weeks vs 8 Weeks vs Present-Centered Therapy on PTSD Symptom Severity in Military Personnel: A Randomized Clinical Trial. *JAMA*. 2018;319(4):354-364. doi:10.1001/jama.2017.21242

Ganzer PD, Darrow MJ, Meyers EC, et al. Closed-loop neuromodulation restores network connectivity and motor control after spinal cord injury. *Elife*. 2018;7. doi:10.7554/eLife.32058

Hays SA, Rennaker RL, Kilgard MP. Targeting plasticity with vagus nerve stimulation to treat neurological disease. *Prog Brain Res*. 2013;207:275-299. doi:10.1016/B978-0-444-63327-9.00010-2

Hays SA, Khodaparast N, Hulsey DR, et al. Vagus nerve stimulation during rehabilitative training improves functional recovery after intracerebral hemorrhage. *Stroke*. 2014a;45(10):3097-3100. doi:10.1161/STROKEAHA.114.006654

Hays SA, Khodaparast N, Ruiz A, et al. The timing and amount of vagus nerve stimulation during rehabilitative training affect poststroke recovery of forelimb strength. *Neuroreport*. 2014b;25(9):676-682. doi:10.1097/WNR.0000000000000154

Hays SA, Ruiz A, Bethea T, et al. Vagus nerve stimulation during rehabilitative training enhances recovery of forelimb function after ischemic stroke in aged rats. *Neurobiol Aging*. 2016;43:111-118. doi:10.1016/j.neurobiolaging.2016.03.030

Hulsey DR, Hays SA, Khodaparast N, et al. Reorganization of Motor Cortex by Vagus Nerve Stimulation Requires Cholinergic Innervation. *Brain Stimul*. 2016;9(2):174-181. doi:10.1016/j.brs.2015.12.007

Hulsey DR, Riley JR, Loerwald KW, Rennaker RL, Kilgard MP, Hays SA. Parametric characterization of neural activity in the locus coeruleus in response to vagus nerve stimulation. *Exp Neurol*. 2017;289:21-30. doi:10.1016/j.expneurol.2016.12.005

Itzkovich M, Gelernter I, Biering-Sorensen F, et al. The Spinal Cord Independence Measure (SCIM) version III: reliability and validity in a multi-center international study. *Disabil Rehabil*. 2007;29(24):1926-1933. doi:10.1080/09638280601046302

Ivanova J, G Birnbaum H, Chen L, et al. Cost of Post-Traumatic Stress Disorder vs Major Depressive Disorder Among Patients Covered by Medicaid or Private Insurance. *The American journal of managed care*. 2011;17:e314-23.

Jebsen RH, Taylor N, Trieschmann RB, Trotter MJ, Howard LA. An objective and standardized test of hand function. *Arch Phys Med Rehabil*. 1969;50(6):311-319.

Kahlow H, Olivecrona M. Complications of vagal nerve stimulation for drug-resistant epilepsy: a single center longitudinal study of 143 patients. *Seizure*. 2013;22(10):827-833. doi:10.1016/j.seizure.2013.06.011

Kalsi-Ryan S, Beaton D, Curt A, et al. The Graded Redefined Assessment of Strength Sensibility and Prehension: reliability and validity. *J Neurotrauma*. 2012;29(5):905-914. doi:10.1089/neu.2010.1504

Khodaparast N, Hays SA, Sloan AM, et al. Vagus nerve stimulation during rehabilitative training improves forelimb strength following ischemic stroke. *Neurobiol Dis*. 2013;60:80-88. doi:10.1016/j.nbd.2013.08.002

Khodaparast N, Hays SA, Sloan AM, et al. Vagus nerve stimulation delivered during motor rehabilitation improves recovery in a rat model of stroke. *Neurorehabil Neural Repair*. 2014;28(7):698-706. doi:10.1177/1545968314521006

Khodaparast N, Kilgard MP, Casavant R, et al. Vagus Nerve Stimulation During Rehabilitative Training Improves Forelimb Recovery After Chronic Ischemic Stroke in Rats. *Neurorehabil Neural Repair*. 2016;30(7):676-684. doi:10.1177/1545968315616494

Kilgard M, Rennaker R, Alexander J, Dawson J. Vagus nerve stimulation paired with tactile training improved sensory function in a chronic stroke patient. *NeuroRehabilitation*. 2018;42:159-165. doi:10.3233/NRE-172273

Kirshblum SC, Burns SP, Biering-Sorensen F, et al. International standards for neurological classification of spinal cord injury (revised 2011). *J Spinal Cord Med*. 2011;34(6):535-546. doi:10.1179/204577211X13207446293695

Kroenke K, Spitzer RL, Williams JB. The PHQ-9: validity of a brief depression severity measure. *J Gen Intern Med*. 2001;16(9):606-613. doi:10.1046/j.1525-1497.2001.016009606.x

Marino RJ, Sinko R, Bryden A, et al. Comparison of Responsiveness and Minimal Clinically Important Difference of the Capabilities of Upper Extremity Test (CUE-T) and the Graded Redefined Assessment of Strength, Sensibility and Prehension (GRASSP). *Top Spinal Cord Inj Rehabil*. 2018;24(3):227-238. doi:10.1310/sci2403-227

Meyers EC, Solorzano BR, James J, et al. Vagus Nerve Stimulation Enhances Stable Plasticity and Generalization of Stroke Recovery. *Stroke*. 2018;49(3):710-717. doi:10.1161/STROKEAHA.117.019202

Norman GR, Sloan JA, Wyrwich KW. Interpretation of changes in health-related quality of life: the remarkable universality of half a standard deviation. *Med Care*. 2003;41(5):582-592. doi:10.1097/01.MLR.0000062554.74615.4C

Pruitt DT, Schmid AN, Kim LJ, et al. Vagus Nerve Stimulation Delivered with Motor Training Enhances Recovery of Function after Traumatic Brain Injury. *J Neurotrauma*. 2016;33(9):871-879. doi:10.1089/neu.2015.3972

Stefanovics EA, Rosenheck RA, Jones KM, Huang G, Krystal JH. Minimal Clinically Important Differences (MCID) in Assessing Outcomes of Post-Traumatic Stress Disorder. *Psychiatr Q*. 2018;89(1):141-155. doi:10.1007/s11126-017-9522-y

Tyler R, Cacace A, Stocking C, et al. Vagus Nerve Stimulation Paired with Tones for the Treatment of Tinnitus: A Prospective Randomized Double-blind Controlled Pilot Study in Humans. *Scientific Reports*. 2017;7. doi:10.1038/s41598-017-12178-w

Widerström-Noga E, Biering-Sørensen F, Bryce T, et al. The international spinal cord injury pain basic data set. *Spinal Cord*. 2008;46(12):818-823. doi:10.1038/sc.2008.64

Zigmond AS, Snaith RP. The hospital anxiety and depression scale. *Acta Psychiatr Scand*. 1983;67(6):361-370. doi:10.1111/j.1600-0447.1983.tb09716.x

19. Revision History

Revision	Description	Author	Date
A	ECN 1025 Initial release	Ruchi Verma	2/12/2019
B	ECN 1059 was updated to align with the changes to CPT-SCI-FDA-001 and to be formatted based on Baylor IRB's format.	Amy Porter	10/25/2019
C	ECN 1062 All documents were updated as part of the IRB Submission. Refer to Redline document	Kavita Raman	11/14/2019
D	ECN 1066 All documents were updated as part of the IRB Submission. Refer to redline document	Kavita Raman	11/19/2019
E	ECN 1076 updated general aims and background section	Amy Porter	2/24/2020
F	ECN 1077 Updated primary end points, added case report forms, clarified explant risks	Amy Porter	2/24/2020
F.1 (Am 2-Am 3)	Added recruitment information, updated "male or female" to "adult" and updated BUMC address, updated SoA to include new CRFs and expand general CRFs to all visits<br>NOTE: Reviewed by IRBs but not released via ECN	Amy Porter	9/30/2020
G	ECN 1132. Rev F.1 (Am2 – Am3) was reviewed and approved by participating IRBs and FDA. Rev G is now being released via change control process. No changes made between F.1 and Rev G	Amy Porter	02/01/2021
F.1 (Am 5)	Updated the Schedule of Activities table to include the Reimbursement log under general case report forms. Updated Lost	Amy Porter	12/11/2020

	to Follow-Up section in protocol (pg 33) to specify what other contacts will be made besides telephone calls and a final letter.<br><br>NOTE: Reviewed by IRBs but not released via ECN		
H	ECN 1133 Rev F.1 (Am 5 only) was reviewed and approved by participating IRBS and FDA. Rev H is now being released via change control process. No changes made between F.1 (Am 5) and Rev H.<br><br>NOTE: All changes, namely, F.1 (Am2-Am3), G, F.1 (Am 5) and H were consolidated via this ECN.	Amy Porter	02/12/2021
H.1 (Am6)	Consolidate SoA such that Visit 2 and 3 occur back-to-back at a single visit during trial week 4 instead of as two separate visits. Remove reference to Vital Signs and Symptoms at Stimulation CRF-SCI-042 from footnote in protocol SoA as form is not utilized in trial.	Amy Porter	5/24/2021
H.2	Updated to remove Hand Measures CRF (CRF-SCI-007) from SoA and SoA footnotes	Amy Porter	6/23/2021
I	ECN 1180 - Rev H.1 (Am 6) and Rev H.2 were reviewed and approved by participating IRBS and FDA. Rev I is now being released via change control process. No changes made between RevH.1(Am 6)-Rev H.2 and Rev I.	Amy Porter	08/05/2021
I.1	Added section on psychological risks to discuss procedures for substantial deviations in HADS and PHQ-9 results. Added Attachment A: “Suicide Risk Assessment and Management” and C-SSRS. Updated SoA footnotes to accommodate consolidation of CRF 20 and 25. Revised long term follow-up language to clarify timeline and potential for stim under follow-on studies. Include discussion on by computer-based rehab tools sending automated VNS trigger requests. Minor formatting updates to adjust page breaks.	Amy Porter	10/22/2021
J	ECN 1215 - Rev I.1 (Am 7) was reviewed and approved by participating IRBs and FDA. Rev J is now being released via change control process. No changes made between I.1 and Rev J.	Amy Porter	03/02/2022
J.1	Administrative correction clarifying that sending of trigger requests is not limited to therapists. Some triggering occurs during study visits that do not involve rehabilitation, and therapist triggering is not applicable or feasible in all situations. The changes clarify that other qualified study team personnel can oversee triggering in these circumstances.	Amy Porter	9/28/2022
J.2	Updated Section 12 risks to itemize risks associated with anesthesia.	Amy Porter	1/18/2023
K.0	Removed Kiara Leonard, Emma Turner, and UT Southwestern from coversheet to align with team updates. Updated follow-on study language to clarify that participants may participate in more than one additional study. Removed DoD/HRPO from reporting for non-compliance/unanticipated problems, adverse events, audits, and investigation reports as they are no longer funding this study.	Amy Porter	06/03/2023

	Note that protocol Amendment 10 was skipped to remain in alignment with the consent form.		
K.1	Added “previously” to first sentence of protocol to retain transparency regarding the original trial funding source following completion of the DARPA funded effort on the project.	Amy Porter	07/01/2024

BAYLOR UNIVERSITY MEDICAL CENTER (BUMC)
BAYLOR SCOTT & WHITE INSTITUTE FOR REHABILITATION
&
DIVISION OF TRAUMA
DALLAS, TEXAS

TEXAS BIOMEDICAL DEVICE CENTER
UNIVERSITY OF TEXAS AT DALLAS
RICHARDSON, TEXAS

THE UNIVERSITY OF TEXAS SOUTHWESTERN MEDICAL CENTER
DALLAS, TEXAS

CONSENT FORM AND PRIVACY AUTHORIZATION

PROJECT TITLE: Targeted Plasticity Therapy for Upper Limb Rehabilitation in Spinal Cord Injuries

National Clinical Trial # NCT04288245

PRINCIPAL INVESTIGATOR ("PI"): Rita Hamilton, DO

TELEPHONE NUMBER: 214-820-8557

The information in this section is intended to be an introduction to the study only. Complete details of the study are listed in the sections below. If you are considering taking part in the study, the entire document should be discussed with you before you make your final decision. You can ask questions about the study now and at any time in the future. Taking part in this study is voluntary.

<u>Key Information -</u>

1. WHY HAVE I BEEN ASKED TO TAKE PART IN THIS STUDY?

You are being asked to take part in this research study because you have a diagnosis of spinal cord injury with upper limb deficits (i.e., limitations to upper body movements).

2. WHY IS THIS STUDY BEING DONE AND HOW LONG WILL IT LAST?

The purpose of this study is to test the safety of using the ReStore System to deliver vagus nerve stimulation (VNS) during physical therapy to the upper limbs of subjects with Spinal Cord Injury with upper limb deficits and see what effects (good and bad) it has on you and others with Spinal Cord Injuries. The ReStore System consists of a very small vagus nerve stimulator that sits on the vagus nerve in the neck and two devices used to trigger the vagus nerve stimulation. We think that you will be in the study for about 20 weeks; long-term assessment of safety will be performed twice yearly for up to 2 years after your implant date when possible.

3. WHAT WILL I BE ASKED TO DO IN THIS STUDY?

If you decide to take part in this study and are eligible, you will be asked to attend about 20 study visits. Following about a 6-week screening, consent, surgery to place the vagus nerve stimulator on the vagus nerve in the neck, and recovery period, you will receive 6 weeks of active VNS or placebo stimulation during upper limb physical therapy. You will be assessed at the end of those 6 weeks; at which time you may elect to exit the study or begin an additional 6 weeks of active VNS paired with rehabilitation.

4. WHY MIGHT I WANT TO TAKE PART IN THIS STUDY?

If you agree to take part in this study, there may or may not be direct medical benefit to you. We hope that the information learned from this study will benefit other subjects with this diagnosis in the future.

5. WHY MIGHT I NOT WANT TO TAKE PART IN THIS STUDY?

You may decide that you do not want to take part because you have a 50/50 chance of being in the placebo group. Both groups will receive identical assessments, procedures, and ReStore System equipment. However, during Phase 1 of the study, only subjects in the active group will receive VNS therapy during rehabilitation activities. Subjects in the placebo group will receive rehabilitation therapy with placebo stimulation. Following this first phase of the study, all study subjects will receive active VNS therapy with upper limb physical therapy. There is no guarantee that the ReStore system and upper limb Physical Therapy will help with your Spinal Cord Injury injury/damage. In addition, you may have side effects while on the study. The most common side effects that people experience include pain after surgery and sensations during vagus nerve stimulation. The most serious side effects (experienced by about 1%) include nerve damage, side effects from anesthesia, numbness/tingling, irritation of the skin or tissue from the surgery or the device and breathing difficulty during stimulation. The researchers do not know all of the side effects that could happen. For a complete description of known risks, refer to the Detailed Information section of the consent form. The possible benefits of taking part in this study are the same as receiving upper limb physical therapy without being in this study.

6. WHAT OTHER OPTIONS ARE THERE?

There are other options available to you. Your other choices may include:

1. Receiving no treatment at this time for SCI
2. Receiving upper limb Physical Therapy without vagus nerve stimulation
3. Participating in a different clinical study

Please talk to your regular doctor about these and other options.

7. HOW WILL TAKING PART IN THE STUDY AFFECT ME FINANCIALLY?

(1) There is no additional cost to you if you take part in this study. Although we do not provide additional funds for parking and transportation, if you do need transportation, we will provide you with a list of options and may be able to cover some or all of these costs.
(2) You will receive up to $3,500.00 for the time you spend in this study. You will receive $87.50 for each visit after implantation of the device. Payment will be made in the form of a gift card provided by UT Dallas, which can be used as a credit or debit card. The money will be credited to the card upon the completion of each scheduled study visit.

IRB NUMBER: 019-356
IRB APPROVAL DATE: 07/02/2024
IRB EXPIRATION DATE: 12/6/2024

Detailed Information Section

What is the Status of the Device Involved in This Study?

The ReStore System is an investigational device, which is not approved by the US Food and Drug Administration. The placebo group will be implanted with the same device (ReStore system); the only difference is that they will not be receiving active stimulation during therapy.

How Many People Will Take Part in This Study?

About 20 people will take part at this location.

What Will I Be Asked to Do?

Screening- After you sign this consent to participate, exams, tests, and/or procedures may be done as described below to find out if you can continue in the study; this is called screening. We may also be able to use the results of exams, tests, and/or procedures you completed before enrolling in this study. Many of the procedures and visits that are described below as **"standard care"** would be done even if you do not take part in this research study. You will be told which ones are for **"research only."** These will take place in an outpatient setting. We will complete some or all of the following in preparation for your surgery.

Pre-operative Tests and Preparation:
Physical Exam- including measurement of your height, weight, listen to your heart, your pulse, and blood pressure
Blood draw- Blood (about two teaspoons) will be taken from a vein (or artery) in your arm to count the number of red blood cells and white blood cells, to measure the amount of sugar/cholesterol in your blood, and to determine your overall, general health
Pregnancy Test- If you are capable of becoming pregnant, a pregnancy test will also be done before you receive study treatment. More specifically, a blood pregnancy test (about half of a teaspoon) will be done, as it is more sensitive than urine.

This visit will take about 1 hour. The results of the screening exams, tests, and/or procedures will be reviewed to determine whether you will be allowed to continue in the study. If you are not allowed to continue in the study, the researcher will discuss the reasons with you. This will take place in an outpatient setting.

Study Procedures- as a subject, you will undergo the following procedures:
The following assessments may be completed by a research team member:

Brief physical exam and medical history: This will be completed by a medical professional on the research team and is meant to assess your overall health and ability to take part in the study. This will take 15 minutes.
Laryngoscopy: This will be completed by a doctor on the team, and is a very safe, quick, simple, and routine in-office procedure where your throat will be numbed with a spray, and then a very small tube-shaped camera (endoscope) will be inserted down to the voice box (larynx) through the mouth. The camera will allow the doctor to see the inside of

Full Accreditation
IRB NUMBER: 019-356
IRB APPROVAL DATE: 07/02/2024
IRB EXPIRATION DATE: 12/6/2024

your voice box, including the vocal cord positions and movement. This is important, because some people have one vocal cord that does not move, but they are unaware of it. A rare but important risk of vagus nerve implantation is nerve damage that affects vocal cord motion. Therefore, as the device will be implanted on your left side, if your right vocal cord does not move correctly, you would be excluded from the study to protect your safety. If both vocal cords move well, or if your left vocal cord does not move well but your right one does, you can be included in the study. This will take about 15 minutes.

Clinical Assessments and Examinations:

- International SCI Upper Extremity Basic Data Set: used to collect basic information on upper limb function and surgical history (~10-20 min)
- International Standard for Neurological Classification of Spinal Cord Injury (upper limb) AIS/ASIA: used to evaluate motor and sensory function, and neurological levels (~10 min-1 hour)
- Jebsen-Taylor Hand Function Test: used to measure hand functions commonly used in daily activities (~15 min)
- Graded Redefined Assessment of Strength, Sensibility, and Prehension (GRASSP): series of questions used to measure clinical upper limb disability (~45 min)
- Spinal Cord Injury Independence Measure (SCIM) III: used to measure level of independence in everyday functions (~30-45 min)
- International SCI Pain Basic Data Set: used to report pain levels (~10 min)
- Hospital Anxiety and Depression Scale (HADS): used to measure levels of depression and anxiety in a healthcare setting (~2-5 min)
- Subject Health Questionnaire 9 (PHQ-9): used to measure depression (~2-5 min)
- Quantitative Force and Range of Motion Assessment: used to measure physical functions (~10 min-1 hour)

Demographic information: We will ask for information such as age, sex, and ethnic origin. This will take about 5 minutes.

Survey of devices: This may be administered at each active therapy session. A study staff member will record information from the app on the study phone.

During the initial visit, you will be assessed for the inclusion and exclusion criteria as well as medical history and physical exam. If you meet the criteria and give consent, you will be enrolled in the study.

When you are determined to be medically eligible for the study, and it is determined that your spinal cord injury (SCI) meets the study criteria and your eligibility for surgery is verified (which includes physical exams, plus a pregnancy test if you are a woman of childbearing age), the study team will schedule your surgery for the implantation of the ReStore System (i.e., Vagus Nerve Stimulator).

The surgery is expected to take about 60 minutes and involves one incision on the left side of your neck. The surgery will take place in the operating room in outpatient surgery. After you receive anesthesia and are asleep, the surgeon will locate the vagus nerve on the left side of your

IRB NUMBER: 019-356
IRB APPROVAL DATE: 07/02/2024
IRB EXPIRATION DATE: 12/6/2024

neck and then will place the stimulator next to the vagus nerve. It will be held in place by a silicone cuff. Your incisions will be closed with stitches. After surgery you will recover for about one week or as directed by your doctor. Once you have recovered, you will start your study treatment.

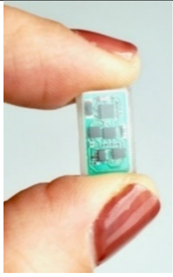	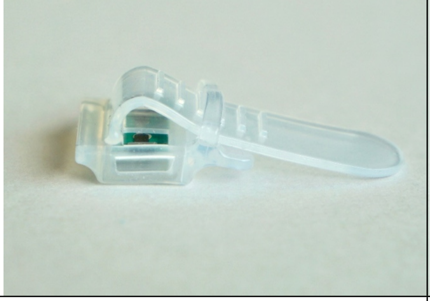	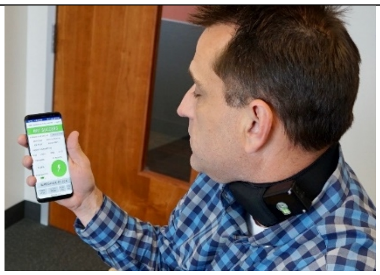
1) Vagus Nerve Stimulation Device (VNS)	2) VNS Device placed in the cuff	3) Smart device and the Power and Communication Device (PCM) in the neckband.
**Fig. 1. System Components**		

The ReStore System, shown in Figure 1, consists of: 1) the vagus nerve stimulation device 2) silicone rubber nerve cuff to hold the device in place, 3) the Power and Communication Device (PCM) and study phone (similar to a cell phone). The VNS device is placed in the silicone rubber cuff and implanted on the left cervical branch of the vagus nerve. The Power and Communication Device is worn externally in a neckband and powers, communicates with, and controls the VNS device. A secure smart device programs and controls the VNS device. During in-office physical therapy, study personnel press an icon on the smart device to trigger stimulation. The secure smart device records session information and stimulation times. During the study, your study team will first test the device and settings in the office. Each of your clinic visits (i.e., outpatient clinic at BUMC) will last about one hour.

You will be randomly selected (like the flip of a coin) to receive either the active VNS or the placebo VNS. Neither you nor your doctor will choose which group you will be in. Following this first phase of the study, all study subjects will have the opportunity to receive active VNS therapy with upper limb physical therapy.

You will have a one in two chance of being in the placebo group. Most subjects in the active stimulation group in previous studies report not sensing the stimulation after the first few trials. You will be instructed that you may initially perceive stimulation, but the perception may fade. Because of this, neither you nor the researchers will know whether you are receiving active stimulation or placebo. In the event of an emergency, there is a way for the researcher to find out which are you receiving.

If you agree to take part in the study, and sign the informed consent document, you will take part in the following Study Phases:

Phase 1: During the first six weeks you will undergo consent, three assessments, surgical implantation, and assignment to one of the groups. You must wait a minimum of two weeks after surgery to start rehabilitation.

Visit 1 (before surgery): Following informed consent, the study doctor will conduct a physical exam, and study personnel will test spinal cord injury impairment.

Visit 2-3 (surgery): You will have a pre-op visit and will be surgically implanted with the study device via surgery. This is generally a minor day surgery that does not require an overnight stay.

Visit 4 (post-op recovery and assessment): About one week following device implantation, you will have more spinal cord injury assessments before beginning treatment. Additionally, the VNS will be activated to gauge tolerability and adjust parameters as needed.

If you are randomized to Treatment Group A, you will receive rehabilitation and active stimulation during weeks 7-12. If you are randomized to Treatment Group B, you will receive rehabilitation and placebo stimulation during weeks 7-12. Both groups will have an assessment in week 13 (within 7 to 14 days of the last session).

Phase 2: Whether you are in Group A or B, Phase 2 will include 18 more sessions of rehabilitation (~6 weeks) where you will receive active VNS stimulation with rehabilitation. An assessment will be performed about 1 week (within 7 to 14 days) after the last session.

Long term Follow Up: Both groups will be offered long-term follow-up. Assessments will be performed twice annually for two years after the date of implant. There is no plan to stimulate during this period.
The schedule of activities below is an estimate and is expected to vary on occasion.

Schedule of Activities (SOA)

	Pre therapy				Phase 1							Phase 2 OLE						
Visit Number	1	2	3	4	5 - 7	8 - 10	11 - 13	14 - 16	17 - 19	20 - 22	23	24 - 26	27 - 29	30 - 32	33 - 35	36 - 38	39 - 41	42
Approximate Week Number	1	4	4	6	7	8	9	10	11	12	13	14	15	16	17	18	19	20
Sign Informed Consent	x																	
Medical History	x																	
Physical Exam	x																	
Laryngoscopy	x																	
Pre-Op Assessment		x																
Implantation			x															
Post-Op and Stimulation Test				x														
Vital Signs	x	x	x	x	x	x	x	x	x	x	x	x	x	x	x	x	x	x
Collect Adverse Events	x	x	x	x	x	x	x	x	x	x	x	x	x	x	x	x	x	x

IRB NUMBER: 019-356
IRB APPROVAL DATE: 07/02/2024
IRB EXPIRATION DATE: 12/6/2024

	Pre therapy				Phase 1							Phase 2 OLE						
Visit Number	1	2	3	4	5 - 7	8 - 10	11 - 13	14 - 16	17 - 19	20 - 22	23	24 - 26	27 - 29	30 - 32	33 - 35	36 - 38	39 - 41	42
Approximate Week Number	1	4	4	6	7	8	9	10	11	12	13	14	15	16	17	18	19	20
Collect Device Setting Information				x	x	x	x	x	x	x	x	x	x	x	x	x	x	x
Complete Case Report Forms	x	x	x	x	x	x	x	x	x	x	x	x	x	x	x	x	x	x
Therapy																		
Rehabilitation					x	x	x	x	x	x		x	x	x	x	x	x	
Vagus Nerve Stimulation (in Active Group)				x	x	x	x	x	x	x		x	x	x	x	x	x	
Assessments																		
International SCI UE Basic Data Set	x										x							x
ASIA Assessment	x										x							x
SCI Independence Measure III	x										x							x
International SCI Pain Basic Data Set	x										x							x
Patient Health Questionnaire 9	x			x							x							x
Hospital Anxiety and Depression Scale	x			x							x							x
GRASSP	x			x							x							x
Jebsen-Taylor Assessment	x			x							x							x
Force and Range of Motion Assessments	x			x							x							x
Participant Satisfaction Survey											x							x

The researchers will discuss your options for medical care when your time in this study ends.

You can decide to have the device removed at any time. The cost of this will be covered within two years of your date of implant. After those two years, you will be responsible for paying the cost of the surgery to remove the device.

Your Responsibilities as a Research Subject:

Commitment: While you always have the right to change your mind and leave this study, you should enter this study only if you think you will want to be in it until it ends.

Visits: You agree to come for all study visits and to follow the instructions of the research doctor/staff, even if you stop the study treatment. In case it is not possible for you to attend a visit, we will contact you by phone or mail.

Problems: You will let the research doctor/staff know immediately if any problems occur while you are involved in this study. You will also let the research doctor/staff know if you have to go to an emergency room, doctor's office, or a hospital.

Medicines: You will let the research doctor/staff know about any changes in your prescription medicines, over-the-counter medicines, and all vitamins or supplements that you take, and will keep all study medicine and/or supplies out of the reach of others.

IRB NUMBER: 019-356
IRB APPROVAL DATE: 07/02/2024
IRB EXPIRATION DATE: 12/6/2024

Individuals of child-bearing potential: You should not become pregnant while taking part in this study because we do not know how the study procedures could affect a fetus. It is important that you talk to your study doctor about avoiding pregnancy during this study. If you think you might have become pregnant while you are in this study, you must tell the study staff right away so that management of the pregnancy and the possibility of stopping you from taking part in this study can be discussed.

***Other studies**:* You will not take part in any other study at the same time you are in this study (unless you are given permission by study lead investigators).

How Long Will I Be in This Study?

You will be in this study for about 20 weeks with the intervention taking place over 12 weeks. It will take about 6 weeks for screening, consent, and surgical placement of the vagus nerve stimulator on the vagus nerve in the neck, and recovery period. You will receive 6 weeks of active VNS or placebo stimulation during upper limb physical therapy. You will be assessed about 1 week after completion of physical therapy. During phase 2, you will enter an open-label extension of 18 more sessions of rehabilitation (~6 weeks) where you will receive active VNS stimulation with rehabilitation. While you are taking part in this study, you will be asked to attend about 42 total visits with the researchers or study staff.

The PI/researcher may decide to take you off this study if any of the following occur:

- He/She feels that it is in your medical best interest.
- Your condition worsens.
- You become ineligible to take part.
- New information becomes available.
- This study is stopped by the sponsor.
- Your condition changes and you need treatment that is not allowed while you are taking part in the study.
- You do not follow instructions from the researchers.

You are free to decide to have the device removed at any time during the study. Upon completion of the study, even if you have not experienced any issues with the device, it is your choice whether to leave the device implanted or have the device removed.

You can stop taking part in this study at any time. However, if you decide to stop taking part in this study, we encourage you to talk to the researcher and your regular doctor first. The researcher may ask you to complete study withdrawal procedures at a final study visit. This visit includes documentation and final therapeutic assessments. There is no risk to you if you do not complete the final withdrawal procedures and you can choose not to participate in them. You may still choose to have the device removed without cost to you or your insurance company for up to two years from implantation date, even if you withdrew from the study and chose to keep the device at the time.

IRB NUMBER: 019-356
IRB APPROVAL DATE: 07/02/2024
IRB EXPIRATION DATE: 12/6/2024

Regardless of if you keep the device or choose to have it removed, we intend to follow-up with you at least once per year for two years following implant. It is very important in clinical trials to know if subjects are alive and well by the end of the clinical trial. To do this, we ask the following things:

- ***Location and telephone number updates:*** At each visit, our staff will ask you if any of your contact information has changed. Please let us know of any changes at any time.
- ***Closest Relative or Friend:*** Please provide us with the name of a relative or friend whom we can contact in the event that you cannot make visits and we cannot reach you. We want to be able to ask this person for information about your health status.
- ***Doctor:*** If we cannot reach you or one of the people you have listed above, we will contact your doctor to find out about your health.
- ***Public Registries:*** If you drop out of this study and we are unable to contact anyone with information about your health status, we will search Public Registries (such as the US Postal Service, Social Security, and social media) for information about you.
- ***Minimum information:*** We will not disclose any of your personally identifiable health information beyond the minimum required to confirm your health status.

What Are the Risks of This Study?

While in this study, you are at risk for these reactions, sometimes bad, which are listed below. You should discuss these with the researcher and/or your regular doctor. There also may be other reactions that we cannot predict. These unknown reactions could also be to your unborn child if you are pregnant or become pregnant while on this study. Other medicines may be given to make them less serious and uncomfortable. Many of these reactions go away shortly after the vagal stimulation is stopped, but in some cases, they can be serious or long lasting and permanent.

Risks and reactions related to surgery for placing/removing the device include:

Likely, some may be Serious
In 100 people, about 21 – 100 may have:

- Pain caused by the incision
- Inflammation (area around the incision site becomes red, swollen, and sometimes painful)
- Local pain after the operation
- Nausea
- Formation of scar tissue
- Irritation of the skin

Less Likely, some may be Serious
In 100 people, about 2 – 20 may have:

- Blood Clot
- Formation of cysts

IRB NUMBER: 019-356
IRB APPROVAL DATE: 07/02/2024
IRB EXPIRATION DATE: 12/6/2024

- Infection
- Facial numbness (losing feeling in your face)
- Facial paralysis (weakness in your face)
- Edema (swelling near the incision site)
- Paresthesia (numbness, tingling)
- Hematoma (solid swelling of clotted blood)
- Hoarseness/vocal cord paresis/paralysis
- Tissue reaction (tissue becomes inflammed i.e., red and swollen)

Rare and Serious
In 100 people, about 1 or fewer may have:
- Nerve damage
- Side effects from the anesthesia
    - Breathing and heart problems
    - Collapsed lung (pneumothorax)
    - Drug reactions
    - Aspiration
    - Nerve damage
    - Cardiac arrest
    - Brain damage
    - Paralysis
    - Permanent organ damage
    - Memory dysfunction/memory loss
    - Injury to vocal chords, teeth, lips, eyes
    - Awareness during the procedure
    - Death
- Parasthesia (tingling, pricking, chilling, burning, or numb sensation on the skin)
- Skin or other tissue having a reaction to the surgery or the device

Risks and side effects related to the Vagus Nerve Stimulation include those which are:

Likely, some may be Serious
In 100 people, about 21 – 100 may have:
- Pain, especially in the throat or neck
- Muscle twitching during stimulation
- Hoarseness
- Pharyngitis (sore throat)
- Cough
- Irritation of the skin

Less Likely, some may be Serious
In 100 people, about 2 – 20 may have:

IRB NUMBER: 019-356
IRB APPROVAL DATE: 07/02/2024
IRB EXPIRATION DATE: 12/6/2024

- Diarrhea
- Dyspepsia (upset stomach)
- Dysphagia (difficulty or discomfort with swallowing)
- Earache
- Hiccup
- Laryngospasm (spasm of the vocal cords)
- Nausea and vomiting

Rare and Serious
In 100 people, about 1 or fewer may have:

- Respiratory effects
- Histotoxicological reaction (reaction to the device, i.e., an allergic reaction to the materials or redness and swelling around the device)
- Dyspnea (difficult breathing)

At much higher and longer doses of VNS than will be used in this study (for subjects with epilepsy), some subjects have noted some influence on heart rate variability (brief changes in pulse); however, clinically relevant cardiac effects have not been observed in controlled studies. Additionally, although not shown to be definitely related to stimulation, a small number of subjects in studies of other indications using substantially higher stimulation intensity and duration have reported cardiac abnormalities after stimulation was initiated. Based on a large body of preclinical and clinical literature and our previous clinical investigations using lesser VNS stimulation that is similar to this study, cardiac effects are not expected given the substantially lower stimulation intensity and shorter duration employed in the present protocol.

Additional discussion of device removal choices and potential risks:

If adverse events are experienced, the team of surgeons and clinical investigators will determine if the device warrants removal during or at completion of the study. If device removal is warranted, the study team will make a strong recommendation to you to have it removed.

You are free to decide to have the device removed at any time during the study. Upon completion of the study, even if you have not experienced any issues with the device, it is your choice whether to leave the device implanted or have the device removed.

The risks of leaving the device implanted long-term are currently unknown.

Device removal may have increased risks compared with the original implantation surgery. This is because there may be an increased risk of damage to the structure around the device because of potential scar tissue from the original operation. In reported series removal of the implanted electrode (a different device that wrapped wires around the vagus nerve) resulted in a slightly higher incident of nerve injury compared to implantation (4.9 vs 3.6%). As explained in the

IRB NUMBER: 019-356
IRB APPROVAL DATE: 07/02/2024
IRB EXPIRATION DATE: 12/6/2024

section on the risks of implantation, damage to the nerve may result in issues such as hoarseness, difficulty with swallowing and cough.

It is recommended that you talk to your doctor to determine whether to leave the device implanted or have it removed.

If you choose to leave the device in place, you may be contacted annually by phone to check on you, and you may also be contacted about relevant future studies.

Warning: ReStore PCM should not be used in the vicinity of metal detectors, anti-theft devices and Radiofrequency Identification readers and equipment marked with the following symbol:

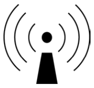

Talking about your injury/damage may be emotionally painful, and some people may experience emotional distress or thoughts of suicide in response to that pain. The research associate or the study investigators will always be available during the study to discuss with you any distress you are experiencing. A representative from the clinical team will follow-up directly with you in person or over the phone, and the study Principal Investigator (PI), to ensure proper care and follow-up occur. If we discover at any time over the course of the study that you are so emotionally upset that you are at risk of harming yourself, we may need to break confidentiality to get you the care that you need.

For more information about risks and side effects, ask one of the researchers or study staff.

We will tell you about any significant new findings which develop during the course of this research which may relate to your willingness to continue taking part.

Risks could occur if your information is released by mistake. The measures being taken to protect your privacy are taken seriously and make this possibility unlikely. However, releasing this information to you could cause psychological distress, anxiety, or family problems. Releasing this information to others, such as including it in your medical record, may pose a possible risk of discrimination, or increased difficulty in obtaining or maintaining disability, long-term care, or life insurance.

Reproductive Risks: Because the device in this study could harm an unborn baby, you should not become pregnant while on this study. Examples of acceptable birth control options include hormonal contraceptives (combined oral contraceptives, patch, vaginal ring, injectable and implants), intrauterine device (IUD), intrauterine system (IUD), vasectomy, tubal ligation, complete abstinence. You should not nurse your baby while in this study. Ask about counseling and more information about preventing pregnancy. A urine pregnancy test is not as sensitive as a blood pregnancy test and a negative urine test does not completely rule out an early pregnancy in

progress. The study device has not been adequately studied on pregnant women and the effects on the fetus/unborn child are unknown.

Conflict of Interest

Your doctor may be an investigator in this study. If so, s/he is interested both in your medical care and in the conduct of this research. Before you sign up for this study or at any time during the research, you may discuss your care with another doctor who is not associated with this research project. You are not under any obligation to take part in any research study offered by your doctor.

Researcher Financial Interests in this Study
Robert Rennaker, PhD, Michael Kilgard, PhD and Seth Hays, PhD working on this study are the inventors of the use of vagus nerve stimulation for spinal cord injury rehabilitation, for which a patent has been filed by UT Dallas. Michael Kilgard, PhD is a shareholder in and consultant for MicroTransponder, Inc. which is developing VNS therapy for stroke. Robert Rennaker, PhD is a part owner and CEO of Xnerve, which has licensed portions of the ReStore system for commercial use, as well as OptoKinetix and Konan Medical USA. These individuals may be paid for other work that is unrelated to this study.

What About Confidentiality?

You have a right to privacy. This means that all the information about you from this study will only be shown to the people working on this study. The results of this study may be published in a scientific book or journal. If this is done, your name will not be used. All information about you from this research project will be kept in a locked office or other locked area. Information that is kept on computers will be kept safe from access by people who should not see it.

The privacy law requires that Baylor Scott & White Research Institute ("BSWRI") and your doctors and other health care providers and facilities that have provided services to you, which could include doctors that work for the Scott & White Clinic, HealthTexas Provider Network or Texas Oncology, P.A., Baylor University Medical Center, Scott & White Medical Center – Temple and other health care providers depending on where you have received care (collectively, "Your Health Care Providers") get your permission before giving any of your health information to other people. There are people who need to review your information to make sure this study is done correctly. These people may look at or copy your information while they are doing this review. When you sign this form, you give permission to BSWRI and Your Health Care Providers to give other people information about your health as needed for the research project. These groups include people who work for BSWRI (including the Institutional Review Board), Baylor Scott and White Health, the US Food and Drug Administration, the Office for Human Research Protections, and the Association for the Accreditation of Human Research Protection Programs. This also includes the following groups of people who are working with the sponsor of this study: Robert Rennaker, PhD, Michael Kilgard, PhD, Seth Hays, PhD and Jane Wigginton, MD (at UT Dallas). Even though we usually remove your name from the information, the people who get this information may be able to figure out who you are. The kinds of health information that might be given to these people include results from lab tests or

IRB NUMBER: 019-356
IRB APPROVAL DATE: 07/02/2024
IRB EXPIRATION DATE: 12/6/2024

other tests like x-rays. This information might also include notes and other information in your medical records. We may ask for these notes and other information in your medical records from Your Health Care Providers. This means that the records of your care and information about you maintained by Your Health Care Providers may be given to the people mentioned above and, by signing this form, you are agreeing that Your Health Care Providers may release this information to these people. This could also be information about mental illness (except for specific notes of psychotherapy sessions). A representative from the Sponsor may be present during study procedures.

You do not have to give this permission and it is all right to refuse to sign this form. Your doctor will still treat you and your insurance company will still pay your medical bills (according to their policy) even if you do not give your permission for BSWRI and Your Health Care Providers to release this information. However, since it is important for the people listed above to have access to your information, if you do not sign this form, you cannot be in this study.

If you give permission to BSWRI and Your Health Care Providers to give other people information about your health and the other people are not part of the group that must obey the privacy law, your health information will no longer be protected by the privacy law. However, we will take all reasonable measures to protect your information from being misused.

If you change your mind and later want to withdraw your permission, you may do so. You must notify BSWRI in writing at 3434 Live Oak Street, Dallas, TX 75204. Please be sure to tell us the name of this study and the PI for this study for which you are withdrawing your permission. BSWRI will provide your withdrawal notice to Your Health Care Providers promptly after BSWRI receives your withdrawal notice. While not required, you should also talk to your PI and Your Health Care Providers and make sure they are aware you are withdrawing your permission. If you withdraw your permission, it will not apply to information that was given to others by BSWRI before you withdrew or to information given to others by Your Health Care Providers before Your Health Care Providers receive your notice withdrawing your permission. If you withdraw your permission, you will no longer be able to take part in this study.

You may not be allowed to look at your study-related health information during this study. However, at a later time, you will be able to look at this information. This later time will be sometime after this study is completed.

Unless permission is withdrawn, this permission will not expire at the end of this study.

A description of this clinical trial will be available on www.ClinicalTrials.gov, as required by U.S. Law. This website will not include information that can identify you. At most, the website will include a summary of the results. You can search this website at any time.

Study visits and rehabilitation sessions will be video recorded for training and study purposes, and portions of sessions will be reviewed live or prerecorded at a later date.

Additional Financial Information

Full Accreditation
IRB NUMBER: 019-356
IRB APPROVAL DATE: 07/02/2024
IRB EXPIRATION DATE: 12/6/2024

There will be no cost to you for being in this study. This means that you (or your insurance company) will not be billed for any of the drugs, devices, tests, or visits that take place for the study. If you see the same doctors for other clinical care, you or your insurance company will be responsible for those costs, the same as if you were not in the study.

Your payment will be made by the University of Texas at Dallas and is considered taxable income. You will be paid up to $3,500.00 for the time you spend participating in the study. You will receive $87.50 per visit for each visit after implantation plus an additional $87.50 for completion of Assessment 2 at the end of Phase 1. Compensation is provided in the form of a UT Dallas GalaxyPay card, which can be used as a credit or debit card. You must be eligible to be paid in the United States and willing to complete all the necessary tax/legal paperwork to receive this payment. Compensation will be credited to the card upon completion of each study visit. Your name, address, date of birth and social security number will be shared with a third-party solely for the purposes of compensation processing. All information will be stored in a secure fashion. Please note that if you are on record as owing money to the State of Texas, such as for back child support or a delinquent student loan, the payment may be applied to that debt. An IRS Form 1099 will be sent to you if your total payments are $600.00 or more in a calendar year unless it's a reimbursement.

What if I am Injured or Become Ill While Taking part in this Study?

The people doing this research project will do everything they can to make sure you do not get hurt during the project. If you do get hurt, there are some things that you need to know:

- Baylor Scott and White Health, Baylor Scott and White Research Institute, Baylor Scott & White Institute for Rehabilitation, and Baylor University Medical Center have not set funds aside to pay you money if you are hurt. The University of Texas at Dallas has not set funds aside to pay you money if you are hurt.
- If you have an emergency illness during the project, the people working with you will provide emergency care. You or your insurance company may need to pay for the emergency care if that happens.
- You have not given up any of your legal rights by signing this form.

The Centers for Medicare and Medicaid Services (CMS), the agency that administers the Medicare program, has stated that payments by clinical trial sponsors for injuries related to a trial are a form of liability insurance and must be reported to CMS. As a result, if an institution affiliated with this study ("Institution") pays any medical expenses to treat a trial-related injury, and if you are covered by Medicare, Institution must report that payment to CMS. In order to do that, the Institution must have certain individually identifiable information about you, such as your name, date of birth, Social Security number, Medicare claim number, date of injury and a description of the injury.

While Institution normally will not receive any individually identifiable information about you, Institution (or its delegate) will receive your individually identifiable information if (and only if) you are covered by Medicare and have incurred medical expenses that have been determined to be the result of a trial-related injury. If it receives your individually identifiable information,

IRB NUMBER: 019-356
IRB APPROVAL DATE: 07/02/2024
IRB EXPIRATION DATE: 12/6/2024

Institution (or its delegate) will only use that information to make legally required reports to CMS.

What are My Rights as a Subject?

Taking part in this study is voluntary. You may choose not to take part or may leave this study at any time. If you agree to take part and then decide against it, you can withdraw for any reason. At certain times during the treatment, it may be unsafe for you to withdraw, so please be sure to discuss leaving this study with the PI or your regular doctor. Deciding not to be in this study, or leaving this study early, will not result in any penalty or loss of benefits that you would otherwise receive.

We will tell you about any new information that may affect your health, welfare, or willingness to stay in this study.

Whom Do I Call If I have Questions or Problems?

If you have concerns, complaints, or questions about this study or have a research-related injury, contact Rita Hamilton, DO at 214-820-8557 or Jane Wigginton, MD at 469-648-8571.

For concerns, complaints, or questions about your rights as a research subject or if you simply wish to speak with someone who is not a part of the research staff, contact the IRB Office at 254-215-9697.

PERMISSION TO BE CONTACTED REGARDING FUTURE TRIALS
As a progressive research program there may be future trials that may be based on or/and related to the outcomes of this study. We would love to contact you in future regarding any future trials for people with stroke as they might be helpful or of interest to you.

___ I do not wish to be contacted about future studies.

PERMISSION TO OBTAIN INFORMATION FROM ADDITIONAL SOURCES
If the study site is unable to contact me after repeated attempts at any point during the study, I authorize the study site to contact my personal doctor and family member or friend to obtain information about how to contact me and to learn about changes in my health. I also authorize the site to use public records to find information that can help them to contact me.

Name of personal doctor: ____
Phone number: ________________________

Name of friend/family member:____
Phone number: ________________________
Relationship: __________________________

___ I do not wish to provide this information or allow these methods to be used to contact me.

Full Accreditation
IRB NUMBER: 019-356
IRB APPROVAL DATE: 07/02/2024
IRB EXPIRATION DATE: 12/6/2024

Statement of Person Obtaining Consent:

I have explained to ____the purpose of this study, the procedures required and the possible risks and benefits to the best of my ability. They have been encouraged to ask questions related to taking part in this study. I gave a copy of this consent to the subject.

_______________________________________ ___________________ _____________
Signature of Person Obtaining Consent Date Time

Confirmation of Consent by Research Subject:

You are making a decision about being in this study. You will be asked to give your written consent if you want to be in this study. Giving consent is like giving permission. You should not give your permission to be in this study until you have read and understood all pages in this form. Make sure that all your questions about this study have been answered before you sign this form. When you sign this form, you are giving your permission to be in the study. By signing this form, you have not given up any of your legal rights or released anyone from liability for negligence.

_________________________________ has explained to me the purpose of this study, the study procedures that I will have, and the possible risks and discomforts that may happen. I have read (or have been read) this consent form. I have been given a chance to ask questions about this study and the procedures involved. I believe that I have enough information to make my decision. I have also been told my other options. To the best of my knowledge, I am not in any other medical research. Therefore, I consent to take part as a subject in this study and authorize the activities described in this consent. I also acknowledge that I have received a copy of this consent form.

______________________________________ ___________________ ____________
Signature of Subject Date Time

I understand that this individual is physically not able to sign the document. However, I have been present during the entire informed consent discussion and my signature indicates that the individual understands the information as presented and agrees to take part in the study.

_________________________________ ___________________ ____________
Signature of Witness to Entire Process Date Time

IRB NUMBER: 019-356
IRB APPROVAL DATE: 07/02/2024
IRB EXPIRATION DATE: 12/6/2024

Participant ID#__________________________

Date of Visit: __ __/__ __/__ __ __ __

Visit # _________ Study # _____________

IRB # _______________

Informed Consent Documentation/Process

Documentation of Informed Consent Process

___ History reviewed for inclusion / exclusion criteria

___ Participant seen by delegation of principal investigator

___ Information about study including all options was provided in a language understood by the participant

___Consents reviewed with participant / Witness / Legally Authorized Representative

___ HIPAA information reviewed with participant / Witness / Legally Authorized Representative

___ Participant was given adequate opportunity to consider all available options

___ All questions / concerns were answered / addressed

___ Participant / Witness / Legally Authorized Representative communicated comprehension of information

___ Participant / Witness / Legally Authorized Representative agreed to participate in study

___ Consents signed and dated by all parties prior to any study specific procedure

___ Copy of signed consent was given to participant

___ BSWH Authorization Use and Disclosure of Protected Health Information

___ UTD Talent Release Form

___UTSW Photograph Consent and Release Form

Date consent was signed: ______________________ Time Signed: _______________________

___________________________	______________________________	______/ ______/ ______	______
Name of Person Obtaining Consent	Signature of Person Obtaining Consent	MM DD YYYY	Time

Participant ID# ____________________________

Date of Visit: __ __/__ __/__ __ __ __

Revision History

Revision	Date	Reason	ECN #
A	3/01/2019	Initial Release	TxBDC is introducing this form for the first time: ECN 1028
B	11/15/2019	Edits for IRB Submission	1062
B.1	9/30/2020	Edits for Photo and Talent Releases, added name field	N/A
C	02/01/2021	Rev B.1 was reviewed and approved by participating IRBs and FDA. Rev C is now being released via change control process. No changes made between B.1 and Rev C	1132
C.1	5/24/21	Remove BSW non-patient consent to photograph and quote. Form is extraneous as all trial participants will be BSW patients. Added IRB number to header for clarity.	N/A
D	08/05/2021	Rev C.1 was reviewed and approved by participating IRBs and FDA. Rev D is now being released via change control process. No changes made between Rev C.1 and Rev D.	1180
D.1	10/28/21	Added Witness to list of individuals who could sign the informed consent form to align process with actual informed consent signature lines.	N/A
E		Rev D.1 was reviewed and approved by participating IRBs and FDA. Rev E is now being released via change control process. No changes made between Rev D.1 and Rev E.	1215
