# Supplementary material for: Closed-loop vagus nerve stimulation aids recovery from spinal cord injury
Source: Nature. 2025 May 21;643(8073):1030–6. doi: 10.1038/s41586-025-09028-5 (PMC12286844; doi:10.1038/s41586-025-09028-5)
Supplement: Supplementary file 2 — Reporting Summary [file 41586_2025_9028_MOESM2_ESM.pdf]

nature portfolio

Corresponding author(s): Michael Kilgard

Last updated by author(s): Mar 21, 2025

Reporting Summary

Nature Portfolio wishes to improve the reproducibility of the work that we publish. This form provides structure for consistency and transparency in reporting. For further information on Nature Portfolio policies, see our Editorial Policies and the Editorial Policy Checklist.

Statistics

For all statistical analyses, confirm that the following items are present in the figure legend, table legend, main text, or Methods section.

n/a	Confirmed	
☐	☒	The exact sample size (*n*) for each experimental group/condition, given as a discrete number and unit of measurement
☐	☒	A statement on whether measurements were taken from distinct samples or whether the same sample was measured repeatedly
☐	☒	The statistical test(s) used AND whether they are one- or two-sided<br>*Only common tests should be described solely by name; describe more complex techniques in the Methods section.*
☐	☒	A description of all covariates tested
☐	☒	A description of any assumptions or corrections, such as tests of normality and adjustment for multiple comparisons
☐	☒	A full description of the statistical parameters including central tendency (e.g. means) or other basic estimates (e.g. regression coefficient) AND variation (e.g. standard deviation) or associated estimates of uncertainty (e.g. confidence intervals)
☐	☒	For null hypothesis testing, the test statistic (e.g. *F*, *t*, *r*) with confidence intervals, effect sizes, degrees of freedom and *P* value noted<br>*Give P values as exact values whenever suitable.*
☒	☐	For Bayesian analysis, information on the choice of priors and Markov chain Monte Carlo settings
☒	☐	For hierarchical and complex designs, identification of the appropriate level for tests and full reporting of outcomes
☐	☒	Estimates of effect sizes (e.g. Cohen's *d*, Pearson's *r*), indicating how they were calculated

Our web collection on statistics for biologists contains articles on many of the points above.

Software and code

Policy information about availability of computer code

Data collection	https://github.com/davepruitt/RePlay
Data analysis	Data analysis was performed using standard tools in MATLAB.

For manuscripts utilizing custom algorithms or software that are central to the research but not yet described in published literature, software must be made available to editors and reviewers. We strongly encourage code deposition in a community repository (e.g. GitHub). See the Nature Portfolio guidelines for submitting code & software for further information.

Data

Policy information about availability of data

All manuscripts must include a data availability statement. This statement should provide the following information, where applicable:

- Accession codes, unique identifiers, or web links for publicly available datasets
- A description of any restrictions on data availability
- For clinical datasets or third party data, please ensure that the statement adheres to our policy

Data is available at Open Data Commons for Spinal Cord Injury (https://odc-sci.org/). https://doi.org/10.34945/F5302810.34945/F5G30Z

Research involving human participants, their data, or biological material

Policy information about studies with human participants or human data. See also policy information about sex, gender (identity/presentation), and sexual orientation and race, ethnicity and racism.

Reporting on sex and gender	Data was collected in both sexes.
Reporting on race, ethnicity, or other socially relevant groupings	Self-reported race and ethnicity are reported as per U.S. Food and Drug Administration guidelines.
Population characteristics	Population characteristics are reported in the manuscript. This study reports a clinical trial, and no participant characteristics were restricted beyond those defined in the inclusion/exclusion criteria.
Recruitment	Potential participants were identified through an established referral network at Baylor Scott & White Spinal Cord Injury Model System, study flyers, local advertisements, and online advertisement.
Ethics oversight	Regulatory and ethics oversight for this trial was provided by the FDA, the IRB at Baylor Scott & White, the IRB at the University of Texas at Dallas, and the Department of Defense Human Research Protection Office (DOD HRPO).

Note that full information on the approval of the study protocol must also be provided in the manuscript.

Field-specific reporting

Please select the one below that is the best fit for your research. If you are not sure, read the appropriate sections before making your selection.

☒ Life sciences ☐ Behavioural & social sciences ☐ Ecological, evolutionary & environmental sciences

For a reference copy of the document with all sections, see nature.com/documents/nr-reporting-summary-flat.pdf

Life sciences study design

All studies must disclose on these points even when the disclosure is negative.

Sample size	Maximum sample size was limited to 20 by the U.S. FDA for this first in human study.
Data exclusions	No data was excluded.
Replication	Pearson correlation analysis was used to evaluate test-retest reliability.
Randomization	A blocked software-randomized design was used with a block size of two. The blocking covariate was impairment severity (Treated Arm GRASSP ≤58 vs >58).
Blinding	Participants, therapists, and investigators were blinded to treatment allocation throughout the double-blinded portion of the study.

Reporting for specific materials, systems and methods

We require information from authors about some types of materials, experimental systems and methods used in many studies. Here, indicate whether each material, system or method listed is relevant to your study. If you are not sure if a list item applies to your research, read the appropriate section before selecting a response.

Materials & experimental systems

n/a	Involved in the study	
☒	☐	Antibodies
☒	☐	Eukaryotic cell lines
☒	☐	Palaeontology and archaeology
☒	☐	Animals and other organisms
☐	☒	Clinical data
☒	☐	Dual use research of concern
☒	☐	Plants

Methods

n/a	Involved in the study	
☒	☐	ChIP-seq
☒	☐	Flow cytometry
☒	☐	MRI-based neuroimaging

Clinical data

Policy information about clinical studies

All manuscripts should comply with the ICMJE guidelines for publication of clinical research and a completed CONSORT checklist must be included with all submissions.

Clinical trial registration	NCT04288245, https://clinicaltrials.gov/study/NCT04288245
Study protocol	The full study protocol is available to the editors and reviewers. A summary is available on clinicaltrials.gov.
Data collection	Data was collected at the Baylor Scott & White Spinal Cord Injury Model System in Dallas, Texas. Participants were recruited from March 5, 2021 to June 30, 2023.
Outcomes	Blinded therapists assessed each participant. Primary and secondary outcomes were preregistered on clinicaltrials.gov.

Plants

Seed stocks	n/a
Novel plant genotypes	n/a
Authentication	n/a
